# Safety of a controlled human infection model of tuberculosis with aerosolised, live-attenuated *Mycobacterium bovis* BCG versus intradermal BCG in BCG-naive adults in the UK: a dose-escalation, randomised, controlled, phase 1 trial

**DOI:** 10.1016/S1473-3099(24)00143-9

**Published:** 2024-04-12

**Authors:** Iman Satti, Julia L Marshall, Stephanie A Harris, Rachel Wittenberg, Rachel Tanner, Raquel Lopez Ramon, Morven Wilkie, Fernando Ramos Lopez, Michael Riste, Daniel Wright, Marco Polo Peralta Alvarez, Nicola Williams, Hazel Morrison, Elena Stylianou, Pedro Folegatti, Daniel Jenkin, Samantha Vermaak, Linnea Rask, Ingrid Cabrera Puig, Rebecca Powell Doherty, Alison Lawrie, Paul Moss, Timothy Hinks, Henry Bettinson, Helen McShane

**Affiliations:** The Jenner Institute; Nuffield Department of Primary Care Health Sciences; The Jenner Institute; University of Oxford, Oxford, UK; Institute of Immunology and Immunotherapy, University of Birmingham, Birmingham, UK; Oxford Centre for Respiratory Medicine, Nuffield Department of Clinical Medicine; The Jenner Institute

## Abstract

**Background:**

*Mycobacterium tuberculosis* is the main causative agent of tuberculosis. BCG, the only licensed vaccine, provides inadequate protection against pulmonary tuberculosis. Controlled human infection models are useful tools for vaccine development. We aimed to determine a safe dose of aerosol-inhaled live-attenuated *Mycobacterium bovis* BCG as a surrogate for *M tuberculosis* infection, then compare the safety and tolerability of infection models established using aerosol-inhaled and intradermally administered BCG.

**Methods:**

This phase 1 controlled human infection trial was conducted at two clinical research facilities in the UK. Healthy, immunocompetent adults aged 18–50 years, who were both *M tuberculosis*-naive and BCG-naive and had no history of asthma or other respiratory diseases, were eligible for the trial. Participants were initially enrolled into group 1 (receiving the BCG Danish strain); the trial was subsequently paused because of a worldwide shortage of BCG Danish and, after protocol amendment, was restarted using the BCG Bulgaria strain (group 2). After a dose-escalation study, during which participants were sequentially allocated to receive either 1 × 10^3^, 1 × 10^4^, 1 × 10^5^, 1 × 10^6^, or 1 × 10^7^ colony-forming units (CFU) of aerosol BCG, the maximum tolerated dose was selected for the randomised controlled trial. Participants in this trial were randomly assigned (9:12), by variable block randomisation and using sequentially numbered sealed envelopes, to receive aerosol BCG (1 × 10^7^ CFU) and intradermal saline or intradermal BCG (1 × 10^6^ CFU) and aerosol saline. Participants were masked to treatment allocation until day 14. The primary outcome was to compare the safety of a controlled human infection model based on aerosol-inhaled BCG versus one based on intradermally administered BCG, and the secondary outcome was to evaluate BCG recovery in the airways of participants who received aerosol BCG or skin biopsies of participants who received intradermal BCG. BCG was detected by culture and by PCR. The trial is registered at ClinicalTrials.gov, NCT02709278, and is complete.

**Findings:**

Participants were assessed for eligibility between April 7, 2016, and Sept 29, 2018. For group 1, 15 participants were screened, of whom 13 were enrolled and ten completed the study; for group 2, 60 were screened and 33 enrolled, all of whom completed the study. Doses up to 1 × 10^7^ CFU aerosol-inhaled BCG were sufficiently well tolerated. No significant difference was observed in the frequency of adverse events between aerosol and intradermal groups (median percentage of solicited adverse events per participant, post-aerosol *vs* post-intradermal BCG: systemic 7% [IQR 2–11] *vs* 4% [1–13], p=0·62; respiratory 7% [1–19] *vs* 4% [1–9], p=0·56). More severe systemic adverse events occurred in the 2 weeks after aerosol BCG (15 [12%] of 122 reported systemic adverse events) than after intradermal BCG (one [1%] of 94; difference 11% [95% CI 5–17]; p=0·0013), but no difference was observed in the severity of respiratory adverse events (two [1%] of 144 *vs* zero [0%] of 97; 1% [−1 to 3]; p=0·52). All adverse events after aerosol BCG resolved spontaneously. One serious adverse event was reported—a participant in group 2 was admitted to hospital to receive analgesia for a pre-existing ovarian cyst, which was deemed unrelated to BCG infection. On day 14, BCG was cultured from bronchoalveolar lavage samples after aerosol infection and from skin biopsy samples after intradermal infection.

**Interpretation:**

This first-in-human aerosol BCG controlled human infection model was sufficiently well tolerated. Further work will evaluate the utility of this model in assessing vaccine efficacy and identifying potential correlates of protection.

**Funding:**

Bill & Melinda Gates Foundation, Wellcome Trust, National Institute for Health Research Oxford Biomedical Research Centre, Thames Valley Clinical Research Network, and TBVAC2020.

## Introduction

More than 1·13 million people worldwide died from tuberculosis in 2022.^1^ The only licensed vaccine, BCG, confers inadequate protection against adult pulmonary disease in endemic areas.^2^

The efficacy of candidate tuberculosis vaccines requires evaluation in large, expensive randomised controlled trials. A controlled human infection model would enable the rational selection of candidate vaccines for field efficacy trials, reducing the financial risks of vaccine development. Such models can also identify potential correlates of protection, which can be validated in samples from human trials.^3^

Deliberately infecting humans with virulent *Mycobacterium tuberculosis* would be unethical. BCG, a live-attenuated *Mycobacterium bovis* strain that can cause tuberculosis in humans, could be used as a surrogate for *M tuberculosis* in a controlled human infection model.^4,5^ We have previously developed such a model using intradermal BCG.^4,5^ Studies of this model in the UK, where BCG has been shown to protect against tuberculosis, showed that historic BCG vaccination reduced mycobacterial recovery after a subsequent intradermal BCG challenge.^4,5^ The natural route of *M tuberculosis* infection is through the inhalation of aerosolised infectious droplets. Because intradermal BCG infection might induce a different immune response to a lung infection, the delivery of BCG by inhalation would better model the mucosal response to *M tuberculosis* infection.^6^ A study in South Africa found that instillation of BCG into the lung segments of BCG-vaccinated adult participants who were exposed to *M tuberculosis* was well tolerated and induced localised cellular, humoral, and transcriptomic responses.^7^ Delivering BCG to the respiratory mucosa of non-human primates was significantly more protective than intradermal vaccination against *M tuberculosis* challenge.^8,9^ To better mimic the natural route of *M tuberculosis* infection, we aimed to establish the first human study to deliver BCG as an infectious challenge via the aerosolised route in healthy *M tuberculosis*-naive and BCG-naive adults.

## Methods

### Study design

This controlled human infection trial consisted of a dose-escalation trial followed by a single-blind, randomised, controlled, phase 1 trial. In the dose-escalation trial, we assessed five doses of aerosol BCG (1 × 10^3^, 1 × 10^4^, 1 × 10^5^, 1 × 10^6^, and 1 × 10^7^ colony-forming units [CFU]). Safety data were reviewed by the Chief Investigator (HMc) before dose escalation. A local safety committee, comprising independent infectious disease physicians, provided oversight. The maximum tolerated dose of aerosol BCG was taken forward into the randomised trial comparing aerosol BCG with intradermal BCG. The study commenced using BCG Danish (group 1), the licenced BCG strain in the UK. However, because of a global shortage of BCG Danish in 2016, the protocol was amended to use BCG Bulgaria (group 2), the strain used in the National Immunisation Programme in England during the BCG Danish shortage.

The trial was conducted at the Centre for Clinical Vaccinology and Tropical Medicine, University of Oxford (Oxford, UK) and the National Institute of Health Research/Wellcome Trust Birmingham Clinical Research Facility (Birmingham, UK). The trial protocol and associated documents were approved by the Medicines and Healthcare products Regulatory Agency (EudraCT: 2015-004981-27) and the South-Central Oxford A Research Ethics Committee (15/SC/0716). The trial was conducted according to the Declaration of Helsinki and Good Clinical Practice. The trial protocol can be found in the appendix (pp 39–77).

### Participants

Participants were recruited using approved advertisements on posters, in newsletters, and on Facebook, and were screened for eligibility after providing written informed consent. Eligible participants were healthy, immunocompetent adults aged 18–50 years, had no history of asthma or other respiratory diseases, and were *M tuberculosis*-naive (IFNγ release assay negative and no known history of tuberculosis) and BCG-naive (no BCG scar and no BCG vaccine history). A tuberculin skin test was not used to detect historic BCG vaccination to avoid immune priming and because of the poor sensitivity and specificity of the test. Biological sex (male or female) was self-reported. For a full list of inclusion and exclusion criteria, see appendix (pp 21–22).

### Randomisation and masking

All participants were enrolled by trial investigators. For safety purposes, participants in the dose-escalation groups (group 1A, group 1B, one participant in group 1C, and groups 2A–D) were sequentially enrolled and were not masked to group assignment (appendix p 11).

Allocation into masked comparator groups (ie, to receive either aerosol or intradermal BCG) was by variable block randomisation using sequentially numbered, sealed, opaque envelopes, prepared by an independent statistician at the Centre for Statistics in Medicine, University of Oxford (Oxford, UK). Participants in randomly assigned groups were masked using a paired-placebo design. In brief, those who were randomly assigned to inhale aerosol BCG (groups 1C and 2E) received a concurrent intradermal saline injection, and those who were to receive intradermal BCG (groups 1D and 2F) inhaled saline. Because BCG doses were prepared in saline, both BCG and placebo doses looked and tasted the same. Participants were masked until day 14, when those who had received intradermal BCG had a skin biopsy. Three participants in group 2E were unmasked at day 2, as per protocol, when sputum was induced. The bronchoscopist and senior immunologist were masked to group allocation throughout.

### Procedures

Dose escalation using aerosol BCG Danish was completed to the target of 1 × 10^5^ CFU, and the randomised trial began; however, this phase was not completed owing to the 2016 global shortage of BCG Danish. Because the safety profile of aerosol BCG Danish (1 × 10^3^–1 × 10^5^ CFU) was acceptable, we commenced dose escalation with BCG Bulgaria at 1 × 10^4^ CFU. To maximise BCG recovery (as part of the secondary objectives of the trial), we escalated the dose to 1 × 10^7^ CFU.

BCG Danish vials (Statens Serum Institute; Copenhagen, Denmark) contain 2–8 × 10^6^ CFU and BCG Bulgaria vials (InterVax; Markham, ON, Canada) contain 1·5–6 × 10^6^ CFU. Medians of 5 × 10^6^ CFU (BCG Danish, group 1) and 4 × 10^6^ CFU (BCG Bulgaria, group 2) per vial were assumed for dosing calculations. Both strains were supplied as a lyophilised powder with solvent for reconstitution.

BCG was reconstituted, then either diluted with sterile 0·9% NaCl or concentrated by combining vials to generate the required dose. For participants receiving intradermal BCG (group 1D, BCG Danish; group 2F, BCG Bulgaria), 100 μL of the appropriate dose was given by intradermal injection over the deltoid muscle. For those receiving aerosol BCG, 0·9% NaCl was combined with the appropriate dose of BCG to a volume of 1 mL and delivered using the Omron MicroAir U22 ultrasonic mesh nebuliser (Omron Healthcare, Milton Keynes, UK).

Participants received one dose of either aerosol (1 × 10^3^−1 × 10^7^ CFU) or intradermal (1 × 10^5^−1 × 10^6^ CFU) BCG; those in the masked comparator groups also received intradermal or aerosol saline. Expected respiratory adverse events (ie, cough, sore throat, wheeze, dyspnoea, sputum production, haemoptysis, and chest pain) and systemic adverse events (ie, fever [>37·5°C], feverishness, fatigue, malaise, headache, myalgia, arthralgia, and nausea) were solicited using an electronic diary card for 28 days after BCG infection and reviewed at clinic visits. Unsolicited adverse events, which included any events associated with intradermal administration, were collected throughout the trial period. Stopping criteria were any related serious adverse event or two or more participants having the same related grade 3 adverse event beginning within 2 days after challenge and persisting for more than 48 h. Participants with fever or any grade 2 adverse event attended the relevant centre for investigation, which included a nasopharyngeal swab for respiratory viral PCR screen and blood cultures, including mycobacterial detection. An electrocardiogram was obtained from any participant with chest pain or tightness. Participants were referred for further assessment to a specialist at Oxford University Hospitals NHS Foundation Trust (Oxford, UK) as required.

Blood was collected at every scheduled visit (days 0, 2, 7, 14, 28, 84, and 168; appendix p 11). The urine of all female participants was analysed for β-human chorionic gonadotropin on the day of BCG infection (day 0) and the day of bronchoscopy (day 14). For participants from the Oxford centre who received aerosol BCG, induced sputum was collected at day 168 and at either day 2 and day 7 or at day 28 and day 84 (appendix pp 3, 51, 64).

Fibre optic bronchoscopy was conducted on all participants 14 days after BCG infection, using a standardised clinical protocol.^10,11^ Intravenous sedation (midazolam and fentanyl) was offered, and local anaesthetic spray was administered. The macroscopic appearance of the airways was reported and photographed. Bronchoalveolar lavage samples were collected from the right middle lobe (medial segment) using 100 mL of 0·9% NaCl.

Blood biochemical and haematological parameters were measured on days 2, 7, 14, and 28. Participants used a digital thermometer and a handheld spirometer (Micro Spirometer, CareFusion; San Diego, CA, USA) for daily home measurement for 28 days after BCG infection. Vital signs and spirometry were measured at all clinic visits.

BCG was quantified from vaccine vials after reconstitution, after dose preparation, after nebulisation, from skin biopsy and bronchoalveolar lavage samples obtained 2 weeks after infection, and from sputum collected according to the previously mentioned schedule (appendix pp 3–4).

At each scheduled clinic visit, we investigated local and systemic responses considered of importance in tuberculosis immunology: the frequencies of IFNγ^+^, TNFα^+^, IL-2^+^, and IL-17^+^ Th1 and Th17 cells and antibody concentrations (IgG and IgA; appendix pp 4–5).

### Outcomes

All outcome measures were assessed at The Jenner Institute, University of Oxford (Oxford, UK). Determination of a safe aerosol BCG dose, with which to compare the safety and tolerability of aerosol and intradermal BCG controlled human infection models, was the primary outcome. Solicited systemic adverse events were predefined from the summary of product characteristics. Solicited respiratory adverse events were determined from those reported during previous aerosol vaccine studies.^10,11^

The secondary outcome was the detection of BCG in the airways 2 weeks after aerosol infection or in skin biopsy samples 2 weeks after intradermal infection. The BACTEC Mycobacteria Growth Indicator Tube (MGIT; Wokingham, UK) system was used for BCG quantification. Genotyping was conducted on any MGIT-positive samples using the HAIN GenoType MTBC VER 1.X kit (Bruker; Nehren, Germany**;** appendix pp 3–4). The evaluation of lung mucosal and systemic immune responses induced by aerosol or intradermal infection were exploratory outcomes.

### Statistical analysis

Participant numbers were sufficient for the safety monitoring of dose escalation only in all group 1 groups and in the group 2 dose-escalation groups. This is an exploratory safety trial with descriptive endpoints. The target enrolment was initially 30 participants; however, in the amended protocol, target recruitment numbers for the BCG Danish group (group 1) were revised to reflect the actual numbers enrolled and this group was closed to enrolment; after cessation of this group and inclusion of group 2, the target enrolment was 46 participants. The sample size of 12 per intervention group was determined on the basis of our previous experience with phase 1 clinical studies and chosen with the aim of detecting substantial differences in the primary outcome measure between the two experimental groups, while also being a feasible number to enrol. The sample size was not determined with the aim of achieving statistical significance.

We compared safety endpoints between participants who received the maximum dose of aerosolised BCG Bulgaria (groups 2D and 2E) and those who received intradermal BCG Bulgaria (group 2F). Because the three participants in group 2D were not masked or randomly assigned, a comparison between the safety data of groups 2D and 2E is included as a post-hoc analysis.

Solicited adverse events were reported as a proportion of total solicited events, by frequency and maximum severity by individual or by group overall, or by frequency and length, and were analysed over the following time periods: 72 h after BCG administration, 2 weeks pre-bronchoscopy, 2 weeks after bronchoscopy, and the entire 28-day diary card.

Statistical analysis was done using GraphPad Prism (version 9) or Stata (version 14) for the area under the curve (AUC) analysis. Prism calculates exact p values, considering tied values. Non-parametric tests were applied as data were not normally distributed. For continuous data (time-to-positivity, enzyme-linked immunosorbent spot assay, flow cytometry, and antibody concentrations), differences in medians between two groups were calculated with Mann–Whitney *U* test for unpaired data or Wilcoxon signed rank test for paired data. Correction for multiple comparisons was done using the Friedman test or Dunn’s multiple comparisons test. Significant differences in medians (all clinical and immunology continuous data) between timepoints or groups are presented with p values and IQRs. For binary data outcomes (severe adverse event data in groups 2D/E and 2F), we used Fisher’s exact test. The significance level was set as p<0·05, with the caveat that the sample size limited our ability to detect differences. An AUC analysis of deviation from baseline was used to compare overall spirometry values between groups after infection. Analysis included data collected at all timepoints unless otherwise stated.

The trial was registered at ClinicalTrials.gov, NCT02709278.

### Role of the funding source

The funders of the study had no role in study design, data collection, data analysis, data interpretation, or writing of the report.

## Results

**Figure 1:**
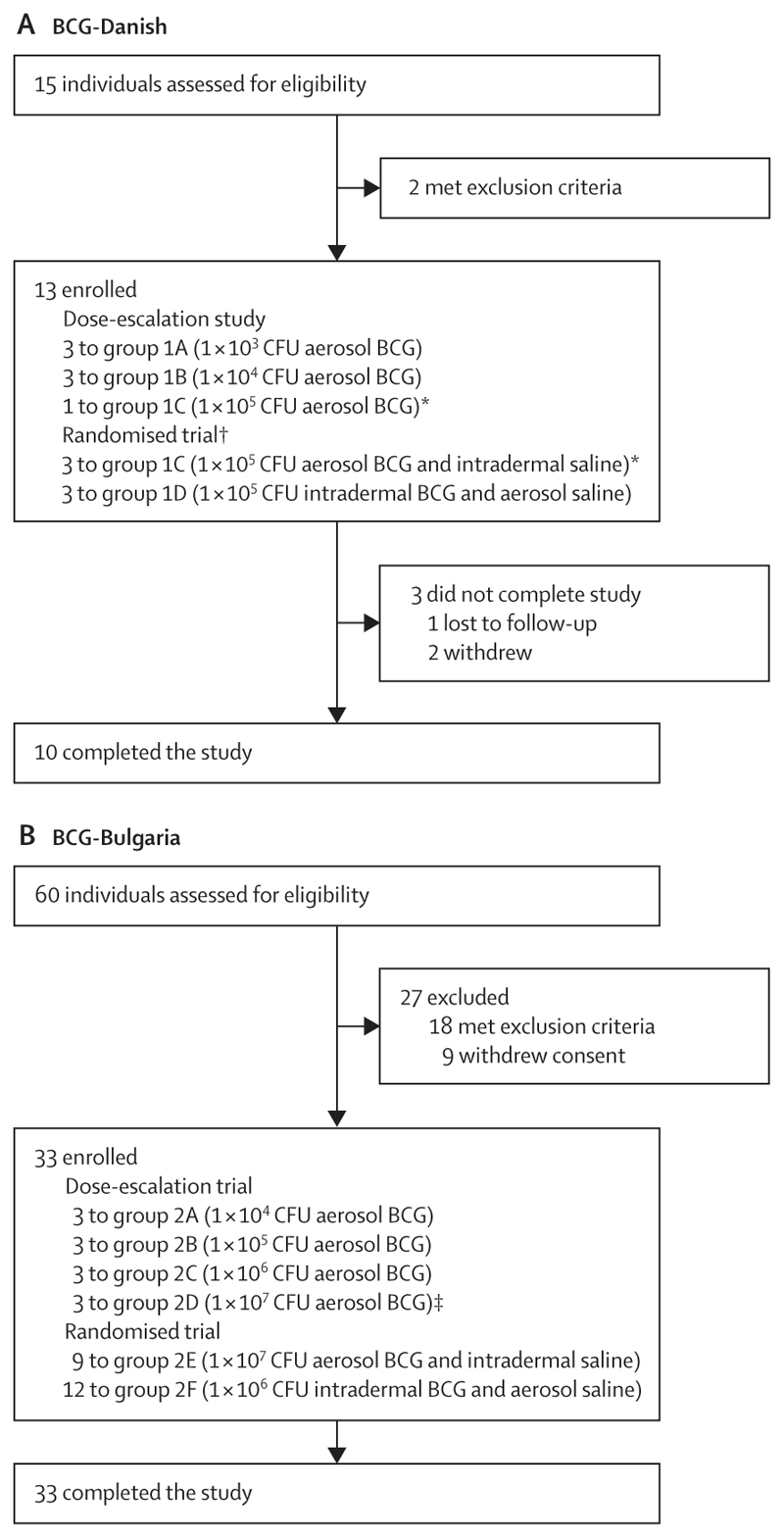
Trial profile (A) Trial profile for group 1 (BCG Danish). (B) Trial profile for group 2 (BCG Bulgaria). *The first participant in group 1C was sequentially enrolled as part of the dose-escalation study and not randomly assigned; this participant was included in the comparison of safety between aerosol BCG and intradermal BCG. †The target for enrolment into groups 1C and 1D was 12 participants per group but was incomplete owing to a shortage of BCG Danish. ‡Group 2D was enrolled as part of the dose-escalation trial but was included in the comparison between aerosol and intradermal BCG.

The first participants were enrolled on April 20, 2016, and the last participant on Oct 30, 2018. The trial was completed after the protocol-defined follow-up visits, with the last participant visit on April 16, 2019. The trial was fully enrolled, with 46 participants after protocol amendment (13 in group 1; 33 in group 2; figure 1, appendix p 11). 17 protocol deviations were reported during the trial (appendix pp 23–24); none had any clinically significant effect on participant safety or on the data generated.

Throughout the trial there were no suspected unexpected serious adverse reactions, no withdrawals due to safety concerns, and no activation of predefined stopping rules. 15 participants were screened for group 1 (BCG Danish); of these, two met exclusion criteria and 13 were enrolled. Three participants in this group withdrew during the study (figure 1A). The baseline characteristics of this group are summarised in table 1.

**Figure 2:**
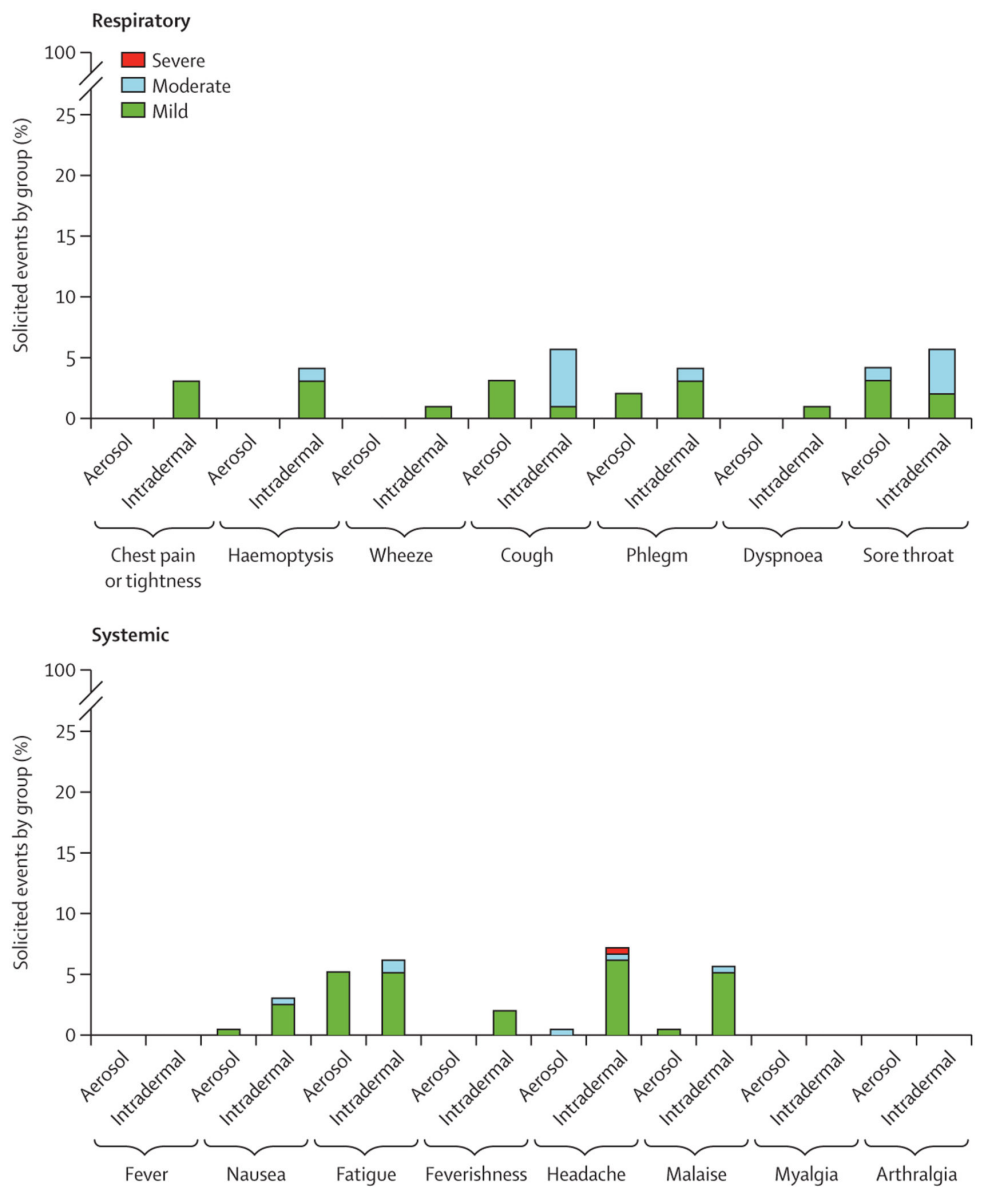
Adverse events in group 1 (BCG Danish) Occurrences of respiratory (top) and systemic (bottom) adverse events during the 2 weeks after infection with BCG Danish and before bronchoscopy. Shown as a percentage of total solicited occurrences, for group 1C (n=4; 1 × 10^5^ CFU aerosol BCG) and group 1D (n=3; 1 × 10^5^ CFU intradermal BCG), calculated as the number of participants × 16 timepoints (group 1C adjusted to 15·75 timepoints as one participant had their bronchoscopy on day 13). Adverse events were collected every 12 h for 2 days after infection and then daily.

No serious adverse events were recorded in group 1. Most symptoms by either infection route were mild and all were transient (figure 2). One participant reported a severe headache following the receipt of intradermal BCG, accompanied by feverishness, fatigue, malaise, and nausea. The headache resolved to mild within 24 h without the need for analgesia. The only treatment required by group 1 participants was over-the-counter medication for symptomatic relief. Peripheral oxygen saturation was normal at all clinic visits. All bronchoscopies in group 1 showed macroscopically normal results and no procedural complications were reported.

One participant who received aerosol BCG (group 1B; 1 × 10^4^ CFU) developed a left-middle-lobe pneumonia 2 days after bronchoscopy, which did not require hospital admission nor absence from work. Bronchoalveolar lavage and blood samples were culture-negative, including for mycobacteria. The pneumonia was deemed possibly secondary to the bronchoscopy. Symptoms rapidly and fully resolved with oral co-amoxiclav and clarithromycin (appendix p 25).

**Figure 3:**
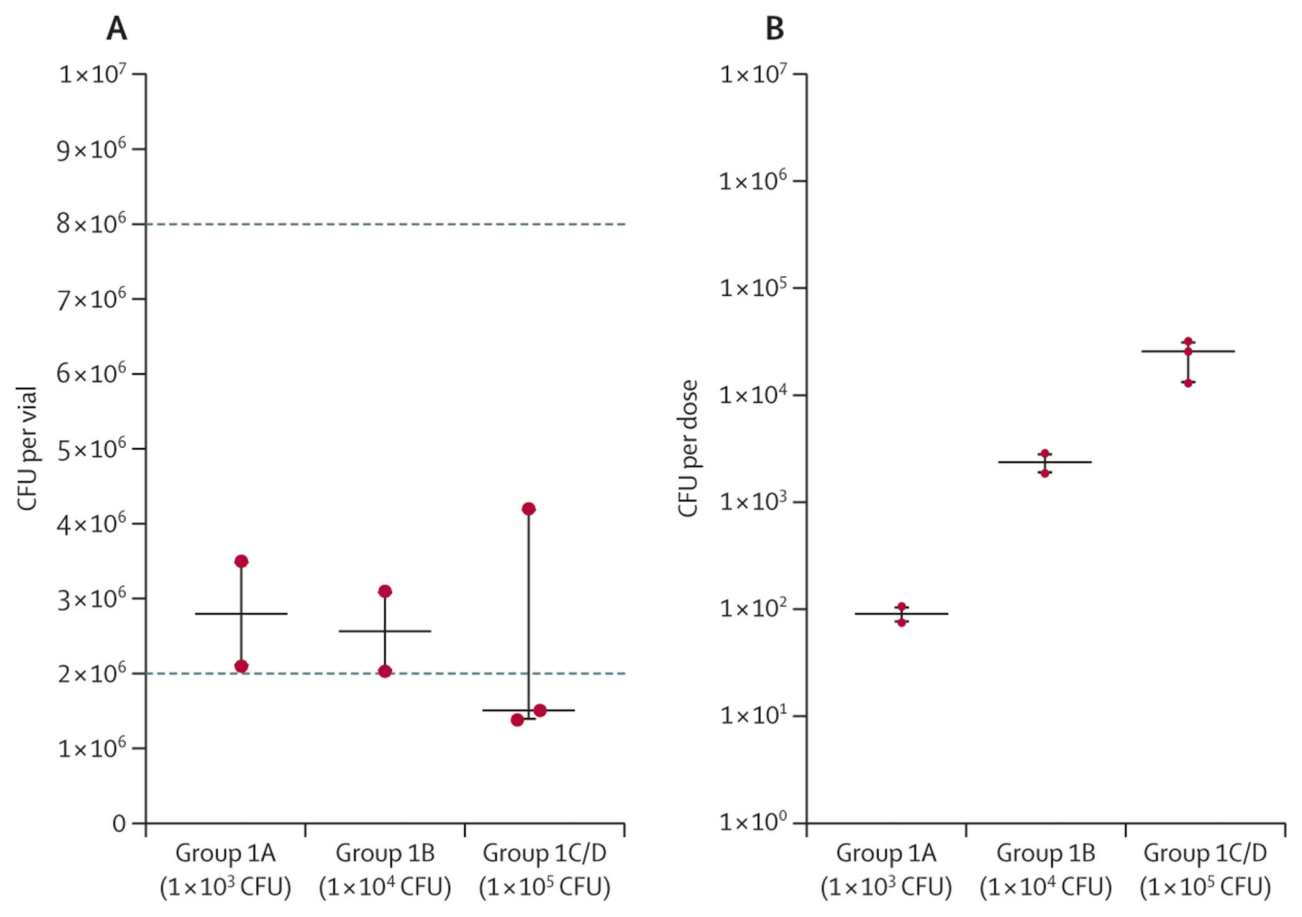
BCG quantification in group 1 (BCG Danish) (A) BCG recovered from the vaccine vials, plated onto Middlebrook 7H11 agar. The range of CFU as stated by the manufacturer is represented by the dotted lines (2–8 × 10^6^ CFU). The dots represent individual vials. (B) CFU of BCG per dose, determined from the diluted or concentrated vials after participant infection, plated onto Middlebrook 7H11 agar. Dots represent each reconstituted vial (prepared dose) (participants infected on the same day could have been infected with BCG from the same vial). The middle horizontal solid lines represent the median, and the outer lines show the upper and lower limits of the IQR and range. CFU=colony-forming units.

BCG Danish quantified from group 1 vaccine vials before dose preparation was at the lower end of the manufacturer’s stated range (figure 3A). Dose preparation achieved a log-fold increase per dose-escalation group, as planned, but was below the target dose for each group (figure 3B). No BCG was grown from the 10 mL bronchoalveolar lavage sample that was allocated for BCG detection in group 1. One of the three skin biopsies was collected at day 28 rather than day 14, as the bronchoscopy visit for this patient was deferred because of an unrelated upper respiratory tract infection. No BCG was recovered from this sample; the day-14 biopsy samples from the other two participants yielded 100 CFU and 198 CFU BCG.

60 participants were screened for group 2 (BCG Bulgaria), of whom 27 met exclusion criteria or withdrew consent before enrolment and 33 were enrolled (figure 1B). Exclusion reasons included previous BCG vaccination, pre-existing respiratory disease (eg, asthma), poor baseline lung function, and blood dyscrasias (anaemia or increased concentrations of liver enzymes). The baseline characteristics of this group are summarised in table 1.

One serious adverse event was reported in group 2E: hospital admission for analgesia for a pre-existing ovarian cyst.

**Figure 4:**
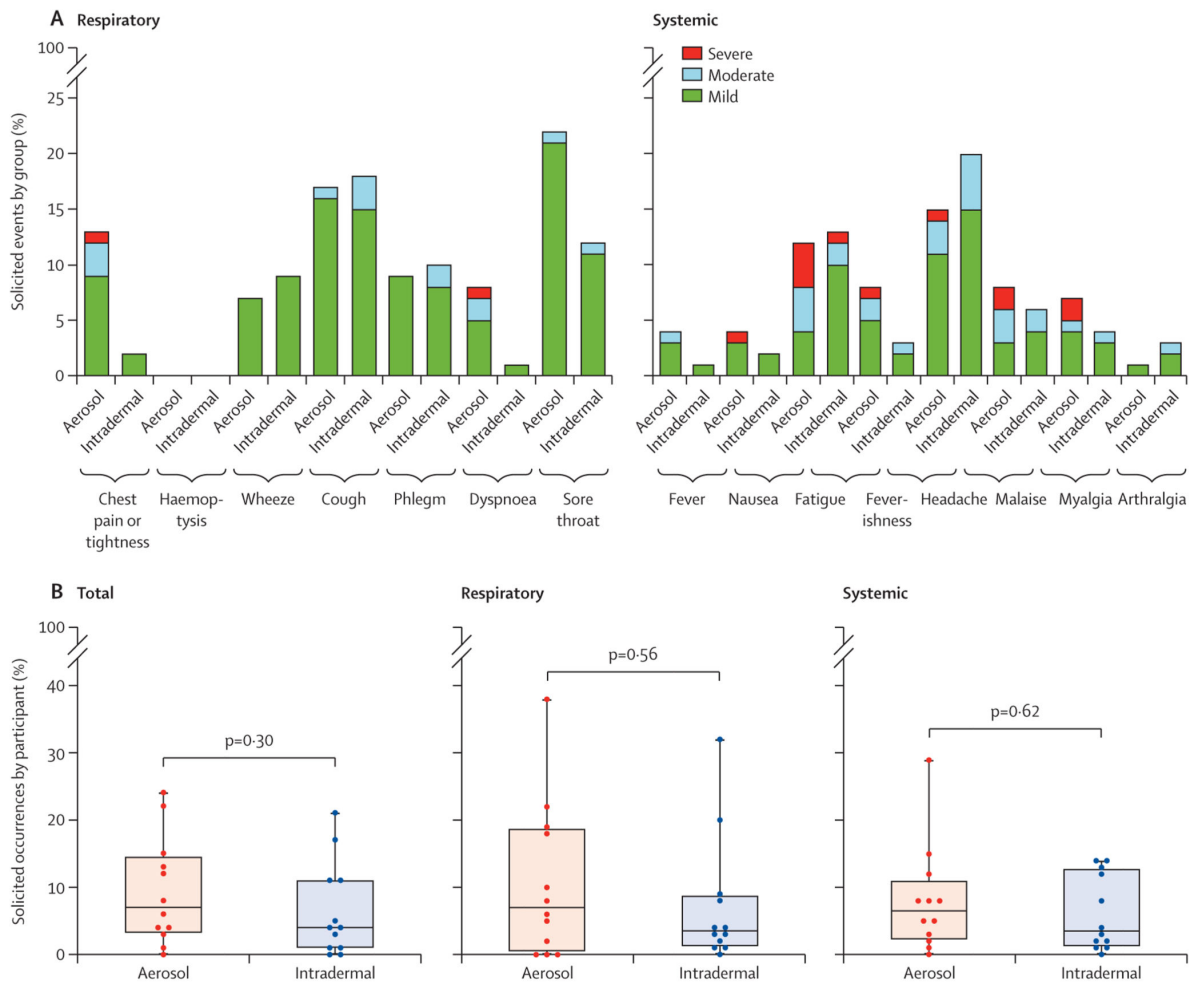
Adverse events in group 2 (BCG Bulgaria) (A) Occurrences of respiratory (left) and systemic (right) adverse events during the 2 weeks after infection with BCG Bulgaria and before bronchoscopy. Shown as a percentage of total solicited occurrences, for the aerosol group (n=12, including n=3 from group 2D and n=9 from group 2E [both 1 × 10^7^ CFU]) and the intradermal group (n=12 from group 2F [1 × 10^6^ CFU]), calculated as 12 participants × 16 timepoints. Adverse events were solicited every 12 h for 2 days after infection and then daily. (B) Total (left), respiratory (middle), and systemic (right) solicited adverse events per participant (as a percentage of the total number of solicited adverse events [15 adverse events × 16 collected timepoints], respiratory adverse events [seven adverse events × 16 collected timepoints], and systemic adverse events [eight adverse events × 16 collected timepoints], respectively) in the 2 weeks after infection with aerosol (n=12) or intradermal (n=12) BCG Bulgaria. Adverse events were collected every 12 h for 2 days following infection and then daily. No significant difference between aerosol and intradermal groups was seen by Mann–Whitney *U* tests. Each dot represents one participant. The middle horizonal lines represent the median, the box boundaries show the IQR, and the outer lines depict the range. CFU=colony-forming units.

Most symptoms by both infection routes in group 2 were mild and all were transient (figure 4A). No treatment was required other than over-the-counter medication for symptomatic relief. No significant difference was observed in the frequency of adverse events between aerosol and intradermal groups (median percentage of solicited adverse events per participant, post-aerosol *vs* post-intradermal BCG: systemic 7% [IQR 2–11] *vs* 4% [1–13], p=0·62; respiratory 7% [1–19] *vs* 4% [1–9], p=0·56). There were also no significant differences in duration or severity of adverse events between infection routes after administration, although there was a non-significant trend of increased nausea, feverishness, and chest pain or tightness in the first 72 h after aerosol BCG (table 2, figure 4B; appendix p 27). In addition, of the participants with systemic symptoms in the first 2 weeks, symptoms were more likely to be reported as severe in those who received aerosol infection (15 [12%] of 122 reported adverse events) than in those who received intradermal infection (one [1%] of 94; difference 11% [95% CI 5–17]; p=0·0013; table 3).

For both routes, the most common respiratory adverse event after BCG infection was cough or sore throat. No difference in spirometry readings was observed between infection routes (appendix p 13), and all electrocardiograms were normal.

One participant in the high-dose aerosol BCG group (group 2E; 1 × 10^7^ CFU) reported fever with 1 week of chest tightness and dyspnoea commencing within 12 h after BCG infection. They had a reduction in transfer factor for carbon monoxide with normal peripheral oxygen saturation (appendix pp 25–26). The participant was assessed at the hospital outpatient respiratory clinic. A putative diagnosis of hypersensitivity pneumonitis was made and the participant was discharged from the clinic without treatment. All symptoms spontaneously resolved. This event was deemed probably related to BCG. A further participant in group 2D had chest tightness without a change in lung function on day 10 after receipt of BCG. This participant had a vasovagal syncope associated with dyspnoea and chest tightness. Paramedics assessed the participant, deemed them well, and did not administer treatment; this event was deemed possibly related to BCG. One participant in group 2A (1 × 10^4^ CFU aerosol BCG) attended the emergency department with chest pain and dyspnoea at day 15 after receipt of BCG, but was discharged without treatment; this event was deemed possibly related to BCG. Peripheral oxygen saturation at clinic visits remained normal for all participants.

Six (50%) of the 12 participants in group 2D/E (aerosol BCG; 1 × 10^7^ CFU) had increased C-reactive protein concentrations at day 2 (range 9·8–90·5 mg/L; normal range <5 mg/L for the Oxford laboratory and <10 mg/L for the Birmingham laboratory), which resolved by day 7–14 in all participants. Two (17%) of the 12 participants in group 2F (intradermal BCG; 1 × 10^6^ CFU) had increased C-reactive protein concentrations at day 14 (23·6 mg/L and 29·0 mg/L). Other laboratory adverse events were too infrequent to compare between groups and are summarised in the appendix (pp 25–26).

In participants who were febrile after BCG infection, no respiratory viral infections were detected on nasopharyngeal swab PCR and all blood cultures were negative. Of those receiving high-dose aerosol BCG, participants who were unmasked to treatment assignment (group 2D) were more likely than those who were masked (group 2E) to report a respiratory adverse event in the 2 weeks after BCG infection (median percentage of solicited respiratory adverse events per participant 22% [IQR 19–38] *vs* 5% [0–9]; p=0·0091, Mann–Whitney *U* test**;** appendix p 14). All bronchoscopies in group 2 showed macroscopically normal results after aerosol infection and no procedural complications were reported for any bronchoscopy. One participant in group 2F had a polypoid mucosal lesion noted macroscopically on bronchoscopy following saline inhalation. After referral for further investigation, this was deemed non-clinically significant on bronchoscopy and biopsy.

After bronchoscopy, an increase in adverse events was observed for both infection routes, consistent with known post-bronchoscopy symptoms; neither adverse event frequency nor severity differed by infection route (table 3, appendix p 38).

**Figure 5:**
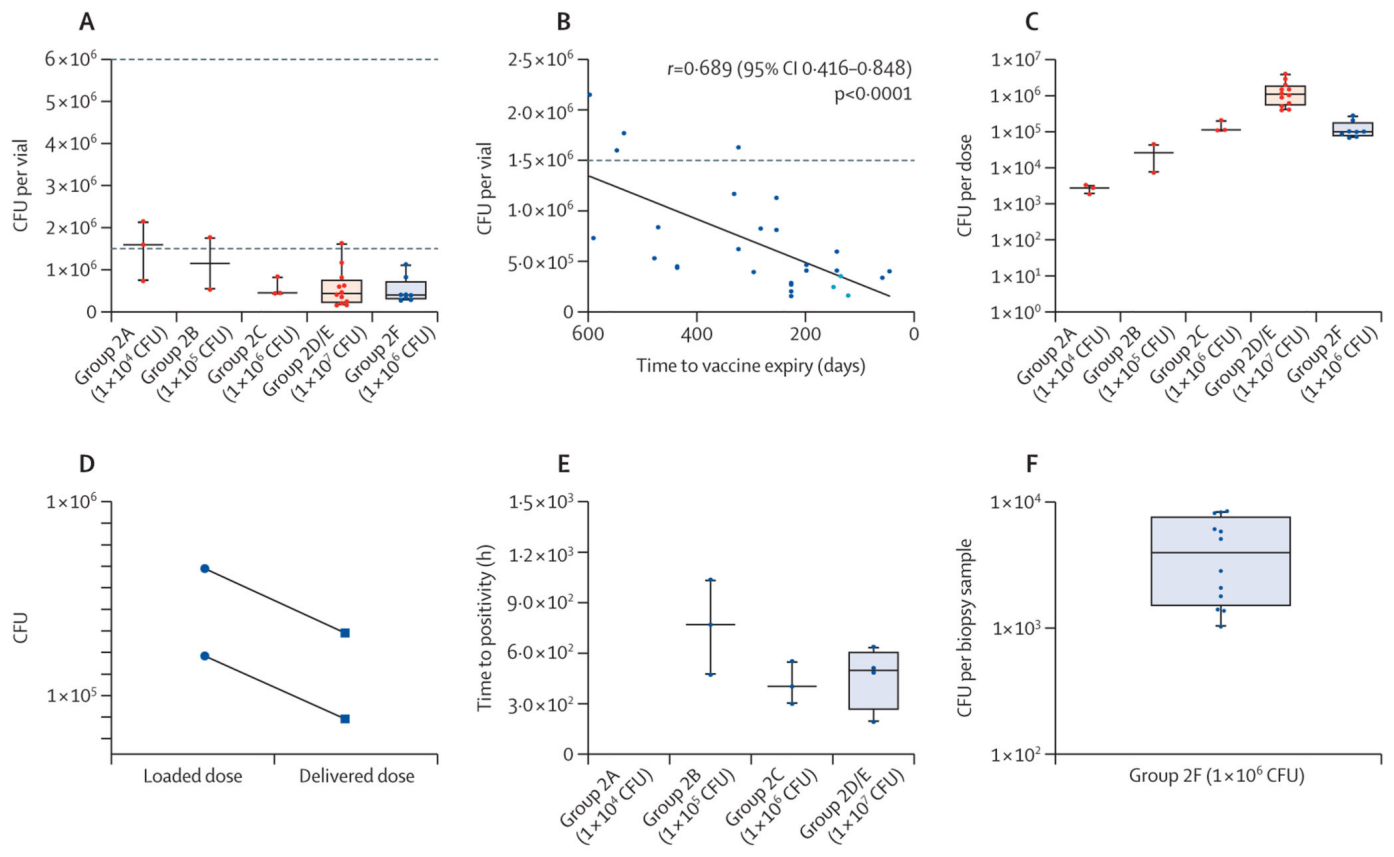
BCG quantification in group 2 (BCG Bulgaria) (A) BCG recovered from the vaccine vials, plated onto Middlebrook 7H11 agar. The range of CFU as stated by the manufacturer is represented by the dotted lines (1·5–6·0 × 10^6^ CFU). Dots represent each reconstituted vial. (B) BCG Bulgaria per vial over an 18-month period. The dotted line represents the lower limit of the range of the stated CFU of BCG Bulgaria per vial (1·5 × 10^6^ CFU). Dots represent each reconstituted vial. Dark blue dots indicate batch 175-2, light blue dots indicate batch 204-1. (C) BCG CFU per dose measured from the diluted or concentrated vials after participant infection, plated onto Middlebrook 7H11 agar. Dots represent each reconstituted vial (prepared dose) (participants infected on the same day could have been infected with BCG from the same prepared dose). (D) Recovery of BCG after nebulisation. After vial reconstitution as per manufacturer instructions, BCG CFU were enumerated before addition to the nebuliser (loaded dose; circles) and after nebulisation (delivered dose; squares) by plating onto Middlebrook 7H11 agar. Two vials were reconstituted and nebulised in a duplicate experiment, in which 46·7% and 47·5% (mean 47·1%) of the BCG was recovered from the original loaded dose; each line represents a separate vial. (E) MGIT culture data from bronchoalveolar lavage samples, presented as time to positivity (h) in participants in the aerosol BCG Bulgaria groups. For dose-escalation groups 2A–C (1 × 10^4^–1 × 10^6^ CFU), the whole bronchoalveolar lavage sample was cultured; for the aerosol comparator groups 2D/E (1 × 10^7^ CFU), bronchoalveolar lavage cells were first removed for immunology and only the fluid was cultured for mycobacterial growth detection. (F) Quantification of BCG in skin biopsy samples taken 14 days after infection in the intradermal group 2F, measured directly by plating the biopsy homogenate onto solid agar. Median CFU recovered 3970 (IQR 1500–7650). Dots represent individual participants. In panels A, C, E and F, the middle horizontal solid lines represent the median; in groups 2A–C, the outer lines show the upper and lower limits of the IQR and range, whereas in groups 2D/E, the box boundaries show the IQR and the outer lines depict the range. CFU=colony-forming units. MGIT=mycobacterial growth indicator tube.

Reconstituted BCG-Bulgaria CFU counts from vaccine vials in group 2 before dilution were 0·3–1·3 log lower than the median range stated in the summary of product characteristics (figure 5A; appendix p 27). The CFU count was proportional to the time to expiry (figure 5B). BCG dose preparation achieved a log-fold increase per dose-escalation group, but concentrations were lower than the targets (figure 5C; appendix p 27). A mean of 47·1% of the loaded dose was recovered after nebulisation (figure 5D).

In the group 2 dose-escalation groups, the total bronchoalveolar lavage sample recovered at 2 weeks after infection was cultured. Two (67%) of three bronchoalveolar lavage samples in group 2A (1 × 10^4^ CFU) and all three (100%) samples in groups 2B (1 × 10^5^ CFU) and 2C (1 × 10^6^ CFU) were positive on the MGIT (table 4). Times-to-positivity were 473 h, 770 h, and 1037 h for group 2B participants and 300 h, 403 h, and 553 h for group 2C participants (figure 5E, appendix p 28). For the maximum tolerated dose group (group 2D/E; 1 × 10^7^ CFU), bronchoalveolar lavage cells were removed for immunology and only bronchoalveolar lavage fluid was cultured. Five (42%) of 12 bronchoalveolar lavage fluid samples were MGIT-positive in this group. Genotyping (appendix pp 3–4) confirmed all samples as BCG, except those from group 2A, in which the organism could not be identified and the samples were considered contaminated. Samples from subsequent groups were decontaminated using a BD BBL MycoPrep kit (BD; Wokingham, UK). The MGIT time-to-positivity for contaminated samples is not included in figure 5E.

Up to three sputum samples in each aerosol group were MGIT-positive, but only samples from day 2 and day 7 in group 2D/E were identified as BCG by genotyping (table 5).

BCG recovery from skin biopsies from the 12 participants who received intradermal BCG (group 2F) showed less variability between participants than that recovered from the bronchoalveolar lavage in those who received aerosol BCG (figure 5F).

As per protocol, immunological analysis was restricted to the 24 participants in the completed comparator groups of group 2 (group 2D–F). Bronchoalveolar lavage volume and cell counts were similar between participants in the aerosol and intradermal groups and were positively correlated in both groups (appendix p 15).

Aerosol BCG induced a higher frequency of mucosal purified protein derivative (PPD)-specific cytokine responses than intradermal BCG (appendix pp 4, 16, 28–29). Polyfunctional CD4^+^ and CD8^+^ T cells were detected following infection by both routes. The frequencies of some T-cell subsets were significantly higher in the aerosol group than in the intradermal group (appendix p 16).

IFNγ production by PPD-stimulated peripheral blood mononuclear cells peaked at day 7 post-infection, remaining significantly above baseline concentrations up to day 84 (aerosol group) and day 168 (intradermal group; appendix pp 4, 17, 29). At day 7, the peak response for the aerosol group was higher than that for the intradermal group (p=0·045; medians: aerosol 546 spot-forming cells per 1 × 10^6^ peripheral blood mononuclear cells [IQR 121–1439]; intradermal 96 [54–143]; appendix p 29).

Frequencies of cytokine-positive PPD-specific CD4^+^ and CD8^+^ T cells and PPD-specific polyfunctional CD4^+^ T cells were measured in whole blood in both groups (appendix pp 6, 18, 30–36). Baseline cytokine responses were higher in the aerosol group than the intradermal group (appendix p 18). There were no major differences in demographic characteristics between participants from the Oxford and Birmingham centres, and day 0 responses in the whole-blood intracellular cytokine staining assay did not differ significantly between participants at the two sites (appendix pp 6, 19).

Baseline serum PPD-specific IgG and IgA concentrations were similar in the aerosol and intradermal groups. BCG infection induced significant systemic PPD-specific IgG and IgA responses via both infection routes (appendix pp 6, 20, 36–37). PPD-specific bronchoalveolar lavage antibody responses did not differ between the aerosol and intradermal groups (appendix p 20).

## Discussion

To our knowledge, this is the first controlled human infection study to deliver aerosol-inhaled BCG to *M tuberculosis*-naive and BCG-naive participants, mimicking the natural route of *M tuberculosis* infection. This infection route is sufficiently well tolerated. Most adverse events were mild and all were self-limiting. The frequency of adverse events did not differ between infection routes, but participants who received aerosol BCG were more likely than those who received intradermal BCG to report severe systemic adverse events. Systemic symptoms such as fever could indicate disseminated BCG infection; however, blood cultures were negative in all febrile participants and symptoms spontaneously resolved.

Reports of cough and sore throat were surprisingly frequent in participants who received intradermal BCG. Unlike hypertonic saline, which is a known mild airway irritant, the 0·9% saline delivered in this study is physiologically isotonic. Isotonic saline has been reported to alleviate symptoms of bronchiectasis^12^ and could have had a clinical effect in our participants, perhaps explaining some of the reported respiratory adverse events. Although no concurrent viral infection was detected in participants after BCG infection, swabs were collected only from febrile participants or those with a grade 2 adverse event, and upper respiratory tract viral infections in participants infected via aerosol or intradermally cannot be excluded. Transient alveolitis, which can be symptomatic, might be expected after BCG infection and might explain the chest tightness and transient decrease in transfer factor for carbon monoxide in some participants.^13^

The increased frequency of respiratory adverse events in group 2D (unmasked) compared with group 2E (masked) participants could be due to over-reporting of adverse events. These participants knew that they had inhaled BCG and were the first participants to inhale a higher dose. This finding highlights the importance of masking in trial design.

In the study by Davids and colleagues,^7^ in which BCG was instilled through a bronchoscope in BCG-vaccinated individuals who were exposed to *M tuberculosis*, 70% of participants reported adverse events, all of which were attributed to bronchoscopy, with no adverse events reported as being definitely related to BCG.^7^ The adverse events in the study by Davids and colleagues were similar to those reported after aerosol BCG inhalation and before bronchoscopy by the participants in our study, and included dyspnoea, chest pain, cough, and fever. In a previous study in which BCG was nebulised into a sealed room (up to 1·9 × 10^5^ CFU), no fever, x-ray changes, or what were termed abnormal complaints were reported.^14^ Adverse events that were reported after the administration of high-dose aerosol BCG in patients with lung cancer are difficult to interpret.^15,16^

We show that BCG can be cultured from bronchoalveolar lavage samples 2 weeks after aerosol BCG infection. No BCG-Danish was grown from samples from the group 1 dose-escalation groups, probably because of the small sample volume cultured in those groups. A 100% recovery rate of live BCG was reached when using whole bronchoalveolar lavage samples (cells and fluid; groups 2B and 2C); this rate decreased to 42% when only bronchoalveolar lavage fluid was cultured (comparator group 2D/E).

An advantage of the BCG skin infection model is that skin biopsies are sterile, unlike bronchoalveolar lavage samples. A positive mycobacterial culture from a bronchoalveolar lavage sample is not necessarily BCG. We genotyped the bronchoalveolar lavage and sputum MGIT culture for species confirmation. We showed that quantifying BCG in the airways following an aerosol BCG infection was possible. Time-to-positivity is a well established method of quantifying mycobacteria, and is more rapid and sensitive (with a detection threshold of fewer than ten organisms) than calculating CFU from solid agar.^17^ No BCG was cultured in sputum from 28 days after aerosol infection. Induced sputum might be less sensitive than bronchoalveolar lavage samples for the detection of subclinical infection, although a 2020 review^18^ concluded that induced sputum was as sensitive as bronchoscopy for the diagnosis of pulmonary tuberculosis in smear-negative patients. Although the lack of cultured BCG is not definitive proof of clearance as BCG could have tracked to the lymph nodes or other tissues, clearance is the most likely outcome because participants had no ongoing symptoms and all had robust immune responses.

When quantifying BCG from vaccine vials, CFU counts were consistently 0·3–1·3 log lower than the median range stated in the summary of product characteristics. Loss of viable bacteria could have occurred between reconstitution and plating, but this loss was likely to be minimal as vials were kept as per the manufacturers’ instructions (in the dark at 2–8°C) and plated within 2 h (6-h reconstituted product viability). Despite all vials being used before the expiration date, the amount of viable BCG Bulgaria was proportional to time to expiry.

Losses of at least 50% occurred after nebulisation. The length of a mycobacterial cell rod is 1·5–4 μm,^19^ and the mesh diameter of the Omron nebuliser is 4·2 μm. Loss during nebulisation could arise from bacterial clumping and shearing of longer bacterial rods. The Omron nebuliser aerosolises to dryness, independent of user effort, and BCG aerosolised during participant exhalation is not inhaled. We conservatively estimated an additional loss of 50% during nebulisation. Therefore, from a dose loaded with 1 × 10^7^ CFU, a median of 5 × 10^5^ CFU of BCG was probably inhaled. Allowing for the 0·5–1 log lower quantity of viable BCG recovered from the vial, participants receiving intradermal BCG also probably received a dose of approximately 5 × 10^5^ CFU. An aerosol BCG study in non-human primates, using the same Omron nebuliser, estimated a similar aerosol-delivered dose loss (1·08 log).^6^ The Omron nebuliser was designed to deposit particles into the lower airways, with an estimated deposition of greater than 70% of the inhaled dose.^20^ The effect of infection via the upper airway versus the lower lung on adverse events and immune responses is unknown.^20^

The median PPD-specific IFNγ response on an enzyme-linked immunosorbent spot assay after intradermal BCG infection was similar to that seen in previous studies, with a broad peak from day 7 to day 14.^4,5^ The response was stronger after aerosol BCG infection than after intradermal infection at the day 7 peak, before decreasing to similar levels to the intradermal group at later timepoints. This pattern is similar to that seen after aerosol delivery of the candidate tuberculosis vaccine MVA85A.^11^ PPD-specific responses with this assay were more durable in the intradermal group.

Baseline whole-blood intracellular cytokine responses differed significantly between the two groups; therefore, between-group comparisons of these responses was not possible. Why baseline responses differed between the groups is unclear, as participants were randomly assigned and enrolled into each group simultaneously. Furthermore, samples were processed in batches at the end of the study and each batch included samples from both study groups, ruling out a batch effect.

Mucosal Th1, Th17, and polyfunctional T-cell responses were higher after aerosol than after intradermal BCG, consistent with previously published data;^7^ these cytokines were selected as they are known to be important in protective immunity against mycobacteria.^21–23^ Further work is needed to evaluate other cytokines, including Th2 cytokines. The role of polyfunctional T cells in the control of human tuberculosis is controversial.^24,25^ Although potent mucosal responses were detected in the aerosol BCG group, there was no difference in bronchoalveolar lavage cell count between the aerosol and intradermal comparator groups. Davids and colleagues^7^ reported that, 3 days after BCG instillation, increased cell counts were detected in the lungs of previously vaccinated South African patients with a history of tuberculosis; by contrast, our participants were naive to both *M tuberculosis* and BCG, had BCG delivered by inhalation, and bronchoscopy was conducted at a later timepoint.^7^

Intradermal BCG induced a significant increase in systemic PPD-specific IgG and IgA concentrations, whereas aerosol-inhaled BCG induced IgA and transient IgG responses. Mycobacteria-specific IgG and IgA concentrations increase after natural *M tuberculosis* infection and could discriminate between patients with active tuberculosis and healthy controls. Following treatment, concentrations of these antibodies decline, which can be attributed to a reduction in bacillary load.^26,27^

This study has some limitations. BCG might not mimic all *M tuberculosis*-induced immune responses; it is not possible to use a BCG controlled human infection model as a model of latent *M tuberculosis* infection; and such a model could not be used to evaluate vaccines containing *M tuberculosis* RD-1 antigens such as ESAT-6 or CFP-10, as this region is deleted from BCG. To limit participant numbers for this initial exploratory study, and because the target dose was not known until dose escalation was complete, only nine of the 12 participants who received the target dose were masked and randomly assigned. Although acknowledging that the numbers are small, unmasked participants might have over-reported adverse events. Participant masking would not have affected BCG recovery or immunology outcome measures.

The sensitivity of BCG detection was higher when the whole bronchoalveolar lavage sample (ie, cells and fluid) was used. BCG recovery could have been compromised by using some cells for immunological analysis. Sensitive quantification of BCG in bronchoalveolar lavage samples is necessary for a controlled human infection model evaluating vaccine efficacy, and in such a model, whole recovered samples—including cells—should be used for culture. Bronchoalveolar lavage washes might not consistently access the same parts of the lungs in different participants, which could affect BCG quantification and immunological analysis.

In conclusion, this aerosol BCG controlled human infection model could facilitate the development of tuberculosis vaccines and enable the identification of potential correlates of protection.

## Supplementary Material

Supplementary appendix

## Figures and Tables

**Table 1:** Baseline characteristics

	Group 1, BCG Danish				Group 2, BCG Bulgaria		
	Aerosol BCG, dose escalation (n=6)	Aerosol BCG (n=4)*	Intradermal BCG (n=3)		Aerosol BCG, dose escalation (n=9)	Aerosol BCG (n=12)†	Intradermal BCG (n=12)
Sex							
Male	4 (67%)	2 (50%)	1 (33%)		2 (22%)	4 (33%)	3 (25%)
Female	2 (33%)	2 (50%)	2 (67%)		7 (78%)	8 (67%)	9 (75%)
Age, years	27 (21–33)	24 (23–29)	25 (20–34)		31 (24–45)	26 (20–32)	24 (21–29)
BMI, kg/m^2^	22 (21–29)	23 (22–25)	23 (21–28)		25 (23–25)	25 (22–30)	26 (22–36)
Spirometry measurements							
Predicted FEV1, %	94% (88–101)	97% (90–103)	91% (86–102)		94% (89–101)	95% (89–104)	97% (87–108)
Predicted FVC, %	92% (91–100)	95% (90–108)	91% (84–99)		99% (92–105)	95% (86–101)	102% (89–112)
FEV1/FVC, %	85% (82–88)	85% (83–87)	87% (84–88)		82% (74–92)	87% (82–89)	84% (79–86)
Baseline SaO_2_, %	97% (97–98)	97% (97–97)	98% (98–99)		99% (97–99)	98% (98–99)	98% (97–99)
Recruitment site							
Birmingham	0	0	1 (33%)		0	6 (50%)	3 (25%)
Oxford	6 (100%)	4 (100%)	2 (67%)		9 (100%)	6 (50%)	9 (75%)
Place of birth							
UK	5 (83%)	3 (75%)	2 (67%)		7 (78%)	10 (83%)	10 (83%)
Canada	··	1 (25%)	··		1 (11%)	··	··
USA	··	··	1 (33%)		1 (11%)	··	··
Australia	··	··	··		··	1 (8%)	··
Ireland	··	··	··		··	1 (8%)	··
Italy	1 (17%)	··	··		··	··	··
New Zealand	··	··	··		··	··	1 (8%)
Poland	··	··	··		··	··	1 (8%)

Data are median (IQR) or n (%). Percentages might not total 100 owing to rounding. FEV1=forced expiratory volume in 1 s. FVC=forced vital capacity. SaO_2_=oxygen saturation of arterial blood. *One participant in this group was enrolled as part of the dose-escalation trial but was included in the comparison between aerosol and intradermal BCG. †Three participants in this group were enrolled as part of the dose-escalation trial but were included in the comparison between aerosol and intradermal BCG.

**Table 2:** Adverse events within 72 h after BCG Bulgaria infection by duration and severity

	Number of participants reporting the adverse event (%), maximum severity			Median adverse event severity (IQR)				Median duration of adverse event (IQR), days		
	Aerosol BCG* (n=12)	Intradermal BCG† (n=12)		Aerosol BCG* (n=12)	Intradermal BCG† (n=12)	p value		Aerosol BCG* (n=12)	Intradermal BCG† (n=12)	p value
**Respiratory**										
Cough	5 (42%), 2	7 (58%), 2		0·0 (0·0–1·0)	1·0 (0·0–1·0)	0·61		1·0 (0·8–12·8)	1·0 (0·5–6·0)	0·56
Sore throat	5 (42%), 1	4 (33%), 2		0·0 (0·0–1·0)	0·0 (0·0–1·0)	>0·99		1·0 (0·5–5·8)	0·5 (0·5–6·9)	0·60
Wheeze	2 (17%), 1	1 (8%), 1		0·0 (0·0–0·0)	0·0 (0·0–0·0)	>0·99		4·5 (1·0–8·0)	16·0	··‡
Dyspnoea	2 (17%), 2	1 (8%), 1		0·0 (0·0–0·0)	0·0 (0·0–0·0)	0·74		2·8 (0·5–5·0)	2·0	·· ‡
Phlegm	2 (17%), 1	1 (8%), 2		0·0 (0·0–0·0)	0·0 (0·0–0·0)	>0·99		1·3 (0·5–2·0)	22·0	·· ‡
Chest pain or tightness	4 (33%), 2	0		0·0 (0·0–1·0)	0·0 (0·0–0·0)	0·093		2·3 (0·8–6·8)	··	·· ‡
**Systemic**										
Fever	3 (25%), 2	0		0·0 (0·0–0·8)	0·0 (0·0–0·0)	0·22		0·5 (0·5–1·5)	··	·· ‡
Arthralgia	2 (17%), 1	1 (8%), 1		0·0 (0·0–0·0)	0·0 (0·0–0·0)	>0·99		0·5 (0·5–0·5)	0·5	·· ‡
Myalgia	5 (42%), 3	2 (17%), 1		0·0 (0·0–1·0)	0·0 (0·0–0·0)	0·21		0·5 (0·5 –2·3)	0·5	·· ‡
Feverishness	7 (58%), 3	2 (17%), 2		1·0 (0·0–1·8)	0·0 (0·0–0·0)	0·065		1·0 (0·6–1·5)	0·5	·· ‡
Headache	9 (75%), 3	7 (58%), 2		1·0 (0·3–1·0)	1·0 (0·0–1·0)	0·40		1·5 (1·0–2·3)	1·0 (0·5–2·5)	0·45
Fatigue	10 (83%), 3	7 (58%), 3		1·0 (1·0–1·8)	1·0 (0·0–1·0)	0·20		1·3 (0·6–2·6)	1·5 (0·5–2·3)	0·94
Nausea	4 (33%), 3	0		0·0 (0·0–1·0)	0·0 (0·0–0·0)	0·093		0·5 (0·5–1·5	··	·· ‡
Malaise	5 (42%), 3	2 (17%), 1		0·0 (0·0–1·8)	0·0 (0·0–1·0)	0·16		1·0 (0·7–2·3)	2·0 (1·0–3·0)	·· ‡

We report any adverse events that occurred within 72 h after BCG infection, ranging in severity from grade 1 (mild) to grade 3 (severe; appendix pp 71–72). Haemoptysis was not recorded in any participant during this timeframe. A Mann–Whitney *U* test was used to compare the median frequency and severity or median duration of adverse events between the aerosol and intradermal groups. Exact p values were calculated using tied ranks. CFU=colony-forming units. *Group 2D/E, receiving 1 × 10^7^ CFU aerosol BCG Bulgaria. †Group 2F, receiving 1 × 10^6^ CFU intradermal BCG Bulgaria. ‡p value not calculated because of insufficient numbers.

**Table 3:** Incidence of severe adverse events compared with mild or moderate events in participants reporting symptoms during the 2 weeks before and after bronchoscopy

	Number of reported severe adverse events as a proportion of total adverse events, n/N (%)		p value	Difference in percentage (95% CI)
	Aerosol BCG* (n=12)	Intradermal BCGt (n=12)		
**First 2 weeks after infection**				
Total (respiratory and systemic)	17/266 (6%)	1/191 (1%)	0·0010	6 (3 to 9)
Respiratory only	2/144 (1%)	0/97	0·52	1 (−1 to 3)
Systemic only	15/122 (12%)	1/94 (1%)	0·0013	11 (5 to 17)
**2 weeks after bronchoscopy (weeks 3 and 4 after infection)**				
Total (respiratory and systemic)	0/109	0/170	··	··
Respiratory only	0/80	0/103	··	··
Systemic only	0/29	0/67	··	··

CFU=colony-forming units. *Participants received 1 × 10^7^ CFU aerosol BCG Bulgaria. †Participants received 1 × 10^6^ CFU intradermal BCG Bulgaria.

**Table 4:** Detection of BCG in bronchoalveolar lavage samples from participants who received aerosol BCG Bulgaria at 2 weeks post-infection

	BCG dose (CFU)	Number of culture-positive samples (%)	Number of culture-positive samples confirmed as BCG by genotyping (%)
Group 2A (n=3)	1 × 10^4^	2 (67%)	0*
Group 2B (n=3)	1 × 10^5^	3 (100%)	3 (100%)
Group 2C (n=3)	1 × 10^6^	3 (100%)	3 (100%)
Group 2D/E (n=12)	1 × 10^7^	5 (42%)	5 (100%)

For BCG detection, the complete bronchoalveolar lavage sample (cells and fluid) was used for participants from dose-escalation groups (2A–C), whereas only the fluid was used for the comparator group 2D/E. The Mycobacteria Growth Indicator Tube system was used for culture and the HAIN assay was used for genotyping. CFU=colony-forming units. *The genotype was unidentified for both samples.

**Table 5:** Detection of BCG in induced sputum from participants who received aerosol BCG Bulgaria

	BCG dose (CFU)	Day 2	Day 7	Day 28	Day 84	Day 168
Group 2A	1 × 10^4^	NA	NA	1/3 (0)	1/3 (0)	1/3 (0)
Group 2B	1 × 10^5^	NA	NA	2/3 (0)	2/3 (0)	1/3 (0)
Group 2C	1 × 10^6^	NA	NA	1/2 (0)	1/3 (0)	1/3 (0)
Group 2D/E	1 × 10^7^	2/3 (2)	3/3 (2)	0/2 (0)	1/3 (0)	1/6 (0)

The numerator shows how many samples were culture-positive (Mycobacteria Growth Indicator Tube system); the denominator is the number of participants from whom samples were obtained at that timepoint. The numbers in brackets show how many culture-positive samples were confirmed as BCG on genotyping (HAIN assay). CFU=colony-forming units. NA=not applicable.

## Data Availability

Any request for raw or analysed data will be reviewed by the study team, and a response can be expected within 14 days. The data generated in this study are subject to patient confidentiality, and the transfer of data or materials will require approval from the sponsor. Any shared data will be de-identified. Requests should be made to HMc.

## Research in context

### Evidence before this study

Development of tuberculosis vaccines is difficult because there are no validated immune correlates of protection and the predictive value of the preclinical animal models is unclear. A controlled human infection model would reduce the financial risks of vaccine development by demonstrating a signal of efficacy in humans before committing to large, expensive efficacy trials. We previously established an intradermal BCG controlled human infection model in which BCG was quantified in skin biopsies collected 2 weeks after BCG infection. These studies—conducted in the UK, where BCG has been shown to protect against tuberculosis—demonstrated that previous BCG vaccination reduces the quantity of BCG recovered from a subsequent intradermal BCG infection. We showed that the magnitude of the immune response correlates inversely with mycobacterial growth in vivo and in vitro and have used this intradermal BCG controlled human infection model to identify immune mechanisms of protection. Although we have demonstrated a BCG vaccine effect with this skin challenge model, an aerosol challenge model would better mimic the natural route of infection of *Mycobacterium tuberculosis*. We searched PubMed from database inception to Dec 8, 2023, with no language restrictions, for published trials of aerosolised BCG in humans using the following terms and combination: “Human” “Lung” “Challenge Model” “bcg” or “aerosol bcg” “mucosal”. The search returned five papers, one of which involved human participants. The study, in BCG-vaccinated participants in South Africa who were exposed to *M tuberculosis*, showed that BCG instillation into lung segments was well tolerated and induced localised cellular, humoral, and transcriptomic responses.

### Added value of this study

To our knowledge, this study is the first in which BCG is delivered as an infectious challenge via the aerosolised route in healthy *M tuberculosis*-naive and BCG-naive adults. Our controlled human infection model mimics the natural route of *M tuberculosis* infection and better models the mucosal response to infection than an intradermal challenge. We show that aerosol BCG induces potent mucosal and systemic cellular immune responses. Aerosol BCG was reasonably well tolerated and there were no related serious adverse events.

### Implications of all the available evidence

This controlled human infection model based on aerosolised BCG could facilitate the development of vaccines (by allowing a signal of efficacy in humans to be detected before committing to large, expensive field efficacy studies), the identification of potential immune correlates of protection, and the evaluation of the immunobiology of mycobacterial infection in humans. Furthermore, given that some animal studies have shown improved protective efficacy when BCG is administered via an aerosol rather than an intradermal route, this study is relevant to the evaluation of the mucosal response to aerosol tuberculosis vaccines.
