# Supplementary material for: Safety of a controlled human infection model of tuberculosis with aerosolised, live-attenuated Mycobacterium bovis BCG versus intradermal BCG in BCG-naive adults in the UK: a dose-escalation, randomised, controlled, phase 1 trial
Source: Lancet Infect Dis. Author manuscript; Available in PMC 2025 Sep 19. (PMC7618001; doi:10.1016/S1473-3099(24)00143-9)
Supplement: Supplementary appendix [file EMS207810-supplement-Supplementary_appendix.pdf]

THE LANCET
Infectious Diseases

Supplementary appendix

This appendix formed part of the original submission and has been peer reviewed. We post it as supplied by the authors.

Supplement to: Satti I, Marshall JL, Harris SA, et al. Safety of a controlled human infection model of tuberculosis with aerosolised, live-attenuated *Mycobacterium bovis* BCG versus intradermal BCG in BCG-naive adults in the UK: a dose-escalation, randomised, controlled, phase 1 trial. *Lancet Infect Dis* 2024; published online April 12. https://doi.org/10.1016/S1473-3099(24)00143-9.

Safety of a controlled human infection model of tuberculosis with aerosolised, live-attenuated *Mycobacterium bovis* BCG versus intradermal BCG in BCG-naive adults in the UK: a dose-escalation, randomised, controlled, phase 1 trial

Iman Satti*, Julia L Marshall*, Stephanie A Harris, Rachel Wittenberg, Rachel Tanner, Raquel Lopez Ramon, Morven Wilkie, Fernando Ramos Lopez, Michael Riste, Daniel Wright, Marco Polo Peralta Alvarez, Nicola Williams, Hazel Morrison, Elena Stylianou, Pedro Folegatti, Daniel Jenkin, Samantha Vermaak, Linnea Rask, Ingrid Cabrera Puig, Rebecca Powell Doherty, Alison Lawrie, Paul Moss, Timothy Hinks, Henry Bettinson, Helen McShane

Supplementary appendix

Table of contents

APPENDIX METHODS 3

BCG DETECTION 3
BCG RECOVERY AFTER NEBULISATION 3
SKIN BIOPSIES 3
QUANTIFICATION OF BCG IN BAL AND INDUCED SPUTUM SAMPLES 3
GENOTYPING OF MYCOBACTERIA ISOLATED FROM BAL AND INDUCED SPUTUM 3
IMMUNOLOGY 4
BRONCHOALVEOLAR LAVAGE (BAL) CELL SEPARATION AND STIMULATION 4
WHOLE BLOOD (WB) STIMULATION 4
INTRACELLULAR CYTOKINE STAINING (ICS) 4
EX-VIVO ENZYME-LINKED IMMUNOSPOT (ELISPOT) ASSAY 4
CONCENTRATION OF BAL FLUID (BALF) 5
ENZYME-LINKED IMMUNOSORBENT ASSAY (ELISA) 5

APPENDIX RESULTS 5

IMMUNOLOGY 5
BRONCHOALVEOLAR LAVAGE (BAL) CELL RECOVERY AND INTRACELLULAR CYTOKINE STAINING 5
EX-VIVO ELISPOT RESPONSES 5
WHOLE BLOOD CYTOKINE RESPONSES 6
SERUM AND BAL PPD-SPECIFIC ANTIBODY RESPONSES 6

REFERENCES 6

APPENDIX FIGURES AND TABLES LEGENDS 8

APPENDIX FIGURES LEGENDS 8

APPENDIX TABLES LEGENDS 9

APPENDIX FIGURES 11

APPENDIX FIGURE 1 11
APPENDIX FIGURE 2 12
APPENDIX FIGURE 3 13
APPENDIX FIGURE 4 14
APPENDIX FIGURE 5 15
APPENDIX FIGURE 6 16
APPENDIX FIGURE 7 17
APPENDIX FIGURE 8 18
APPENDIX FIGURE 9 19
APPENDIX FIGURE 10 20
APPENDIX TABLES 21
APPENDIX TABLE 1 21
APPENDIX TABLE 2 23
APPENDIX TABLE 3 25
APPENDIX TABLE 4 27
APPENDIX TABLE 5 38

CLINICAL TRIAL PROTOCOL 39

Appendix Methods

BCG detection

BCG recovery from vaccine vials, after nebulisation, and from skin biopsies was done by plating serial dilutions onto solid Middlebrook 7H11 agar (BD), as previously published. (1–3). The more sensitive BACTEC MGIT system was used to culture BCG from BAL, as CFU counts are too low to be detectable using traditional plating methods(4–7)

BCG recovery after nebulisation

To quantify BCG lost through nebulisation, a BCG vial was reconstituted with vaccine diluent and further diluted in phosphate-buffered saline (PBS) to give a concentration of $1x10^6$CFU/ml. 1ml was nebulised through the Omron nebuliser, and the aerosol generated collected in a glass impinger. The volume collected was measured and 10ml PBS (minus the volume measured) was used to wash the impinger and serial dilutions plated. A separate aliquot of the starting BCG solution was plated onto Middlebrook 7H11 agar. The experiment was repeated twice.

Skin biopsies

Immediately following D14 bronchoscopy, skin biopsies were performed on ID BCG participants (Groups 1D and 2F), using a sterile technique with a standard 4-mm punch (Stiefel), as previously described(3). The biopsy was taken from the centre of the BCG infection site, snap frozen and stored in liquid nitrogen for batched processing.

All skin biopsy samples were processed on the same day. Samples were thawed in a 37°C water bath and transferred to a Dispomix tube (Miltenyl Biotech) containing 1ml sterile PBS. Tubes were loaded onto a Dispomix machine (Thistle Scientific) and homogenised. 100µL of neat homogenate and 100µL of a 10^{-1} and 10^{-2} dilution were plated in triplicate onto Middlebrook 7H11 agar and incubated at 37°C for 3 weeks. A BCG Bulgaria vaccine vial was reconstituted in PBS and 100µL of a 10^{-2}, 10^{-3}, and 10^{-4} dilution was plated in triplicate as positive controls. The remaining biopsy homogenate was stored at −80°C for later DNA extraction.

BCG DNA was released from 200µL of thawed homogenate using the tough microorganism lysing kit (Precellys) in a Precellys 24 machine at 6500rpm for 3×30 seconds.

Homogenate was transferred to a separate tube and 50µL PBS used to wash remaining homogenate from the beads. 180µL of ATL buffer and 20µL of proteinase K (Qiagen) were added, vortexed, and incubated at 56°C for 4 hours. From this point, the extractions were carried out as previously described(3).

Quantification of BCG in BAL and induced sputum samples

BCG detection was performed on 10ml of BAL from Arm-1 (BCG Danish) participants and the entire BAL volume from Arm-2 (BCG Bulgaria) dose-escalation participants. For the high-dose comparator groups (aerosol BCG Bulgaria $1x10^7$CFU 2D/E; ID BCG Bulgaria $1x10^6$CFU 2F), BAL cells were separated from the fluid and used for exploratory immunological analyses and only the supernatant (BALF) was used for BCG detection.

Induced sputum samples were obtained from all Oxford Arm-2 aerosol BCG participants (dose-escalation and comparator groups) at D28, D84 and D168 post-aerosol BCG, except for three participants in Group 2E (BCG aerosol comparator group $1x10^7$CFU) who had induced sputum samples at D2, D7 and D168 for exploratory analysis, as outlined in the protocol. The ID Group (2F) and aerosol BCG participants who were recruited from Birmingham (n=6) did not have induced sputum collected, as per protocol, for logistic reasons.

A BACTEC™ MGIT tube (Becton Dickinson, UK) was prepared for each BAL or sputum sample by supplementing the Middlebrook 7H9 with 800µL of BBL MGIT OADC (oleic acid, albumin, dextrose, and catalase) and PANTA (polymyxin B, amphotericin B [AMB], nalidixic acid, trimethoprim [TMP], and azlocillin) mixture (Becton Dickinson, UK), as recommended by the manufacturer. All BAL and sputum samples (apart from Group 2A) were decontaminated using the BBL® MycoPrep™ Specimen Digestion/Decontamination Kit (Becton Dickinson, UK) according to the manufacturer's instructions. Briefly, the ampule in the MycoPrep™ Reagent bottle was broken and the N-acetyl-L-cysteine (NALC) dissolved by gentle shaking to activate the NALC-sodium hydroxide (NALC-NaOH) solution. This was then added to the BAL pellet (after centrifugation at 3000g for 17 min and removing the BALF) or sputum sample in equal amounts and left standing at room temperature for 15 minutes with occasional gentle shaking. PBS was then added up to 50ml and the solution centrifuged for 17 minutes at 3000g. The supernatant was discarded and the pellet resuspended in media from the corresponding supplemented MGIT tube and returned to the tube. Tubes were placed on the BACTEC™ MGIT instrument (Becton Dickinson, UK) and incubated at 37°C until the detection of time-to-positivity (TTP) by fluorescence. TTP is inversely associated with the number of CFU present in the sample.

Genotyping of Mycobacteria isolated from BAL and induced sputum

After a minimum of 42 days on the BACTEC™ MGIT instrument (when the manufacturer's protocol considers the sample negative), the contents of each positive MGIT tube was transferred to a 50ml Falcon tube and

centrifuged at 3000g for 17 minutes. Supernatant was discarded and the pellet resuspended in 200µL PBS and stored at -80°C for batched DNA extraction. DNA extraction was carried out as described previously for the skin biopsies(3).
Genotyping was performed using the HAIN™ GenoType MTBC VER 1.X kit (Bruker) according to the manufacturer's instructions. Briefly, DNA extracted from BAL and induced sputum was amplified using kit-specific primers and PCR conditions. 20µL of the PCR product was chemically denatured and hybridised to the kit-specific DNA•STRIP, which contains specific probes complementary to the amplified PCR products. The bound amplicon is detected by addition of streptavidin-alkaline phosphatase and made visible by a colourimetric reaction. This results in a specific banding pattern on the strip, corresponding to a particular mycobacterium species, determined by comparing with the kit specific insert.

Immunology
Immunology endpoints were only performed on the completed randomised control comparator groups in Arm-2 BCG Bulgaria and not on the comparator groups from Arm-1 BCG Danish due to low numbers, as per protocol.

Bronchoalveolar Lavage (BAL) cell separation and stimulation
BAL sample stimulation and intracellular cytokine staining (ICS) was performed on samples collected from volunteers in the randomised comparator groups (aerosol and ID BCG) in Arm-2.
BAL samples were centrifuged for 10 minutes at 400g. 2x1ml aliquots of BAL fluid (BALF) were cryopreserved and the remaining BALF was used for BCG quantification using the BACTEC™ MGIT system as described above. The cell pellet was resuspended and counted using a Casy cell Counter (Innovatis AG). BAL cell concentration was adjusted based on lymphocyte count, then 1×10^6 BAL cells were stimulated with PPD (AJ Vaccines, Denmark) (20ug/ml), Staphylococcal Enterotoxin B (SEB; Sigma-Aldrich) (5µg/ml) or left unstimulated as a negative control for the assay. Co-stimulatory antibodies, anti-CD28 and anti-CD49d (BD Biosciences) at 1µg/ml each, were added to all samples. Cells were incubated in 5% CO_2 at 37°C for 2 hours, then 3µg/ml of Brefeldin A (Sigma-Aldrich) was added, and the samples were incubated overnight and stained the following morning.

Whole Blood (WB) stimulation
Whole blood stimulation and intracellular cytokine staining (ICS) was performed using samples collected from volunteers in the randomised comparator groups (aerosol and ID BCG) in Arm-2.
Fresh WB samples collected at D0, D14 and D168 were stimulated with PPD (20ug/ml), SEB (5µg/ml) or left unstimulated in the presence of co-stimulatory antibodies, anti-CD28 and anti-CD49d (BD Biosciences). Stimulated samples were lysed, fixed, and cryopreserved as previously described(8).

Intracellular cytokine staining (ICS)
BAL cells were washed and stained for viability using Live/dead Red (Thermofisher), followed by surface staining with: CD4-Pacific Blue (BioLegend), CD14 and CD19 on ECD (Beckman Coulter). The samples were then permeabilised and stained intracellularly with CD3-AF700 (eBioscience), CD8-APC/H7 (BD Biosciences), IFN-γ-PE/CY7 (eBioscience), TNF-α-AF647 (BioLegend), IL-2-PE (Beckman Coulter), and IL-17-AF488 (BioLegend).
Flow cytometry was performed on whole blood samples that were stimulated, fixed and frozen for batched flow cytometry staining. Frozen, fixed WB cells were thawed, surface and intracellularly stained as described above. Stained cells were fixed with 1% paraformaldehyde for immediate acquisition on a BD Biosciences LSRII flow cytometer. Flow cytometry data were analysed using FlowJo (BD Biosciences) v10.
ICS gating was conducted by using singlet CD3+/CD14-/CD19- T cells to gate on cytokine+ CD4+ and CD8+ T cells (Appendix Figure2). PPD-specific, background subtracted responses are presented. Analysis of the polyfunctional T cell responses was done using Spice and Pestle (http://exon.niaid.nih.gov/spice/).

Ex-vivo Enzyme-Linked ImmunoSpot (ELISpot) assay
Fresh peripheral blood mononuclear cells (PBMC) separated from whole blood (WB) were used to measure *ex-vivo* IFN-γ ELISpot responses as previously described on samples collected at D0, D7, D14, D28, D84 and D168 of the study(9). Briefly, cells were stimulated in triplicate wells at 3×10^5/well with 20ug/ml of PPD (AJ Vaccines, Denmark), SEB (positive control; Sigma-Aldrich) or left unstimulated as a negative control for the assay. Background (unstimulated) subtracted PPD-specific responses are presented as Spot Forming Cells (SFC) per 1×10^6 PBMC.

Concentration of BAL Fluid (BALF)

Frozen BALF was thawed and duplicate 20µL volumes used to determine phospholipid concentration by means of a phospholipid assay kit (Sigma-Aldrich). The manufacturer's instructions were followed and the concentration obtained in µM converted to concentration in µg/ml using the molecular weight of phosphatidyl-choline(10).

The physiological concentration of phospholipid in the lung is estimated to be 1mg/ml, so it would have been desirable to concentrate BALF to this physiological concentration(11). However, as only a small volume of BALF was stored, this would not have left enough volume to perform an ELISA. Therefore, samples were normalised to 0.2mg/ml of phospholipid to ensure sufficient volume for ELISA analysis, whilst still increasing likelihood that antibody concentration will rise above the limit of detection. BAL antibody arbitrary units (AU) were then multiplied by 5 in order to present BAL antibody per 1mg/ml phospholipid (see below). BAL samples were concentrated by adding 3x500µL to separate Amicon® Ultra-0.5 Centrifugal Filter Devices (Millipore) and centrifuging at 14,000g for between 5 and 30 minutes, depending on the extent of concentration required. When the desired volume was achieved, the filter unit was inverted into a new collection tube and centrifuged at 1000g for 2 minutes to transfer the concentrated sample to the tube. Samples were stored at –80 °C for later analysis.

Enzyme-Linked Immunosorbent Assay (ELISA)

ELISAs, measuring PPD-specific IgG and IgA, were performed on serum taken at D0, D7, D14, D28, D84 and D168 post-BCG infection in the randomised comparator aerosol and ID Groups (1x10^7CFU aerosol or 1x10^6CFU ID BCG) in Arm-2 and on concentrated BALF taken at D14. ELISA plates (NUNC 442404) were coated with 50µL/well of 5µg/ml PPD in PBS and incubated overnight at 4°C. Plates were washed and blocked with 100µL/well casein for 1 hour at room temperature. Serum samples were diluted 1 in 50 in casein and standards prepared by serial 1 in 2 dilutions of a reference pool of serum. A positive control corresponding to a 1 in 256 dilution of the reference serum was used. Blocking solution was discarded and 50µL of samples and positive control added to triplicate wells. Standards were added in duplicate, and plates incubated at room temperature for 2 hours. Plates were washed and 50µL of a 1 in 1000 dilution of anti-human goat IgG or IgA (y-chain specific)-alkaline phosphatase antibody (Sigma-Aldrich) was added. Plates were incubated for 1 hour at room temperature. Plates were washed and developed by adding 100µL/well of p-Nitrophenyl Phosphate, Disodium Salt (pNPP) substrate (Sigma-Aldrich). After 5 minutes, plates were read at 405nm using Gen5 software (v2.0.7, BioTek) and read every minute for 20-30 minutes to ensure a read where the standards produced a curve with parameters within the specified range. Plates passed if average OD values for the blank wells were < 0.15, the standard curve was within pre-defined parameters and if the OD of replicate sample values had a coefficient of variation <20**%.** BAL antibody values are presented as arbitrary units based on the standard curve and multiplied by 5, based on the phospholipid physiological concentration of 1mg/ml(10).

Appendix Results

Immunology

As per protocol, data analysis was restricted to the 24 volunteers in the completed comparator groups (2D/E 1x10^7CFU aerosol and 2F 1x10^6CFU ID BCG Bulgaria).

Bronchoalveolar Lavage (BAL) cell recovery and Intracellular Cytokine staining

Inhaled BCG induced potent mucosal Purified Protein Derivative (PPD)-specific cytokine responses (Appendix Figure6 / Appendix Table4(F-J)). The frequencies of PPD-specific IFN-γ+, TNF-α+, IL-2+ and IL-17+ CD4+ T cells and IFN-γ+ CD8+ T cells were significantly higher in the aerosolised BCG group compared to the ID group (Appendix Figure6A/B / Appendix Table4F/G). IL-2+ and IL-17+ CD8+ T cells were not detectable.

Polyfunctional CD4+ T cells were detected following BCG infection by both routes. Frequencies of IFN-γ+TNF-α+IL-2+IL-17+, IFN-γ+TNF-α+IL-17+, IFN-γ+TNF-α+IL-2+, IFN-γ+TNF-α+, TNF-α+IL-17+, TNFα+IL-2+, IFN-γ+IL-17+ and IFN-γ +IL-2+ CD4+ T cells were significantly higher in the aerosolised group compared to the ID group (Appendix Figure6C / Appendix Table4I). IFN-γ+TNF-α+ CD8+ T cells were also higher in the aerosolised BCG group (Appendix Figure6D / Appendix Table4J).

***Ex-vivo* ELISpot responses**

Correction for multiple comparisons was done using Friedman test with Dunn's multiple comparisons test: Friedman test statistically significant p-values = 0·04 and 0·01 for the aerosol and ID groups, respectively. The statistically significant Dunn's multiple comparisons test adjusted p-values were; for Aerosol D0 vs D7 = 0·01, and p=0·00, 0·03 and 0·03 for the ID group D0 vs D7, D14 and D28, respectively. Peak (D7) aerosol vs ID group (p=0·05, Mann Whitney test) (Appendix Figure7 / Appendix Table4K)

Whole blood Cytokine responses

A significant increase above baseline of IFN-γ+ CD4+ T cells was detected in the ID group at D14 and D168, no significant increase was detected in the aerosol group. Significant induction of TNF-α+ and IL-2+ CD4+ T cells was detected following both aerosolised and ID BCG infections at D14, and at D168 in the ID group (Appendix Figure8A / Appendix Table4L). Multiple comparison correction was done using Friedman test with Dunn's multiple comparisons test: For CD4+ T cell response, statistically significant Friedman test p-values = 0·09, 0·011, 0·018 and 0·47 for the aerosol group IFN-γ, TNF-α, IL-2 and IL-17 CD4+ T cells respectively and p=0·00, 0·013, 0·00, and 0·91 for the ID group IFN-γ, TNF-α, IL-2 and IL-17 CD4+ T cells, respectively). The statistically significant Dunn's multiple comparisons test adjusted p-value for the aerosol group were: p=0·04 and p=0·011 for D0 vs D14TNF-α and IL-2, respectively and Dunn's adjusted p-values for the ID group; p=0·04 and p=0·00 for IFN-γ D0 vs D14 and D0 vs D168, respectively, P=0·02 for TNF-α D0 vs D14 and D0 vs D168, and p=0·00 and p=0·04 for IL-2 D0 vs D14 and D0 vs D168, respectively.

BCG infection did not induce significant IL-17+ CD4+ T cells, nor IFN-γ+ or TNF-α+ CD8+ T cells (Appendix Figure8A/B / Appendix Table4L/M). IL-2+ and IL-17+ CD8+ T cells were not detectable.

PPD-specific polyfunctional CD4+ T cells were detected in both study groups (Appendix Figure8C/D / Appendix Table4N/O). The frequencies of IFN-γ+TNF-α+IL-2+IL-17+, IFN-γ+TNF-α+, TNF-α+IL-2+ and IFN-γ+IL-2+, CD4+ T cells were significantly increased at D14 compared to D0 in the aerosol and ID groups, IFN-γ+IL-2+ CD4+ T cells also increased at D168 in the ID group (Appendix Figure8C/D). The frequency of IFN-γ+TNF-α+IL-2+ CD4+ T cells significantly increased at D14 and D168 in the ID BCG group (Appendix Figure8D). Multiple comparison correction analysis for the aerosolised BCG group polyfunctional CD4+ T cells was done using Friedman test with Dunn's multiple comparisons test: Friedman test statistically significant p-values = (0·00, 0·01 and 0·00 for g+t+2+17+, g+t+2-17- and g-t+2+17-, respectively. The statistically significant Dunn's multiple comparisons test adjusted p-value were: Dunn's multiple comparisons test adjusted p-value (D0 vs D14; p=0·00, 0·04 and 0·01 for g+t+2+17+, g+t+2-17- and g-t+2+17-, respectively). ID group polyfunctional CD4+ T cells statistically significant Friedman test: p=0·05, 0·00, 0·00, 0·01, 0·02 and 0·0 for g+t+2+17+, g+t+2+17-, g-t+2+17+, g+t+2-17-, g-t+2+17- and g+t-2+17, respectively). The statistically significant Dunn's multiple comparisons test adjusted p-value (D0 vs D14; p=0·03, 0·01, 0·00, 0·01, 0·01 and 0·04 for g+t+2+17+, g+t+2+17-, g-t+2+17+, g+t+2-17-, g-t+2+17- and g+t-2+17-, respectively and p=0·00 for g+t+2+17- D0 vs D168). The frequencies of polyfunctional CD8+ T cells did not increase after BCG infection by either route (Appendix Figure8E / Appendix Table4P).

Baseline D0 cytokine responses were significantly higher in the aerosol group compared to the ID group (p=0·00, p=0·00, p=0·00 and p=0·01 for IFN-γ, TNF-α, IL-2 and IL-17 CD4+ T cells respectively (Mann-Whitney test for comparing each cytokine response between the two study groups) (Appendix Figure8A / Appendix Table4L). While there was no significant difference in any demographic characteristic analysed between the two groups, 50% of the aerosolised group volunteers were recruited from Birmingham compared to 25% in the ID group. However, WB ICS D0 responses were not significantly different between the Birmingham and Oxford volunteers and all volunteers were not latently infected (Interferon-Gamma Release Assay negative) at enrolment. Unstimulated total cytokine responses were comparable in volunteers in the two study groups (p>0.05 for all CD4+ and CD8+ T cells cytokines, Mann-Whitney test) (Appendix Figure9 / Appendix Table4L/M).

Serum and BAL PPD-specific antibody responses

Significant serum PPD-specific IgG responses were detected at D7 in the aerosol comparator BCG group and up to D84 following ID BCG infection (Appendix Figure10A / Appendix Table4Q). PPD-specific serum IgA remained higher than baseline in both groups up to D168 (Appendix Figure10B / Appendix Table4R). Multiple comparison correction was done using Friedman test with Dunn's multiple comparisons test: Statistically significant Friedman test (p < 0·0001 for IgG in the aerosol and ID groups and p=0·00 and 0·00 for IgA in the aerosol and ID groups, respectively).

The statistically significant Dunn's multiple comparisons test adjusted p-value were: IgG Dunn's multiple comparisons test adjusted p-value = 0·02 (D0 vs D7) for the aerosol group, and for the ID group; p<0·0001, for D0 vs D7 and D0 vs D14 and p=0·01 for D0 vs D28. IgA Dunn's multiple comparisons test adjusted p-values = 0·01 and 0·01 for the aerosol group D0 vs D84 and D0 vs D168. For the ID group, IgA Dunn's adjusted p-value = 0·01, 0·00, 0·01 and 0·00 for D0 vs D7, D0 vs D14, D0 vs D84 and D0 vs D168, respectively.

BAL antibodies were normalised to a phospholipid concentration of 1mg/ml.

References

1. Harris SA, Meyer J, Satti I, Marsay L, Poulton ID, Tanner R, et al. Evaluation of a human BCG challenge model to assess antimycobacterial immunity induced by BCG and a candidate tuberculosis vaccine, MVA85A, alone and in combination. J Infect Dis [Internet]. 2014 Apr 15 [cited 2018 May 28];209(8):1259–68. Available from: https://academic.oup.com/jid/article-

lookup/doi/10.1093/infdis/jit647
2. Minhinnick A, Harris S, Wilkie M, Peter J, Stockdale L, Manjaly-Thomas Z-RR, et al. Optimization of a Human Bacille Calmette-Guérin Challenge Model: A Tool to Evaluate Antimycobacterial Immunity. [cited 2018 May 28];213(5). Available from: https://academic.oup.com/jid/article-lookup/doi/10.1093/infdis/jiv482
3. Minassian AM, Satti I, Poulton ID, Meyer J, Hill AVSS, McShane H. A Human Challenge Model for Mycobacterium tuberculosis Using Mycobacterium bovis Bacille Calmette-Gue´rin. J Infect Dis [Internet]. 2012 Apr 1 [cited 2018 May 28];205(7):1035–42. Available from: https://academic.oup.com/jid/article-lookup/doi/10.1093/infdis/jis012
4. Campelo TA, Sousa PRC de, Nogueira L de L, Frota CC, Antas PRZ. Revisiting the methods for detecting Mycobacterium tuberculosis: what has the new millennium brought thus far? Access Microbiol [Internet]. 2021 Aug 2 [cited 2023 Jun 20];3(8):245. Available from: /pmc/articles/PMC8479963/
5. Sorlozano A, Soria I, Roman J, Huertas P, Soto M, Piedrola G, et al. Comparative evaluation of three culture methods for the isolation of mycobacteria from clinical samples. J Microbiol Biotechnol [Internet]. 2009 [cited 2023 Jun 20];19(10). Available from: https://pubmed.ncbi.nlm.nih.gov/19884789/
6. Diacon AH, Maritz JS, Venter A, Van Helden PD, Dawson R, Donald PR. Time to liquid culture positivity can substitute for colony counting on agar plates in early bactericidal activity studies of antituberculosis agents. Clin Microbiol Infect [Internet]. 2012 Jul 1 [cited 2023 Jun 20];18(7):711–7. Available from: https://onlinelibrary.wiley.com/doi/full/10.1111/j.1469-0691.2011.03626.x
7. de Knegt GJ, Dickinson L, Pertinez H, Evangelopoulos D, McHugh TD, Bakker-Woudenberg IAJM, et al. Assessment of treatment response by colony forming units, time to culture positivity and the molecular bacterial load assay compared in a mouse tuberculosis model. Tuberculosis. 2017 Jul 1;105:113–8.
8. Hanekom WA, Hughes J, Mavinkurve M, Mendillo M, Watkins M, Gamieldien H, et al. Novel application of a whole blood intracellular cytokine detection assay to quantitate specific T-cell frequency in field studies. J Immunol Methods [Internet]. 2004 Aug [cited 2019 Oct 13];291(1–2):185–95. Available from: https://linkinghub.elsevier.com/retrieve/pii/S002217590400225X
9. Meyer J, Harris SA, Satti I, Poulton ID, Poyntz HC, Tanner R, et al. Comparing the safety and immunogenicity of a candidate TB vaccine MVA85A administered by intramuscular and intradermal delivery. Vaccine. 2013 Feb 4;31(7):1026–33.
10. Moliva JI, Rajaram MVS, Sidiki S, Sasindran SJ, Guirado E, Pan XJ, et al. Molecular composition of the alveolar lining fluid in the aging lung. Age (Omaha) [Internet]. 2014 [cited 2022 Jul 28];36(3):1187–99. Available from: /pmc/articles/PMC4082594/
11. Arcos JS, Sasindran SJ, Fujiwara N, Turner J, Schlesinger LS, Torrelles JB. Human Lung Hydrolases Delineate Mycobacterium tuberculosis-Macrophage Interactions and the Capacity To Control Infection. J Immunol [Internet]. 2011 [cited 2023 Mar 27];187:372–81. Available from: www.jimmunol.org/cgi/doi/10.4049/jimmunol.1100823

Appendix Figures and Tables legends
Appendix Figures legends

Appendix Figure 1. Study design

A. Study design for dose-escalation and comparator groups

Blood for exploratory immunology taken at D0, D2, D7, D14, D28, D84 and D168

*The 12 participants for aerosol BCG Bulgaria comparator group ($1x10^7$CFU) were made up of the three 2D unblinded dose-escalation participants and the nine randomised blinded 2E participants.

B. Study design

Study groups Arm-1 BCG Danish SSI and Arm-2 BCG Bulgaria Intervax, target recruitment numbers. Participants in the dose-escalation groups were sequentially enrolled and not blinded (Groups 1A, 1B, 2A-D).

C. Arm-1 Study design

First phase dose-escalation followed by second phase comparator groups. For safety reasons, the first participant in Group 1C was sequentially enrolled and not randomised as this was a dose-escalation, however as with all Group 1C participants, they still received $1x10^5$ CFU aerosol BCG and ID saline. Arrow indicates at least 7 days until the next participant was enrolled. Boxes on left show the actual numbers enrolled, boxes on right show the planned enrolment numbers per group prior to the protocol amendment. Prior to protocol amendment due to global BCG Danish shortage, planned recruitment for Group 1C and 1D was 12 participants per group.

D: Arm-2 study design

First phase dose-escalation followed by second phase 2 comparator groups. Group 2D (unblinded $1x10^7$ CFU aerosol BCG) was both a dose-escalation group and was also included in the aerosol comparator group for analysis. Arrow indicates at least 7 days until the next participant was enrolled.

Appendix Figure 2. Flow Cytometry gating strategy

Whole blood intracellular cytokine staining gating strategy. Lymphocytes and singlet cells (A and B) were used to gate CD3+ T cells (C), then CD4+ and CD8+ T cells (D) were gated off the CD3+ positive population. IL-2, IL-17, IFN-γ and TNF-α were gated on CD4+ T cells (E-G) and CD8+ T cells (H-J).

Appendix Figure 3. participant spirometry following aerosol or intradermal (ID) BCG Bulgaria infection. (A) FEV1 (B) FVC

Median percent change in FEV1 (A) and FVC (B) from participant's baseline reading (D0 pre-infection), in comparator aerosol ($1x10^7$CFU) or intradermal ($1x10^6$CFU) groups in the fourteen days following BCG Bulgaria infection (Arm-2). Taken from participant e-diaries (self-reported). Median and interquartile (IQR) range shown for each group. Note different scale D0-D3 vs D4-D13. FEV1: Forced expiratory volume in 1 second; FVC: Forced vital capacity. Median area under the curve analysis: FEV1 median reduction in aerosol -30·40 (IQR -47·80 to -17·30); intradermal -58.70 (IQR -97·00 to -8·60), p=0·22 FVC median reduction in aerosol -41·80 (IQR -68·10 to -7·20); intradermal -14·80 (IQR -51·90 to -4·90), p=0·64.

Appendix Figure 4. Frequency of solicited respiratory AEs reported between blinded (Group 2E) and unblinded (Group 2D) participants following $1x10^7$CFU aerosol BCG Bulgaria inhalation

Percent of AEs by participant out of total solicited event points in the 2 weeks following BCG Bulgaria infection, by group. AEs symptoms were collected every 12 hours for 2 days following infection then daily. Total solicited occurrences: Seven respiratory adverse events x 16 collected time points; Aerosol BCG Bulgaria $1x10^7$ CFU: Group 2D n=3 unblinded and Group 2E n=9 blinded.

P-value calculated as difference in median by Mann-Whitney. Each dot represents one participant; lines show median response, IQR and range.

Appendix Figure 5. Bronchoalveolar lavage cell count in samples collected from participants in comparator groups aerosol ($1x10^7$CFU) and intradermal ($1x10^6$CFU) BCG

A. BAL samples volume (ml) collected from participants in the comparator aerosol ($1x10^7$CFU) and intradermal ($1x10^6$CFU) study groups. Bars represent median, IQR and range.
B. BAL cell count in samples collected from participants in the two study groups, bars represent median, IQR and range.
C. Spearman correlation between BAL volume (ml) and cell count in samples collected from participants in Groups 2D/2E (aerosolised BCG).
D. Spearman correlation between BAL volume (ml) and cell count in samples collected from participants in Group 2F (intradermal BCG).

Dots represent individual participants. Medians, IQR and range are shown.

Appendix Figure 6. Bronchoalveolar lavage Intracellular Cytokine staining

Bronchoalveolar Lavage Intracellular cytokine response in the comparator groups, aerosol ($1x10^7$CFU) and the intradermal ($1x10^6$CFU) groups. PPD-specific cytokine responses are presented as percentages of CD4+ T cells (A) and CD8+ T cells (B). Figures C and D show polyfunctional CD4+ T cells and IFN-γ+/TNF-α+ CD8+ T cells, respectively. Dots represent individual responses and horizontal lines indicate median responses, IQR and range. P-values using the Mann-Whitney test for comparing each cytokine response between the two study groups in samples collected at day 14 are presented.

Appendix Figure 7. *Ex-vivo* PPD-specific ELISpot responses following BCG Bulgaria infection
PPD-specific ELISpot response in the comparator groups, aerosol ($1x10^7$CFU) and the intradermal ($1x10^6$CFU) groups.
Background subtracted antigen-specific responses are presented in Spot Forming Cells (SFC)/$1x10^6$ PBMC. Statistically significant p-values from Wilcoxon matched-pairs signed rank test are presented in the figure.
Dots represent individual participants with horizontal lines representing median, IQR and range.

Appendix Figure 8. Whole blood Intracellular Cytokine staining
Whole blood intracellular cytokine response in the comparator aerosol ($1x10^7$CFU) and the intradermal ($1x10^6$CFU) groups. PPD-specific cytokine responses are presented as percentages of:

A. CD4+ T cell
B. CD8+ T cell cytokines
C. Polyfunctional CD4+ T cells producing multiple cytokines simultaneously (aerosol group).
D. Polyfunctional CD4+ T cells producing multiple cytokines simultaneously (intradermal group).
E. Polyfunctional CD8+ T cells producing IFN-γ and TNF-α simultaneously.

Wilcoxon matched-pairs signed rank test was used for repeated measures comparisons, statistically significant p-values are presented in the figure.
Dots represent individual participants with horizontal lines representing median, IQR and range.

Appendix Figure 9. Whole blood ICS baseline response by site of participants' recruitment
Dots represent individual participants. Medians, IQR and range are shown. P>0·05 for each of the presented cytokines (Mann-Whitney test).

Appendix Figure 10. Serum and Bronchoalveolar lavage IgG and IgA following BCG Bulgaria infection
PPD-specific serum (A) IgG and (B) IgA responses in participants who received aerosol ($1x10^7$CFU) or intradermal ($1x10^6$CFU) BCG Bulgaria. The dashed lines represent the cut-off value for seropositivity (70 EU for IgG and 27 EU for IgA).
BAL antibodies were normalised to a phospholipid concentration of 1mg/ml (C) IgG and (D) IgA.
Wilcoxon matched-pairs signed rank test was used for repeated measures comparisons, statistically significant p-values are presented in the figures.
Individual dots represent PPD-specific antibody titres per participant. Medians, IQR and range are shown. EU: Elisa units.

Appendix Tables Legends
Appendix Table 1. Inclusion and exclusion criteria
List of inclusion and exclusion criteria

Appendix Table 2: Protocol deviations
List of study protocol deviations

Appendix Table 3. Unsolicited adverse events
A. Arm-1: Adverse events that were deemed probably or definitely related to either the aerosol BCG challenge or any other study procedure are presented here. Intradermal (ID); Colony Forming Units (CFU). Normal reference ranges: White Cell Count (WCC): 4.00 - $11.00x10^9$/L; C-Reactive Protein (CRP): <5mg/L.
B. Arm-2: Adverse events that were deemed probably or definitely related to either the aerosol BCG challenge or any other study procedure are presented here if not already included in the body of the manuscript. Intradermal (ID); Colony Forming Units (CFU). Normal reference ranges: Haemoglobin (Hb) 120-150g/L; White Cell Count (WCC): 4·00 - $11·00x10^9$/L; C-Reactive Protein (CRP): <5mg/L.

Appendix Table 4. Percentiles, medians and interquartile ranges

Appendix Table 5. Solicited Adverse Events (AEs) in the two weeks following bronchoscopy

BCG Bulgaria comparator groups. Aerosol: Group 2D/2E BCG Bulgaria 1x10^7CFU aerosol; ID: Group 2F BCG Bulgaria 1x10^6CFU intradermal.

Appendix Figures

Appendix Figure 1

A

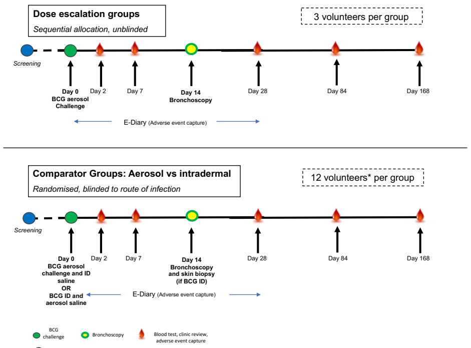

B

Arm 1		
**Group**	**Sample size**	**Intervention**
Group 1A	3	Aerosol inhaled BCG Danish (SSI) 1 x 10^3 CFU then bronchoscopy
Group 1B	3	Aerosol inhaled BCG Danish (SSI) 1 x 10^4 CFU then bronchoscopy
Group 1C	4	Aerosol inhaled BCG Danish (SSI) 1 x 10^5 CFU and intradermal saline placebo then bronchoscopy
Group 1D	3	Aerosol inhaled saline placebo and intradermal BCG Danish (SSI) 1 x 10^5 cfu then bronchoscopy and punch biopsy
**Arm 2**		
**Group**	**Sample size**	**Intervention**
Group 2A	3	Aerosol inhaled BCG Bulgaria (InterVax) 1 x 10^4 CFU then bronchoscopy
Group 2B	3	Aerosol inhaled BCG Bulgaria (InterVax) 1 x 10^5 CFU then bronchoscopy
Group 2C	3	Aerosol inhaled BCG Bulgaria (InterVax) 1 x 10^6 CFU then bronchoscopy
Group 2D	3	Aerosol inhaled BCG Bulgaria (InterVax) 1 x 10^7 CFU then bronchoscopy
Group 2E	9	Aerosol inhaled BCG Bulgaria (InterVax) 1 x 10^7 CFU and intradermal saline placebo then bronchoscopy.
Group 2F	12	Aerosol inhaled saline placebo and intradermal BCG Bulgaria (InterVax), at a dose a log lower than 2E ie 1 x 10^6 CFU, then bronchoscopy and punch biopsy

C

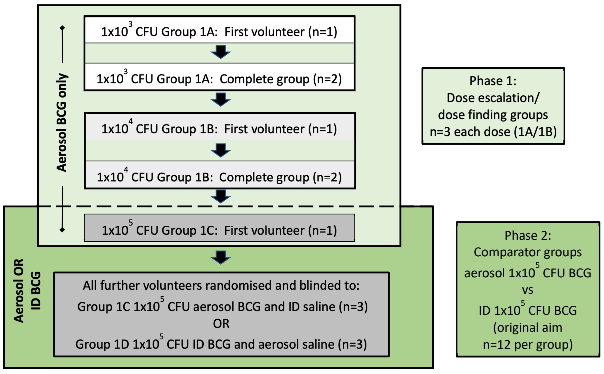

D

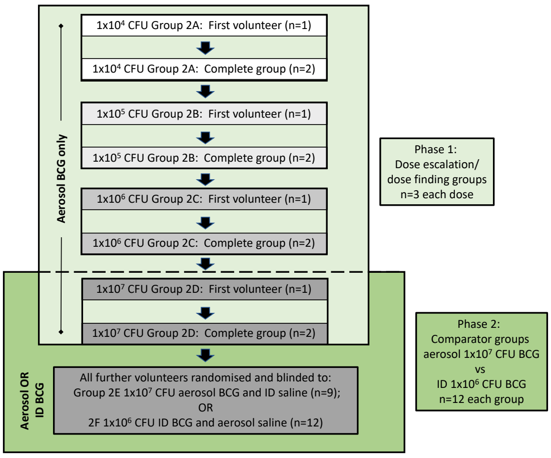

Appendix Figure 2

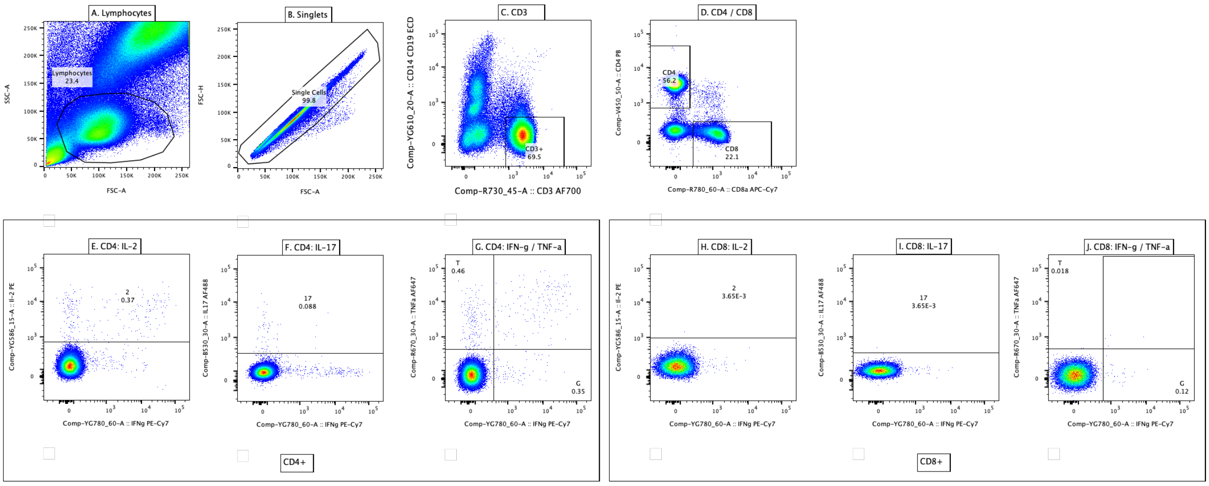

Appendix Figure 3

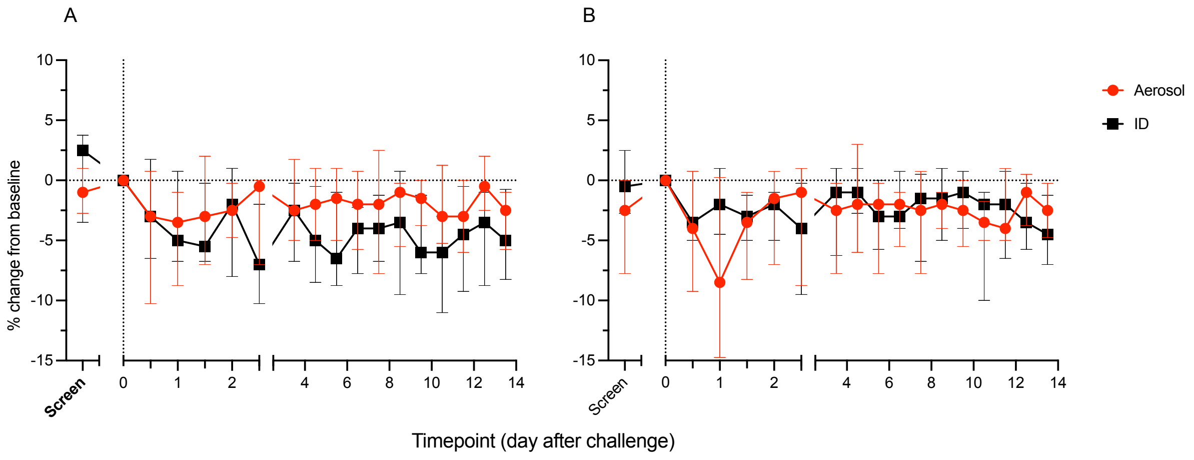

Appendix Figure 4

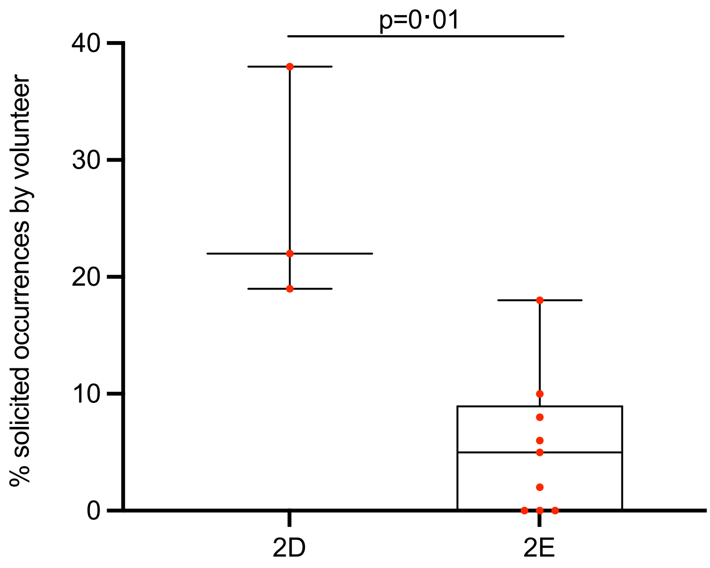

Appendix Figure 5

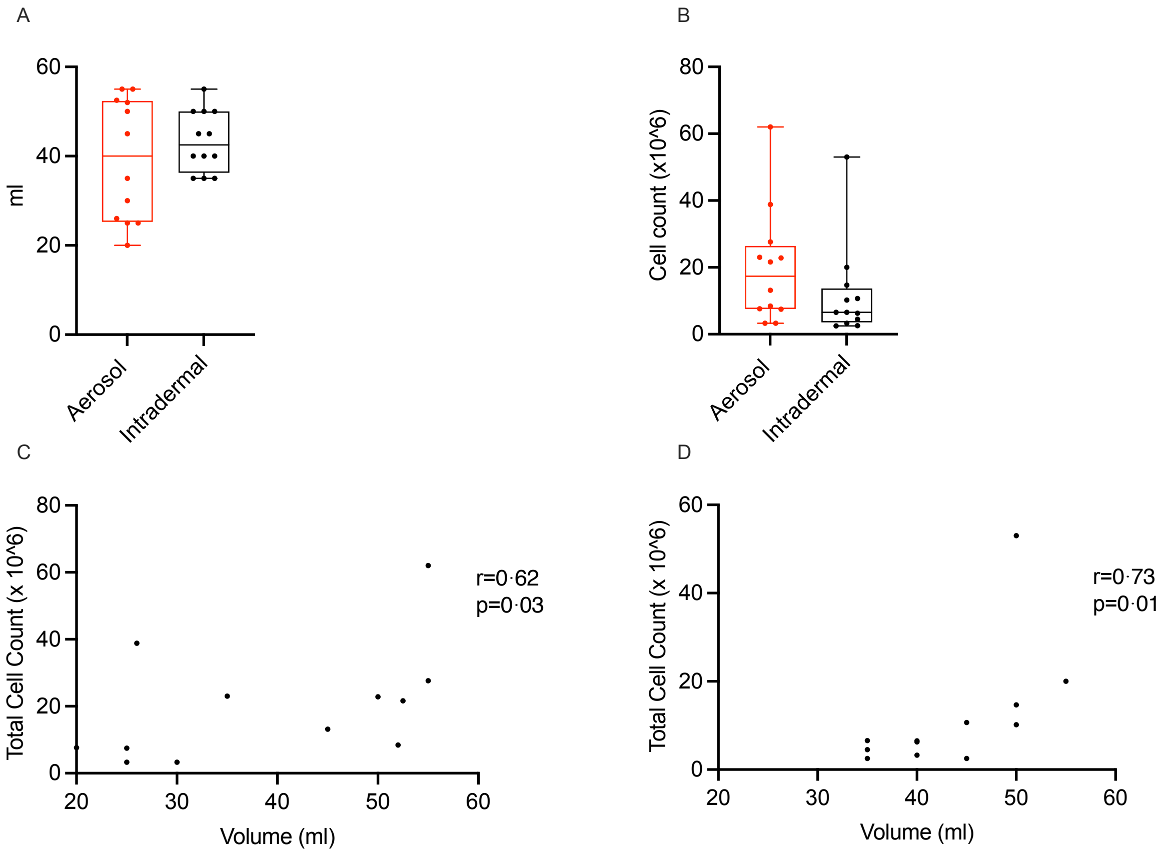

Appendix Figure 6

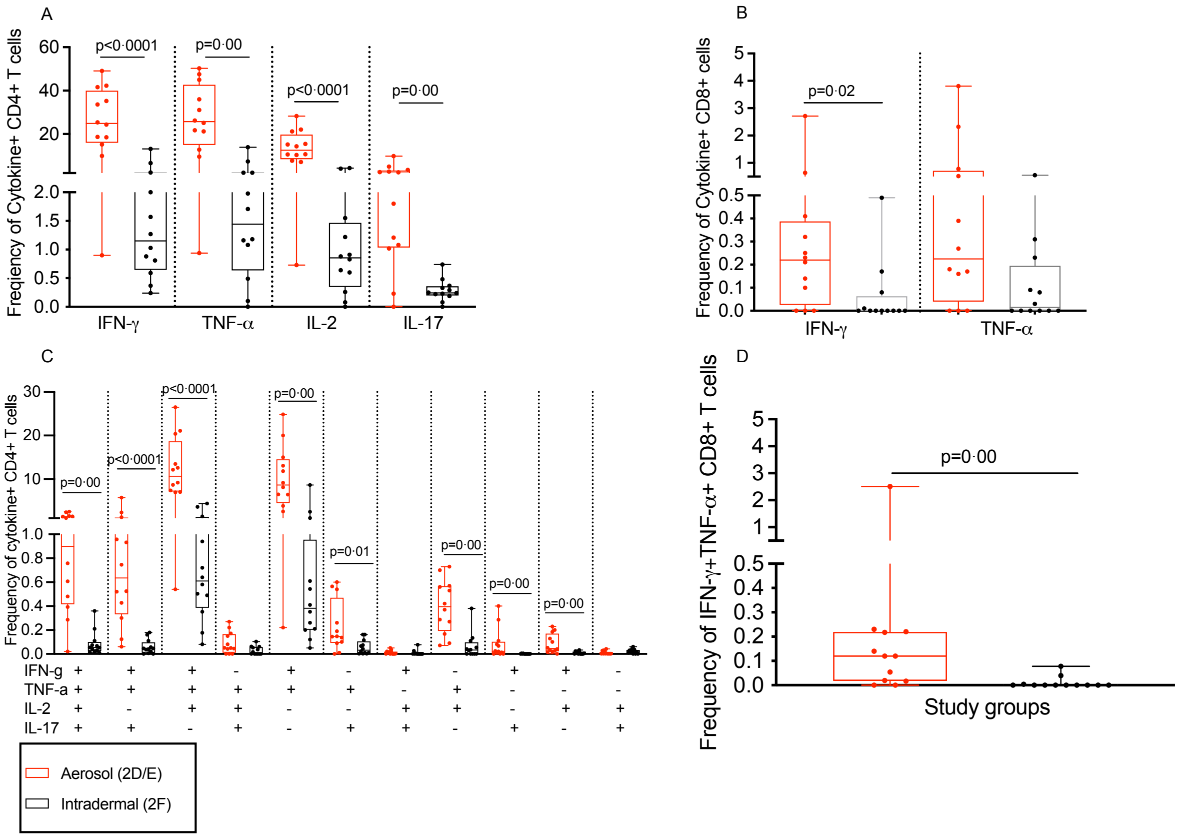

Appendix Figure 7

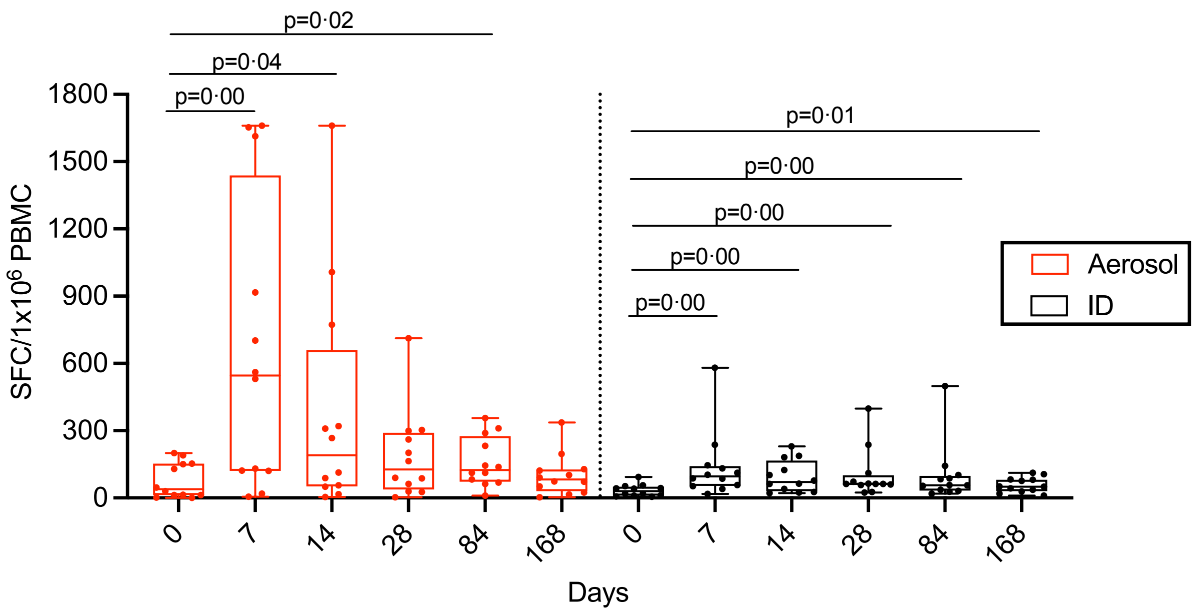

Appendix Figure 8

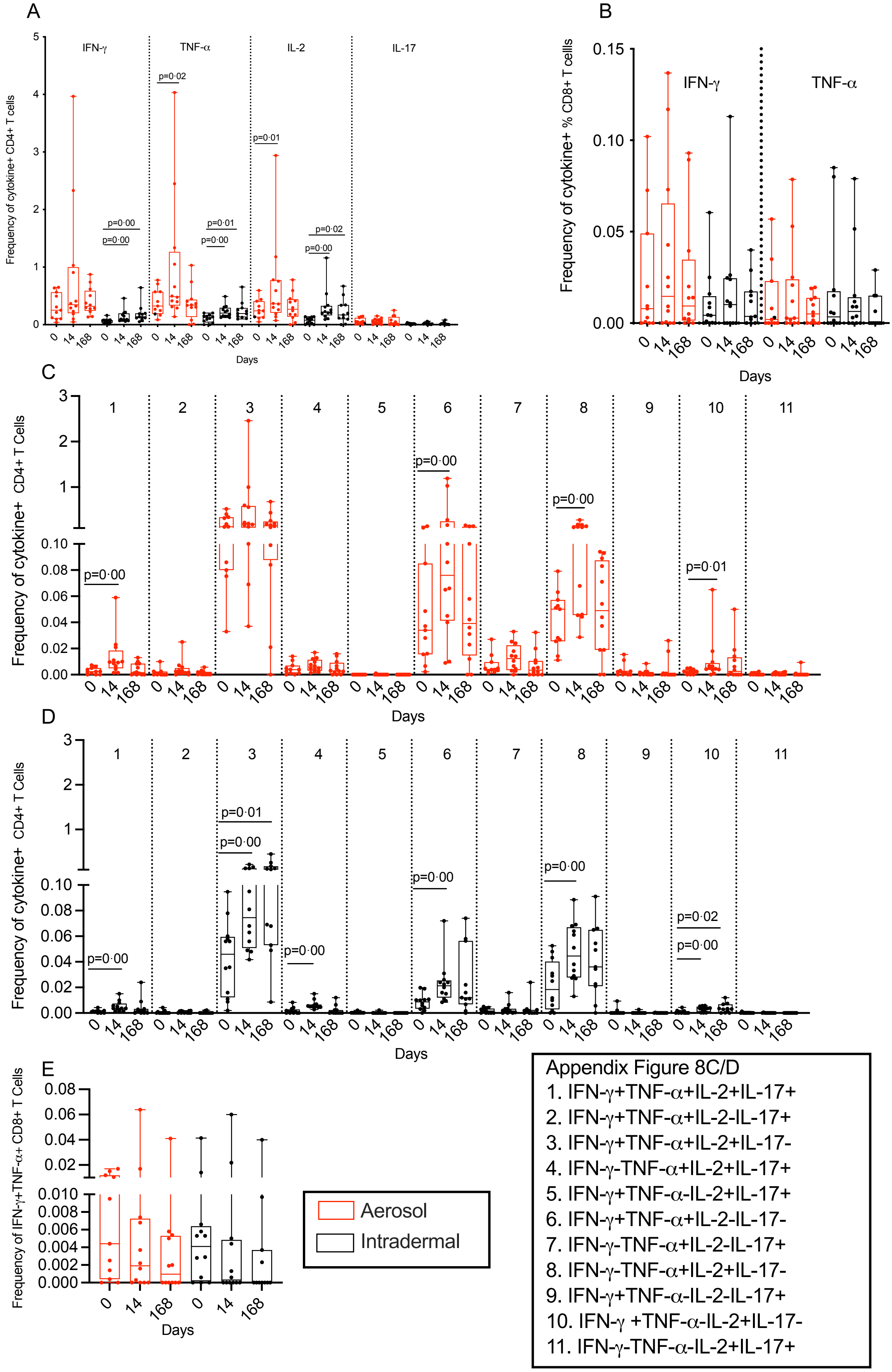

Appendix Figure 9

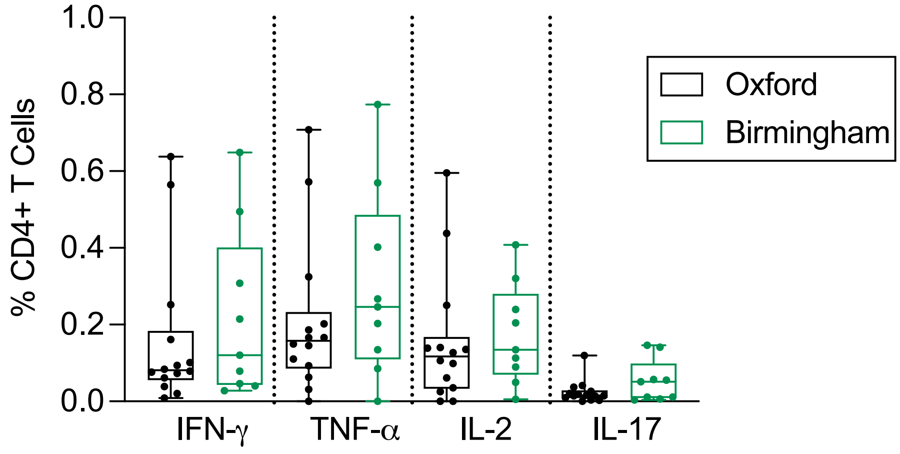

Appendix Figure 10

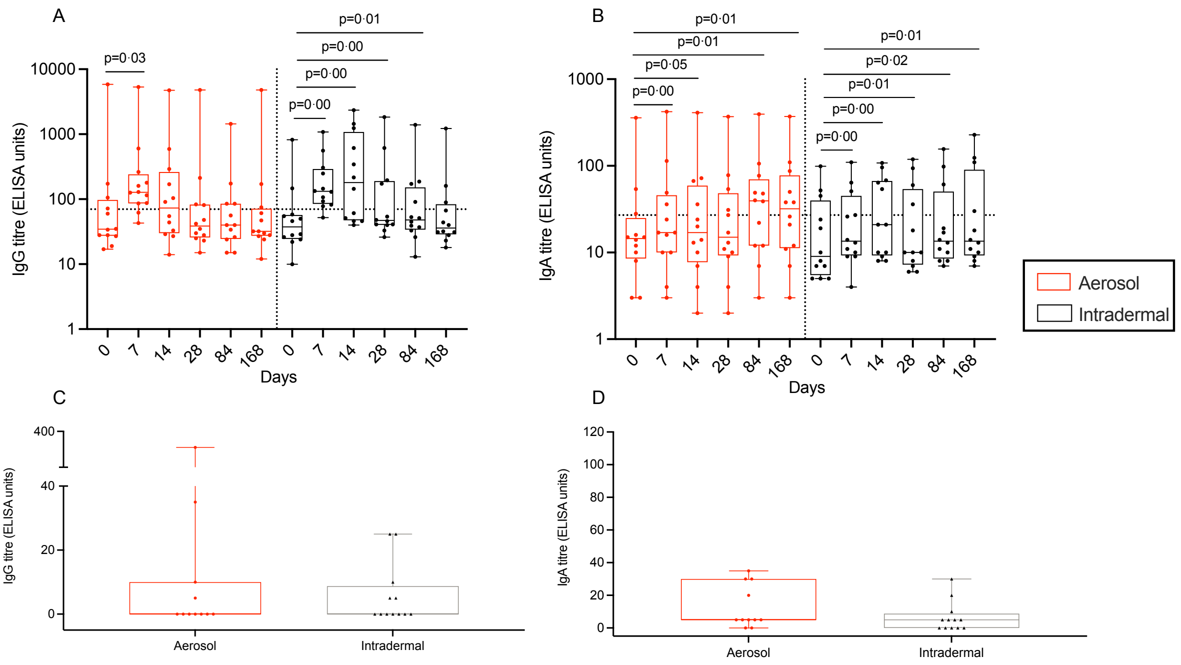

Appendix Tables

Appendix Table 1

Inclusion and exclusion criteria

**Inclusion criteria:**<br>•Healthy adult aged 18-50 years<br>•Resident in or near Oxford (CCVTM or OUH) or Birmingham (NIHR-WTCRF) for the duration of the trial period<br>•Screening IGRA negative<br>•Chest radiograph normal<br>•No relevant findings in medical history or on physical examination<br>•Allow the Investigators to discuss the individual's medical history with their GP<br>•Use effective contraception (see below) for the duration of the trial period (females only)<br>•Refrain from blood donation during the trial<br>•Give written informed consent<br>•Allow the Investigator to register volunteer details with a confidential database (The Over-volunteering Protection Service) to prevent concurrent entry into clinical studies/trials<br>•Able and willing (in the Investigator's opinion) to comply with all the trial requirements
**Exclusion Criteria**<br>•Previously resident for more than 12 months concurrently in a tropical climate where significant non-tuberculous mycobacterial exposure is likely<br>•Participation in another research trial involving receipt of an investigational product in the 30 days preceding enrolment, or planned use during the trial period<br>•Prior vaccination with BCG or any candidate TB vaccine<br>•Administration of immunoglobulins and/or any blood products within the three months preceding the planned trial challenge date<br>•Clinically significant history of skin disorder, allergy, atopy, immunodeficiency (including HIV), cancer (except BCC or CIS), cardiovascular disease, gastrointestinal disease, liver disease, renal disease, endocrine disorder, neurological illness, psychiatric disorder, drug or alcohol abuse<br>•Concurrent oral or systemic steroid medication or the concurrent use of other immunosuppressive agents<br>•History of anaphylaxis to vaccination or any allergy likely to be exacerbated by any component of the trial agent, sedative drugs, or any local or general anaesthetic agents<br>•Pregnancy, lactation or intention to become pregnant during trial period<br>•Any respiratory disease, including asthma<br>•Current smoker<br>•Clinically significant abnormality on screening chest radiography<br>•Clinically significant abnormality of spirometry<br>•Any nasal, pharyngeal, or laryngeal finding which precludes bronchoscopy<br>•Current use of any medication taken through the nasal or inhaled route including cocaine or other recreational drugs<br>•Clinical, radiological, or laboratory evidence of current active TB disease<br>•Past treatment for TB disease<br>•Any clinically significant abnormality of screening blood or urine tests<br>•Positive HBsAg, HCV or HIV antibodies<br>•Any other significant disease, disorder, or finding, which, in the opinion of the Investigator, may either put the volunteer at risk, affect the volunteer's ability to participate in the trial or impair interpretation of the trial data*.<br>•If they were concurrently involved in another study or trial that involved regular blood tests or an investigational medicinal product**.<br>•Suffering from a previously undiagnosed condition thought to require further medical attention***.

*This criteria also outlined the process for the investigator to determine if the conditions listed above were classified as “clinically significant”, meriting exclusion of the volunteer.
** In order to check this, volunteers were asked to provide their UK National Insurance or Passport number and were registered on a national database of participants in clinical trials (www.tops.org.uk).
***These were referred to their GP or an NHS specialist service as appropriate for further investigation and treatment.

Appendix Table 2
Protocol deviations

CCVTM, Oxford			
**Deviation #**	**Volunteer**	**Visit**	**Reason/Comment**
1	0411002	D28	Biochemistry results missing due to miscommunication with the lab. Blood test was repeated within the allowed time window.
2	0411005	D84	Visit 21 days out of widow. Volunteer moved out from Oxford for personal reasons and was unable to attend the clinic on the day the visit was due or within the allowed time window period.
3	0411019	D0	Additional blood sample of 5 mL was taken by mistake. This didn't affect participant safety and health. Participant was informed of the deviation.
4	0411019	D28	The full blood count (FBC) sample was missed by mistake. The sample was finally taken 1 day out of window with no safety concern for the volunteer.
5	0411023 and 0411024	D0	As part of the blood samples required as per protocol, a CRP (1x3ml Biochemistry tube) was taken in error for each volunteer. No safety issues occurred as a consequence of this deviation as the amount of blood taken was minimal.
6	0411025	D0	As part of the pre-vaccination plan, 60 ml of blood should be taken for immunology, 5x10ml green (plasma) tubes and 1x10ml red (serum tube). For volunteer 0411025 there were only 4x10ml green tubes and 1x10ml red tube in the bag that came to the lab. It was unclear if that blood sample was taken and the tube missed or if it was not taken. After this incident and as a preventive action, the blood samples taken were always checked against the CRF Form.
7	0411025	D84	Participant declined to go ahead with D84 Induced Sputum procedure. Integrity of the data has not been compromised, as this was one isolated event.
8	0411033	D14	Formal Urine B-HCG test done in order to exclude pregnancy not done on day of Bronchoscopy due to volunteers not able to produce the sample. The clinician reviewed the volunteers and deemed the risk of pregnancy was minimal.
9	0411031	D84	Volunteer was re-consented to PIS version 3.0 dated 17 Nov 2017 on 20 Dec 2017 at their D84 visit. During the re-consent process, the volunteer wrote a 'Y' in the boxes on the consent form, instead of initialling them. The error was noticed during a review of the Study CRFs after the trial was completed and so it was not possible to correct it. Senior Research Nurse and TB trial Lead aware. There are no safety concerns as a result of this deviation.
**NIHR-WTCRF, Birmingham**			
**Deviation #**	**Volunteer**	**Visit**	**Reason/Comment**
1	0415501	D28	Spirometry test not completed due to spirometer not being charged.
2	0415502	N/A	Participant attended the clinic for screening T-Spot test and repeat FBC blood tests, due to initial FBC showing low platelet count. In error full screening bloods were taken and sent to local labs. A total of extra 13.5 mls of blood was taken. There was no harm to the participant and they were informed of the error.

3	0415506	D2	Day 2 visit only requires a CRP but in error a biochemistry profile was sent, as this was needed in previous visits.
4	0415507	D2	Additional blood sample of 4 mL was taken by mistake due to manual not been very clear.
5	0415507	D2	Day 2 CRP blood results came back as CK results due to an error in the request form. Labs had already discarded the blood sample before the error was spotted hence a CRP could not be performed.
6	Version Control	N/A	VIS v 1.0 arm 1 was incorrectly dated. Error in version control
7 & 8	0415513 and 0415515	D7	Liver function tests (LFTs) at follow up visit "Day 7" were not performed for these volunteers. The correct volume of blood was collected as per protocol and the source document (paper CRF) was marked by the site staff member as being ordered. It is therefore unclear whether these tests were not ordered by research staff or if they were ordered but not performed by the lab staff. As corrective action, all processes were reviewed.

Appendix Table 3

Appendix Table 3A. Arm-1: Unsolicited adverse events following BCG Danish aerosol challenge

Unsolicited adverse events that were deemed probably or definitely related to either the aerosol BCG challenge or any other study procedure are presented here.

Group	Subject ID	Intervention	Adverse Events	Time-point	Duration	Grading	Causality (aerosol BCG)	Causality (study procedures)
1B	TBT-0411003	$1x10^4$ CFU aerosol BCG Danish	Increased WCC ($13.65x10^9$/L;) and CRP (102.7mg/L) consistent with concurrent pneumonia	Day 14	7 days	1	Unlikely	Probable
			Diastolic hypertension during pneumonia	Day 15	Resolved by Day 28	1	Unlikely	Probable
1C	TBT-0411007	$1x10^5$ CFU aerosol BCG Danish; ID saline	Injection site pain	Day 0	1 day	1	Not related	Definitely
			Skin irritation possibly at venepuncture site	Day 2	4 days	1	Unlikely	Probable

Appendix Table 3B. Arm-2: Unsolicited adverse events following BCG Bulgaria aerosol challenge

Unsolicited adverse events that were deemed probably or definitely related to either the aerosol BCG challenge or any other study procedure are presented here or if not included in the body of the manuscript.

Group	Subject ID	Intervention	Adverse Events	Time-point	Duration	Grading	Causality (aerosol BCG)	Causality (study procedures)
2A	TBT-0411019	$1x10^4$ CFU aerosol BCG	Dyspnoea and pleuritic right sided chest pain on background of viral URTI. A+E attendance on advice of 111 hence graded 2 but otherwise volunteer would have graded pain and dyspnoea as 1. Diagnosed in A+E as pleurisy secondary to a viral infection	Day 15	1 day	2	Possible	Possible
2B	TBT-0411021	$1x10^5$ CFU aerosol BCG	Dizziness following phlebotomy	Day 15	Few minutes	1	Not related	Definitely
2B	TBT-0411023	$1x10^5$ CFU aerosol BCG	Anaemia: Hb 112 g/L at Day 14 visit	Day 14	Resolved by Day 28	1	Unlikely	Probable
2C	TBT-0411031	$1x10^6$ CFU aerosol BCG	Syncope after blood taken while fasting pre-bronchoscopy	Day 14	Minutes	3	Unlikely	Definitely
2D	TBT-0411033	$1x10^7$ CFU aerosol BCG	Cold symptoms/rhinorrhoea Associated with general malaise, chest pain and shortness of breath	Day 8	4 days	2	Possible	Unlikely
			Dizziness	Day 9	2 days	3	Possible	Unlikely
			Tachycardia	Day 9	2 days	2	Possible	Unlikely
			Loss of consciousness-assessed by paramedics at the scene. No treatment given	Day 10	Few minutes	3	Possible	Unlikely
			Tightness in chest	Day 10	2 days	2	Possible	Unlikely
			Laryngitis ("lost voice")	Day 13	1 days	2	Possible	Unlikely
2E	TBT-0415516	$1x10^7$ CFU aerosol BCG; ID saline	Anaemia: Hb 113 g/L at Day 7 visit	Day 7	Resolved by Day 14	1	Unlikely	Probable
			Sensation of throat swelling (volunteer states common symptoms when unwell). No SOB, normal examination and vital signs	Day 1	2 days	1	Probable	Not related

			CRP increased to 70 mg/L with concurrent "flu-like symptoms"	Day 2	Resolved by Day 7	Not gradable	Probable	Not related
2E	TBT-0411057	1x10^7 CFU aerosol BCG; ID saline	Bad taste	Day 0	< 12 hours	2	Probable	Not related
			Hypersensitivity pneumonitis<br>Likely secondary to BCG inhalation. Symptoms: Flu like illness, Chest tightness, CRP increased to 90 mg/L, SOBOE recorded in e-diary D1-D7, SaO2 in clinic normal. Respiratory viral PCR negative on nasal swab. TLCO 66.80 predicted (D8). No baseline. Repeat TLCO 1 month later was low normal. Pneumonitis deemed resolved by NHS respiratory consultant	Day 0	2 months (51 days)	3	Probable	Not related
			Increased WCC (11.65x10^9/L) on background of possible hypersensitivity pneumonitis	Day 14	Resolved Day 21	1	Probable	Not related

Intradermal (ID); Colony Forming Units (CFU). Normal reference ranges: Haemoglobin (Hb) 120-150 g/L; White Cell Count (WCC): 4.0-11.0x10^9/L; C-Reactive Protein (CRP): <5mg/L.

Appendix Table 4
Percentiles, medians and interquartile ranges

Appendix Table 4A. Frequency of total, systemic or respiratory AEs following aerosol or intradermal BCG Bulgaria infection. % of AEs by volunteer

		25% Percentile	Median	75% Percentile
Total AE	Aerosol	3·25	7	14·5
	Intradermal	1	4	11
		25% Percentile	Median	75% Percentile
Systemic AE	Aerosol	2·25	6·50	11
	Intradermal	1·25	3·50	12·75
		25% Percentile	Median	75% Percentile
Respiratory AE	Aerosol	0·50	7	18·75
	Intradermal	1·25	3·50	8·75

*** Appendix Table 4B. Frequency of solicited respiratory AEs reported between blinded (Group 2E) and unblinded (Group 2D) volunteers following 1x107CFU aerosol BCG Bulgaria inhalation**

	25% Percentile	Median	75% Percentile
Group 2D	19	22	38
Group 2E	0	5	9

*** Appendix Table 4C. BCG quantified by culture on solid agar plates: BCG CFU recovered from the vaccine vials used in BCG Bulgaria infected study groups**

	25% Percentile	Median	75% Percentile
Group 2A	733000	1600000	2150000
Group 2B	533000	1151500	1770000
Group 2C	439000	453000	840000
Group 2D/E	217500	440000	765750
Group 2F	301750	400500	723250

*** Appendix Table 4D. BCG quantified by culture on solid agar plates: BCG CFU per dose determined from the diluted/concentrated vials following volunteer infection, plated onto Middlebrook 7H11 agar**

	25% Percentile	Median	75% Percentile
Group 2A	1850	2767	3330
Group 2B	7330	26315	45300
Group 2C	110000	113000	210000
Group 2D/E	543500	1100000	1897500
Group 2F	75475	100150	181000

*** Appendix Table 4E. BCG recovery in bronchoalveolar lavage (BAL) Mycobacterial Growth Indicator Tube (MGIT) culture data is presented as time to positivity (TTP) in volunteers in the aerosol-inhaled BCG "Bulgaria" groups of the study**

	25% Percentile	Median	75% Percentile
Group 2B	473	770	1037
Group 2C	300	403	553
Group 2D/E	266	499	607.5

Appendix Table 4F. Bronchoalveolar Lavage CD4+ T cell cytokines

		25% Percentile	Median	75% Percentile
Aerosolised BCG	IFN-γ	16·01	24·88	40·01
	TNF-α	14·91	25·72	42·74
	IL-2	8·42	12·56	19·81
	IL-17	1·04	2·08	3·27
Intradermal BCG	IFN-γ	0·65	1·15	2·23
	TNF-α	0·64	1·45	2·25
	IL-2	0·35	0·86	1·47
	IL-17	0·20	0·24	0·36

Appendix Table 4G. Bronchoalveolar Lavage CD8+ T cell cytokines

		25% Percentile	Median	75% Percentile
Aerosolised BCG	IFN-γ	0·03	0·22	0·39
	TNF-α	0·04	0·23	0·72
Intradermal BCG	IFN-γ	0	0	0·06
	TNF-α	0	0·02	0·20

Appendix Table 4H. Bronchoalveolar Lavage Polyfunctional CD4+ T cells: Aerosol inhaled BCG

	25% Percentile	Median	75% Percentile
IFN-γ+ TNF-α+ IL-2+ IL-17+	0·41	0·90	1·492
IFN-γ+ TNF-α+ IL-2- IL-17+	0·33	0·64	1·17
IFN-γ+ TNF-α+ IL-2+ IL-17-	7·12	10·65	18·65
IFN-γ- TNF-α+ IL-2+ IL-17+	0·01	0·05	0·1658
IFN-γ+ TNF-α- IL-2+ IL-17+	0	0·01	0·01925
IFN-γ+ TNF-α+ IL-2- IL-17-	4·49	8·64	14·52
IFN-γ- TNF-α+ IL-2- IL-17+	0·09	0·15	0·47
IFN-γ- TNF-α+ IL-2+ IL-17-	0·19	0·40	0·57
IFN-γ+ TNF-α- IL-2- IL-17+	0·00	0·02	0·10
IFN-γ+ TNF-α- IL-2+ IL-17-	0·02	0·05	0·17
IFN-γ- TNF-α- IL-2+17+	0	0	0·02

Appendix Table 4I. Bronchoalveolar Lavage Polyfunctional CD4+ T cells: Intradermal BCG

	25% Percentile	Median	75% Percentile
IFN-γ+ TNF-α+IL-2+ IL-17+	0·025	0·05	0·10
IFN-γ+ TNF-α+ IL-2- IL-17+	0·01	0·04	0·10
IFN-γ+ TNF-α+ IL-2+ IL-17-	0·39	0·61	1·31
I IFN-γ- TNF-α+ IL-2+ IL-17+	0	0	0·06
IFN-γ+ TNF-α- IL-2+ IL-17+	0	0	0
IFN-γ+ TNF-α+ IL-2- IL-17-	0·20	0·38	1
IFN-γ- TNF-α+ IL-2- IL-17+	0	0·03	0·10
IFN-γ- TNF-α+ IL-2+ IL-17-	0	0·03	0·10
IFN-γ+ TNF-α- IL-2- IL-17+	0	0	0
IFN-γ+ TNF-α- IL-2+ IL-17-	0	0	0·02
IFN-γ- TNF-α- IL-2+17+	0	0·02	0·03

Appendix Table 4J. Bronchoalveolar Lavage Polyfunctional CD8+ T cells

	25% Percentile	Median	75% Percentile
Aerosolised BCG	0·02	0·12	0·22
Intradermal BCG	0	0	0

Appendix Table 4K. *Ex-vivo* IFN-γ ELISpot

	Day	25% Percentile	Median	75% Percentile
Aerosolised BCG	D0	13	38·5	152·50
	D7	121·30	546	1439
	D14	51·50	190·5	659·80
	D28	37·25	127	290·30
	D84	72·50	125	275·80
	D168	30·75	82·50	126·50
Intradermal BCG	D0	10·50	30·50	50·75
	D7	54·25	95·50	142·50
	D14	30·25	71	166·80
	D28	58·50	63	101·30
	D84	33·25	57	98·25
	D168	30·75	50·50	81·75

Appendix Table 4L. Whole blood intracellular cytokine staining. CD4+ T Cells: total cytokines

Cytokine	Group	Day	25% Percentile	Median	75% Percentile
CD4+ T cells IFN-γ	Aerosolised BCG	D0	0·10	0·25	0·57
		D14	0·20	0·35	1.00
		D168	0·24	0·32	0·59
	Intradermal BCG	D0	0·03	0·07	0·08
		D14	0·07	0·09	0·20
		D168	0·12	0·14	0·20
Cytokine	Group	Day	25% Percentile	Median	75% Percentile
CD4+ T cells TNF-α	Aerosolised BCG	D0	0·19	0·32	0·57
		D14	0·33	0·49	1·26
		D168	0·13	0·34	0·44
	Intradermal BCG	D0	0·04	0·12	0·16
		D14	0·15	0·20	0·29
		D168	0·13	0·19	0·29
Cytokine	Group	Day	25% Percentile	Median	75% Percentile
CD4+ T cells IL-2	Aerosolised BCG	D0	0·13	0·24	0·41
		D14	0·21	0·36	0·77
		D168	0·14	0·27	0·44
	Intradermal BCG	D0	0·01	0·08	0·13
		D14	0·16	0·22	0·33
		D168	0·10	0·17	0·34
Cytokine	Group	Day	25% Percentile	Median	75% Percentile
CD4+ T cells IL-17	Aerosolised BCG	D0	0·03	0·05	0·12
		D14	0·03	0·06	0·09
		D168	0	0·02	0·14
	Intradermal BCG	D0	0	0·01	0·02
		D14	0·01	0·01	0·03
		D168	0	0·02	0·02

Appendix Table 4M. Whole blood intracellular cytokine staining. CD8+ T Cells: total cytokines

Cytokine	Group	Day	25% Percentile	Median	75% Percentile
CD8+ T cells IFN-γ	Aerosolised BCG	D0	0	0·01	0·05
		D14	0	0·01	0·07
		D168	0	0·01	0·03
	Intradermal BCG	D0	0	0	0·01
		D14	0	0	0·02
		D168	0	0	0·02
Cytokine	Group	Day	25% Percentile	Median	75% Percentile
CD8+ T cells TNF-α	Aerosolised BCG	D0	0	0	0·02
		D14	0	0	0·02
		D168	0	0	0·01
	Intradermal BCG	D0	0	0	0·02
		D14	0	0·01	0·01
		D168	0	0	0·02

Appendix Table 4N. Polyfunctional CD4+ T Cells: Aerosolised BCG

Cytokines	Day	25% Percentile	Median	75% Percentile
IFN-γ+ TNF-α+IL-2+IL-17+	D0	0	0	0
	D14	0	0·01	0·02
	D168	0	0	0·01
Cytokines	Day	25% Percentile	Median	75% Percentile
IFN-γ+ TNF-α+IL-2-IL-17+	D0	0	0	0
	D14	0	0	0
	D168	0	0	0
Cytokines	Day	25% Percentile	Median	75% Percentile
IFN-γ+ TNF-α+IL-2+IL-17-	D0	0·08	0·13	0·34
	D14	0·11	0·19	0·59
	D168	0·09	0·15	0·25
Cytokines	Day	25% Percentile	Median	75% Percentile
IFN-γ- TNF-α+IL-2+IL-17+	D0	0	0	0·01
	D14	0	0	0·01
	D168	0	0	0·01
Cytokines	Day	25% Percentile	Median	75% Percentile
IFN-γ+ TNF-α-IL-2+IL-17+	D0	0	0	0
	D14	0	0	0
	D168	0	0	0
Cytokines	Day	25% Percentile	Median	75% Percentile
IFN-γ+ TNF-α+IL-2-IL-17-	D0	0·02	0·03	0·09
	D14	0·04	0·08	0·25
	D168	0·01	0·04	0·13
		25% Percentile	Median	75% Percentile
IFN-γ- TNF-α-IL-2-IL-17+	D0	0·00	0	0·01
	D14	0·00	0	0·02
	D168	0	0	0·01
Cytokines	Day	25% Percentile	Median	75% Percentile
IFN-γ- TNF-α+IL-2+IL-17-	D0	0·03	0·05	0·06
	D14	0·05	0·12	0·16
	D168	0·02	0·05	0·09
Cytokines	Day	25% Percentile	Median	75% Percentile
IFN-γ+ TNF-α-IL-2-IL-17+	D0	0	0	0
	D14	0	0	0
	D168	0	0	0
Cytokines	Day	25% Percentile	Median	75% Percentile
IFN-γ+ TNF-α-IL-2+IL-17-	D0	0	0	0

	D14	0	0	0·01
	D168	0	0	0
Cytokines	Day	25% Percentile	Median	75% Percentile
IFN-γ- TNF-α-IL-2+IL-17+	D0	0	0	0
	D14	0	0	0
	D168	0	0	0

Appendix Table 4O. Polyfunctional CD4+ T Cells: Intradermal BCG

Cytokines	Day	25% Percentile	Median	75% Percentile
IFN-γ+ TNF-α+IL-2+IL-17+	D0	0	0	0
	D14	0	0	0·01
	D168	0	0	0
Cytokines	Day	25% Percentile	Median	75% Percentile
IFN-γ+ TNF-α+IL-2-IL-17+	D0	0	0	0
	D14	0	0	0
	D168	0	0	0
		25% Percentile	Median	75% Percentile
IFN-γ+ TNF-α+IL-2+IL-17-	D0	0·01	0·05	0·06
	D14	0·05	0·08	0·13
	D168	0·05	0·11	0·18
Cytokines	Day	25% Percentile	Median	75% Percentile
IFN-γ- TNF-α+IL-2+IL-17+	D0	0	0	0
	D14	0	0·01	0·01
	D168	0	0	0
Cytokines	Day	25% Percentile	Median	75% Percentile
IFN-γ+ TNF-α-IL-2+IL-17+	D0	0	0	0
	D14	0	0	0
	D168	0	0	0
Cytokines	Day	25% Percentile	Median	75% Percentile
IFN-γ+ TNF-α+IL-2-IL-17-	D0	0	0·01	0
	D14	0·01	0·02	0·03
	D168	0·01	0·01	0·06
Cytokines	Day	25% Percentile	Median	75% Percentile
IFN-γ- TNF-α-IL-2-IL-17+	D0	0	0	0
	D14	0	0	0
	D168	0	0	0
Cytokines	Day	25% Percentile	Median	75% Percentile
IFN-□- TNF-α+IL-2+IL-17-	D0	0	0·02	0
	D14	0·03	0·04	0·07
	D168	0·021	0·04	0·07
Cytokines	Day	25% Percentile	Median	75% Percentile
IFN-γ+ TNF-α-IL-2-IL-17+	D0	0	0	0
	D14	0	0	0
	D168	0	0	0
Cytokines	Day	25% Percentile	Median	75% Percentile
IFN-γ+ TNF-α-IL-2+IL-17-	D0	0	0	0

	D14	0	0	0
	D168	0	0	0·01
Cytokines	Day	25% Percentile	Median	75% Percentile
IFN-γ- TNF-α-IL-2+IL-17+	D0	0	0	0
	D14	0	0	0
	D168	0	0	0

Appendix Table 4P. Polyfunctional CD8+ T Cells

Group	Cytokines	Day	25% Percentile	Median	75% Percentile
Aerosolised BCG	IFN-γ+ TNF-α+	D0	0	0	0
		D14	0	0	0·01
		D168	0	0	0·01
Intradermal BCG	IFN-γ+ TNF-α+	D0	0	0	0·01
		D14	0	0	0
		D168	0	0	0

Appendix Table 4Q. Serum IgG

Group	Day	25% Percentile	Median	75% Percentile
Aerosolised BCG	D0	27·25	34·50	97·50
	D7	86·25	127·50	241·30
	D14	30	73	261·50
	D28	25·75	38·50	82·50
	D84	24·50	40	85
	D168	27·25	32	71·50
Intradermal BCG	D0	24·50	37·50	57·25
	D7	84·75	132	291·80
	D14	47·25	180	1081
	D28	40·25	47	190·30
	D84	34	48	151·80
	D168	28·75	36	83·50

Appendix Table 4R. Serum IgA

Group	Day	25% Percentile	Median	75% Percentile
Aerosolised BCG	D0	8·50	14·50	25
	D7	10	17	45·75
	D14	7·75	17	59·25
	D28	9·25	15	48·25
	D84	12	39·50	70
	D168	11·25	32	77·75
Intradermal BCG	D0	5·50	9	39·75
	D7	9·25	13·50	45
	D14	9·25	21	67
	D28	7·25	10	54
	D84	8·50	13·50	50·50
	D168	9·25	13·50	90

Appendix Table 4S. Bronchoalveolar Lavage IgG

Group	25% Percentile	Median	75% Percentile
Aerosolised BCG	0	0	10
Intradermal BCG	0	0	8·75

Appendix Table 4T. Bronchoalveolar Lavage IgA

Group	25% Percentile	Median	75% Percentile
Aerosolised BCG	5	5	30
Intradermal BCG	0	5	8·75

*Study groups
2A: $1x10^4$ CFU aerosol-inhaled BCG Bulgaria (InterVax)
2B: $1x10^5$ CFU aerosol-inhaled BCG Bulgaria (InterVax)
2C: $1x10^6$ CFU aerosol-inhaled BCG Bulgaria (InterVax)
2D: $1x10^7$ CFU aerosol-inhaled BCG Bulgaria (InterVax)
2E: $1x10^7$ CFU aerosol-inhaled BCG Bulgaria (InterVax), and intradermal saline placebo
2F: Aerosol-inhaled saline placebo, and $1x10^6$ CFU intradermal BCG Bulgaria (InterVax)
"Aerosolised BCG": Groups 2D&2E
"Intradermal BCG": Group 2F

Appendix Table 5

Solicited Adverse Events (AEs) in the two weeks following bronchoscopy

BCG Bulgaria comparator groups. Aerosol: Group 2D/2E BCG Bulgaria $1x10^7$CFU aerosol; ID: Group 2F BCG Bulgaria $1x10^6$CFU intradermal.

**Adverse Events (AEs)**		**BCG Aerosol** n=12	**BCG ID** n=12	**p-value**
% solicited occurrences, median (IQR)				
	Respiratory AEs	3 (1·25-11·25)	5·50 (2·50-12·75)	0·50
	Systemic AEs	2 (0-3)	3·50 (1·25-9·75)	0·09
% volunteers to report mild AEs, median (IQR)				
	Respiratory AEs	100 (44-100)	100 (92-100)	0·23
	Systemic AEs	100 (55-100)	87 (69-100)	0·90

Clinical Trial Protocol

CLINICAL TRIAL PROTOCOL

A clinical challenge trial to evaluate controlled human infection with BCG administered by the aerosol inhaled route compared with the intradermal route in healthy, BCG-naïve, UK adult volunteers

Short title: Aerosol BCG challenge trial in healthy UK adults

**Trial Reference**:	TB041
**EudraCT number:**	2015-004981-27
**REC Reference:**	15/SC/0716
**IRAS Reference:**	192512
**Date and Version Number**	12 October 2017, V5.0
**Chief Investigator:**	Professor Helen McShane
**Sponsor:**	University of Oxford
**Local Safety Committee Chair:**	Professor Brian Angus
**Funding body:**	The Gates Foundation and The Wellcome Trust
**Author:**	Dr Michael Riste, Dr Julia Marshall

Confidentiality Statement
This document contains confidential information that must not be disclosed to anyone other than the Sponsor, the Investigator Team, and members of the Research Ethics Committee, unless authorised to do so. This information cannot be used for any purpose other than the evaluation or conduct of the clinical investigation without the prior written consent of Professor Helen McShane.

TABLE OF CONTENTS

1 STATEMENT OF COMPLIANCE 43

2 AMENDMENT HISTORY 44

3 KEY TRIAL CONTACTS AND ROLES 47

4 SYNOPSIS 49

4.1 SYNOPSIS 49
4.2 SCHEDULE OF VISITS AND PROCEDURES 51

5 ABBREVIATIONS 52

6 BACKGROUND AND RATIONALE 53

6.1 CONTEXT 53
CHALLENGE MODELS 53
BASIS FOR THE AEROSOL INHALED ROUTE 54
AEROSOL DELIVERY DEVICES 54
BRONCHOSCOPY AND BRONCHOALVEOLAR SAMPLING 54
6.2 RATIONALE 54
HYPOTHESIS 54
CHALLENGE AGENT AND DOSAGE 55
6.3 RISKS AND BENEFITS 58
POTENTIAL RISKS 58
POTENTIAL BENEFITS 59

7 OBJECTIVES AND OUTCOME MEASURES 59

PRIMARY OBJECTIVE 59

8 PARTICIPANT IDENTIFICATION 60

8.4 TRIAL PARTICIPANTS 60
8.5 INCLUSION CRITERIA 60
8.6 EXCLUSION CRITERIA 60

9 TRIAL DESIGN AND PROCEDURES 61

9.7 TRIAL NUMBERS AND GROUPS 61
9.8 RECRUITMENT 62
9.9 INFORMED CONSENT 62
9.10 SCREENING AND ELIGIBILITY ASSESSMENT 63
9.11 RANDOMISATION AND BLINDING 63
9.12 FOLLOW UP VISITS 64
9.13 TIMEPOINTS 64
9.14 SAMPLE HANDLING 65
9.15 CHALLENGE POSTPONEMENT CRITERIA 65
9.16 BRONCHOSCOPY POSTPONEMENT CRITERIA 65
9.17 DISCONTINUATION / WITHDRAWAL CRITERIA 66
9.18 SAFETY 66
9.19 END OF STUDY DEFINITION 66

10 CHALLENGE AGENT AND DEVICES 66

10.20 BCG DESCRIPTION 66
BCG SSI 66
BCG BULGARIA (INTERVAX) 66
STORAGE OF BCG 67
10.21 DISPENSING AND ADMINISTRATION 67
10.22 SALINE 67
10.23 SALBUTAMOL 67
10.24 MICROAIR NE-U22 67
10.25 ULTRASONIC NEBULISER NE-U780 67
10.26 SEDATIVE & ANAESTHETIC AGENTS FOR BRONCHOSCOPY 68

11 ASSESSMENT OF SAFETY 68

11.27 INTERIM SAFETY REVIEW 68
11.28 DEFINITIONS 68
11.29 FORESEEABLE ADVERSE REACTIONS 69
11.30 EXPECTED SERIOUS ADVERSE EVENTS 69
11.31 CAUSALITY ASSESSMENT 69
11.32 REPORTING PROCEDURES FOR ALL ADVERSE EVENTS 71
11.33 ASSESSMENT OF SEVERITY 71
11.34 PROCEDURES TO BE FOLLOWED IN THE EVENT OF ABNORMAL FINDINGS 72
11.35 LOCAL SAFETY COMMITTEE 72

12 STATISTICS 73

13 DATA MANAGEMENT 73

13.36 SOURCE DATA 73
13.37 ACCESS TO DATA 73
13.38 DATA RECORDING AND RECORD KEEPING 73

14 QUALITY ASSURANCE PROCEDURES 73

14.39 QUALITY ASSURANCE 73
14.40 MONITORING 74

15 SERIOUS BREACHES 74

16 ETHICAL AND REGULATORY CONSIDERATIONS 74

16.41 DECLARATION OF HELSINKI 74
16.42 GOOD CLINICAL PRACTICE 74
16.43 APPROVALS 74
16.44 REPORTING 74
16.45 VOLUNTEER CONFIDENTIALITY 75
16.46 EXPENSES AND BENEFITS 75

17 FINANCE AND INSURANCE 75

17.47 FUNDING 75
17.48 INDEMNITY 75
17.49 INSURANCE 75

18 PUBLICATION POLICY 75

19 REFERENCES 76

TABLES

Table 1. Schedule of trial procedures 51
Table 2. Objectives and Outcome Measures 60
Table 3. Trial groups .... **Error! Bookmark not defined.**
Table 4. Guidelines for assessing the relationship of challenge administration to an AE 70
Table 5. Routinely solicited adverse events 71
Table 6. Severity grading criteria for physical observations. 72
Table 7. Severity grading criteria for respiratory and systemic AEs 72

1 STATEMENT OF COMPLIANCE

Investigator Agreement

"I have read this protocol and agree to abide by all provisions set forth therein. I agree to comply with the International Conference on Harmonisation Tripartite Guideline on Good Clinical Practice."

Professor Helen McShane		
Chief Investigator	Investigator Signature	Date

Conflict of Interest

"According to the Declaration of Helsinki, 2008, I have read this protocol, and declare no conflict of interest with any Investigators"

Professor Helen McShane		
Chief Investigator	Investigator Signature	Date

2 AMENDMENT HISTORY

Protocol v5.0 Substantial Amendment

Section	From	Changed To
Throughout	 <br><br>Group 2D<br><br>Group 2E	Dose escalation to 1 x 10^7 cfu BCG Bulgaria in Arm 2 resulting in the addition of an extra group of 3 volunteers (new Group 2D).<br><br>Group 2E<br><br>Group 2F
3		Key staff updated
4.1, 4.2, 6.1, 9.1, 12	Arm 2 (30 subjects)<br>The optimal dose of aerosol inhaled BCG Bulgaria (InterVax) will be identified from preliminary results obtained from Groups 2B and 2C (prepared dose 1 x 10^5 or 1x 10^6 cfu) for randomisation groups 2D and 2E<br>Group 2D dose to therefore be either 1 x 10^5 or 1x 10^6 cfu<br>Group 2E dose to be the same as Group 2D	Arm 2 (33 subjects)<br>The optimal dose of aerosol inhaled BCG Bulgaria (InterVax) will be identified from preliminary results obtained from Groups 2C and 2D (prepared dose 1 x 10^6 or 1x 10^7 cfu) for randomisation group 2E<br>Group 2E dose to therefore be either 1 x 10^6 or 1x 10^7 cfu<br>Group 2F prepared dose to be one log lower than Group 2E
4.3	 <br>U+Es, LFTs, CRP (except day 2 when CRP only); if raised to be repeated on day 7	Clarification of wording around collection of CRP<br>U+Es, LFTs, CRP (except day 2 when CRP only)
6.2, 9.7, 11.1		Safety review for new Group 2D. To mirror the safety reviews for Groups 2A-2C.
6.2		Addition of rationale for dose escalation to 1x10^7 cfu BCG Bulgaria
6.3, 9.6	Induced sputum procedure Oxford volunteers Groups 2A-2E (ID) at the 4weeks, 3 months, 6 months follow up visits.	Induced sputum procedure Oxford volunteers Groups 2A-2E (not the 2F ID group).<br>Groups 2A-2D and 3 volunteers in 2E will have collection at the day 2, day 7 and 6 months follow up visit; Last 6 volunteers of Group 2E will have collection at the 4weeks, 3 months and 6 months follow up visits
9.5		Explanation that due to the change in schedule of induced sputum collection a small number of volunteers in Group 2E may become unblinded before the day 14 visit.
9.8	Volunteers will be informed that there may be leftover samples of their blood, BAL and sputum.	Volunteers will be informed that there may be leftover samples of their blood, BAL, skin biopsies and sputum.
10.2	• During aerosol inhalation the Investigator will wear appropriate protective clothing (PPE); gown, goggles and face mask.<br>• The hard surfaces within close range (1-2 metres) of aerosol challenge will be cleaned and disinfected in accordance with local recommendations.	• During aerosol inhalation all clinical staff will wear appropriate protective clothing (PPE); gown, goggles, overshoes, gloves and FFP3 face mask. The volunteer will wear a hood in order to contain the aerosol.<br>• The clinic room will then undergo a terminal clean in accordance with local recommendations.
10.3-10.7		Addition of details regarding the agents and devices used as part of the induced sputum procedure; Salbutamol, hypertonic saline and the ultrasonic nebuliser NE-U780
19		2 references added; the European Respiratory Society recommendation for Induced Sputum procedures and the NE-U780 nebuliser manual.

Protocol v4.0 Substantial Amendment

Section	From	Changed To
4.2, 6.3		Addition of footnote to say blood volumes may vary slightly between sites
6.2		Addition of rationale for dose escalation

Protocol v3.0 Substantial Amendment

Section	From	Changed To
3		Key staff updated
Throughout		Addition of Arm 2, using BCG Bulgaria (InterVax)
4, 6.3, 9.6		Addition of Induced Sputum sample
11.1		Clarification that there is no dose escalation and so no safety review for Groups 1C and 1D
16.6		Increase in compensation due to induced sputum sampling necessitating longer visits to clinic

Protocol v2.0 Substantial Amendment

Section	From	Changed To

4.1, 6.3, 7	Intradermal biopsy	Punch biopsy
5		Sii abbreviation removed
6.2	BCG challenge using BCG SSI containing Mycobacterium bovis strain Danish 1331 is preferred as it is licensed in the UK. However BCG SSI can frequently go into global short supply, impacting UK supply. In the event of this occurring, BCG supplied by the SII will be used instead which contains Mycobacterium bovis BCG strain Moscow 361 I and is on the WHO list of prequalified vaccines. The same strain will be used for all volunteers.<br>The licensed dose of BCG SSI and BCG SII vaccine is 2-8 x 10^5 cfu (0.1ml) intradermally.	BCG SSI containing Mycobacterium bovis strain Danish 1331 will be used in this trial; it is licensed in the UK.<br>The licensed dose of BCG SSI vaccine is 2-8 x 10^5 cfu (0.1ml) intradermally.
6.2	The doses which follow will be on the basis of an estimated BCG concentration of 4 x 10^5 cfu.	The doses which follow will be on the basis of an average median BCG concentration of 5 x 10^6 cfu/ml.<br>Further details of safety reviews are given in section 11.1.
6.3	BCG SSI is a licensed vaccine that has been given to over 3 billion people throughout the world since it was first developed in 1921. Full details are given in the SmPC. BCG SII is on the WHO list of pre-qualified vaccines and has a well-defined side effect profile as outlined in the SmPC that is similar to that of BCG SSI. BCG is licensed via the intradermal route. It is not licensed via the aerosol route.	BCG SSI is a licensed vaccine that has been given to over 3 billion people throughout the world since it was first developed in 1921. Full details are given in the SmPC. BCG is licensed for delivery via the intradermal route. It is not licensed for delivery via the aerosol route.
10.1	BCG SSI contains live attenuated Danish strain 1331 Mycobacterium bovis BCG. It is supplied as a powder and solvent for suspension. Each vial contains 2-8 x 10^5 cfu (see SmPC). BCG SII contains live attenuated Mycobacterium bovis BCG Moscow strain 361I. It is supplied as a powder and solvent for suspension. Each vial contains 2-8 x 10^5 cfu (see SmPC).<br>Storage of BCG<br>BCG SSI will be supplied to the CCVTM in Oxford from the Churchill Hospital pharmacy. BCG SII will be shipped to the CCVTM, with shipping at between +2°C and +8°C to ensure cold chain.	BCG SSI contains live attenuated Danish strain 1331 Mycobacterium *bovis* BCG. It is supplied as a powder and solvent for suspension. Each vial contains 2-8 x 10^5 cfu (see SmPC).<br>Storage of BCG<br>BCG SSI will be supplied to the CCVTM in Oxford from the Churchill Hospital pharmacy.
11.1		Dose escalation will not take place in the event of any of the following stopping criteria being met:<br>• **Any SAE deemed to be possibly, probably or definitely related to the challenge agent**<br>• **Solicited respiratory AEs:** If more than one dose of challenge agent (Groups A or B) or more than 25% of doses of challenge agent (Groups C or D) are followed by the same Grade 3 solicited local AE beginning within 2 days after challenge (day of challenge and one subsequent day) and persisting at Grade 3 for >48 hours.<br>• **Solicited systemic AEs:** If more than one dose of challenge agent (Groups A or B) or more than 25% of doses of challenge agent (Groups C or D) are followed by the same Grade 3 solicited systemic AE beginning within 2 days after challenge (day of challenge and one subsequent day) and persisting at Grade 3 for >48 hours.<br>• **Unsolicited AEs:** If more than one volunteer (Groups A or B) or more than 25% of volunteers (Groups C or D) develop a Grade 3 unsolicited AE (including the same laboratory AE) that is considered possibly, probably or definitely related to challenge and persists at Grade 3 for >48 hours.<br><br>'Solicited respiratory AEs' and 'solicited systemic AEs' are those listed in Table 5 in Section 11.6. 'Unsolicited AEs' are any AE not described herein.<br>If a stopping rule has been met and following an internal safety review it is deemed appropriate to restart dosing, a request to restart dosing with pertinent data must be submitted to the regulatory authority as a request for a substantial amendment. The internal safety review will consider:<br><br>• The relationship of the AE or SAE to the challenge agent.

		• The relationship of the AE or SAE to the challenge agent dose, or other possible causes of the event.<br><br>• If appropriate, additional screening or laboratory testing for other volunteers to identify those who may develop similar symptoms, and alterations to the current Participant Information Sheet (PIS).<br><br>• New, relevant safety information from ongoing research programs on the various components of the challenge agent.<br><br>The local ethics committee will also be notified if a holding rule is activated or released.<br><br>In addition to these pre-defined criteria, the study can be put on hold upon advice of the Local Safety Monitor, Chief Investigator, Study Sponsor, regulatory authority, Ethical Committee(s) or Local Safety Committee, for any single event or combination of multiple events which, in their professional opinion, jeopardise the safety of the volunteers or the reliability of the data.
11.6	Outside the diary card periods, respiratory and systemic AEs (listed in section 6.3)<br>**Reporting Procedures for SARs**<br>As BCG is a vaccine with Marketing Authorisation, the mechanism for reporting any SARs to the MHRA is via yellow card.	Outside the diary card periods, respiratory and systemic AEs (listed in Table 5, below)<br>Addition of Table 5<br>**Reporting Procedures for SARs**<br>These will be reported as per any SAE.

3 KEY TRIAL CONTACTS AND ROLES

**Clinical Trial Units:**	Centre for Clinical Vaccinology and Tropical Medicine (CCVTM)<br>Churchill Hospital,<br>Old Road<br>Headington<br>Oxford, OX3 7LE<br><br>Oxford University Hospital NHS Foundation Trust (OUH)<br>(Oxford Centre for Respiratory Medicine, John Warin Ward, Oxford Haemophilia & Thrombosis Centre)<br>Headington<br>Oxford, OX3 7LE<br><br>National Institute for Health Research-Wellcome Trust Clinical Research Facility (NIHR-WTCRF)<br>Queen Elizabeth Hospital, Edgbaston<br>Birmingham, B15 2TH
**Chief Investigator:**	Professor Helen McShane<br>CCVTM<br>Tel: +44 (0)1865 617606<br>Fax: +44 (0)1865 857471<br>Email: helen.mcshane@ndm.ox.ac.uk
**Principal Investigator:**	Prof Paul Moss<br>NIHR-WTCRF<br>Tel: +44 (0)121 414 2824<br>Email: p.moss@bham.ac.uk
**Other Investigators:**	Dr Julia Marshall<br>CCVTM
**Lead Nurse:**	RN Raquel Lopez Ramon<br>CCVTM
**Collaborators:**	Dr Henry Bettinson<br>Consultant Respiratory and Intensive Care Physician<br>Oxford Centre for Respiratory Medicine<br>Oxford University Hospitals NHS Foundation Trust<br>Oxford, OX3 7LE<br>Tel: +44 (0)1865 225234<br>Fax: +44 (0)1865 225221<br>Email: henry.bettinson@ouh.nhs.uk
**Project Managers:**	Mrs Samantha Vermaak, Dr Rebecca Powell-Doherty & Dr Alison Lawrie<br>CCVTM
**Local Safety Monitor:**	Professor Brian Angus<br>Nuffield Department of Medicine, University of Oxford<br>Old Road, Headington<br>Oxford, OX3 7BN<br>Tel: +44 (0)1865 220154<br>Fax: +44 (0)1865 222962<br>Email: brian.angus@ndm.ox.ac.uk
**Senior Immunologist:**	Dr Iman Satti<br>The Jenner Institute

Old Road Campus Research Building
Roosevelt Drive, Headington
Oxford, OX3 7DQ
Tel: +44 (0)1865 617623
Fax: +44 (0)1865 617608
Email: iman.satti@ndm.ox.ac.uk

Statistician:

Nicola Williams
Senior Trial Statistician
Nuffield Department of Primary Care Health Sciences
Radcliffe Observatory Quarter, University of Oxford
Woodstock Road,
Oxford. OX2 6GG.
Tel: +44 (0) 1865 289300.

Sponsor:

Clinical Trials & Research Governance
Joint Research Office, University of Oxford
Block 60, Churchill Hospital
Old Road, Headington
Oxford, OX3 7LE

Monitor:

Clinical Trials & Research Governance
Joint Research Office, University of Oxford
Block 60, Churchill Hospital
Old Road, Headington
Oxford, OX3 7LE

4 SYNOPSIS

4.1 Synopsis

Trial Title	A clinical challenge trial to evaluate controlled human infection with BCG administered by the aerosol inhaled route compared with the intradermal route in healthy UK adult volunteers	
Trial Identifier	TB041	
Chief Investigator	Professor Helen McShane	
Trial Centres	Centre for Clinical Vaccinology and Tropical Medicine (CCVTM), Churchill Hospital, Old Road, Headington, Oxford, OX3 7LE<br><br>Oxford University Hospital NHS Foundation Trust (OUH) (John Warin Ward, Oxford Haemophilia & Thrombosis Centre, Oxford Centre for Respiratory Medicine), Old Road, Headington, Oxford, OX3 7LE<br><br>NIHR-Wellcome Trust Clinical Research Facility, Queen Elizabeth Hospital, Edgbaston, Birmingham, B15 2TH	
Trial participants	Healthy adult volunteers aged 18-50 years	
Planned Sample Size	Arm 1 (30 subjects):<br>Group 1A: 3 volunteers will receive 1 x 10^3 cfu aerosol inhaled BCG SSI, followed by bronchoscopy 14 days later<br>Group 1B: 3 volunteers will receive 1 x 10^4 cfu aerosol inhaled BCG SSI, followed by bronchoscopy 14 days later<br>The next 24 subjects will be randomised to two trial arms:<br>Group 1C: 12 volunteers receiving 1 x 10^5 cfu aerosol inhaled BCG SSI and ID saline placebo, followed by bronchoscopy 14 days later *[NB This group enrolled 4 volunteers before the BCG SSI expired in August 2016. Due to a global shortage of BCG SSI no further volunteers will be enrolled to this Arm]*<br>Group 1D: 12 volunteers receiving aerosol inhaled saline placebo and 1 x 10^5 cfu ID injection of BCG SSI, followed by bronchoscopy and punch biopsy 14 days later *[NB This group enrolled 3 volunteers before the BCG SSI expired in August 2016. Due to a global shortage of BCG SSI no further volunteers will be enrolled to this Arm.]*<br><br>Arm 2 (33 subjects):<br>Group 2A: 3 volunteers will receive 1 x 10^4 cfu aerosol inhaled BCG Bulgaria (InterVax), followed by bronchoscopy 14 days later<br>Group 2B: 3 volunteers will receive 1 x 10^5 cfu aerosol inhaled BCG Bulgaria (InterVax), followed by bronchoscopy 14 days later<br>Group 2C: 3 volunteers will receive 1 x 10^6 cfu aerosol inhaled BCG Bulgaria (InterVax), followed by bronchoscopy 14 days later<br>Group 2D: 3 volunteers will receive 1 x 10^7 cfu aerosol inhaled BCG Bulgaria (InterVax), followed by bronchoscopy 14 days later<br>The next 21 subjects will be randomised to two trial arms:<br>Group 2E: 9 volunteers will receive the optimal dose of aerosol inhaled BCG Bulgaria (InterVax) identified from preliminary results obtained from Groups 2C and 2D, and ID saline placebo, followed by bronchoscopy 14 days later<br>Group 2F: 12 volunteers will receive aerosol inhaled saline placebo and intradermal BCG Bulgaria (InterVax), at a dose a log lower than 2E then bronchoscopy and punch biopsy	
Challenge Schedule	Single challenge at day 0	
Follow-up Duration	24 weeks from challenge day	
Blood Sampling	See visit schedule	
Trial Interventions	Spirometry<br>Challenge by aerosol inhaled route (Groups 1A,1B, 1C, 2A, 2B, 2C, 2D and 2E) or ID route (Group 1D and 2F)<br>Saline placebo by alternative route (Groups 1C, 1D, 2E, 2F)<br>Venepuncture<br>Bronchoscopy and BAL<br>Punch biopsy (Group 1D and 2F)<br>Chest x-ray<br>Induced sputum (Groups 2A-2E)	
Trial Duration	Estimated end date is July 2018	
Planned Trial Period	Planned start date is January 2016	
	Objectives	Outcome Measures
Primary	To evaluate the safety of BCG challenge by the aerosol inhaled route in healthy, BCG-naïve UK adult volunteers	Actively and passively collected data on adverse events
Secondary	To evaluate and compare the amount of BCG recovered from BAL in healthy BCG-naïve adults receiving both aerosol inhaled and ID BCG, and punch biopsy in adults receiving ID BCG	Culture (colony counting) and PCR quantification of BCG in BAL and punch biopsy
Tertiary	To identify laboratory markers of the immune response that correlate with the levels of BCG recovered at the challenge site	Established and exploratory markers of innate, cell mediated and humoral immunity in blood and BAL samples

Quaternary	To evaluate and compare the systemic and mucosal cellular and humoral immunogenicity induced by BCG challenge by the aerosol inhaled and ID route, in healthy volunteers	Laboratory markers of cell mediated and humoral immunity, including *ex-vivo* ELISpot in blood and intracellular cytokine staining in blood and BAL samples
Challenge agent	BCG	
Dose(s)	Arm 1:<br>Group 1A: 1 x 10^3 BCG SSI<br>Group 1B: 1 x 10^4 BCG SSI<br>Groups 1C and 1D: 1 x10^5 BCG SSI<br>Arm 2:<br>Group 2A: 1 x 10^4 BCG Bulgaria (InterVax)<br>Group 2B: 1 x 10^5 BCG Bulgaria (InterVax)<br>Group 2C: 1 x 10^6 BCG Bulgaria (InterVax)<br>Group 2D: 1 x 10^7 BCG Bulgaria (InterVax)<br>Group 2E : 1 x 10^6 or 1 x 10^7 BCG Bulgaria (InterVax)<br>Group 2F: One log lower than the Group 2E dose ie either 1 x 10^5 or 1 x 10^6 BCG Bulgaria (InterVax)	
Route of challenge	Aerosol inhalation by nebuliser or ID injection in the deltoid region of the arm	
Allocation Method	Sequential enrolment (Groups 1A, 1B, 2A, 2B, 2C and 2D), variable block randomisation by sequentially numbered sealed envelopes (Groups 1C, 1D, 2E and 2F)	

4.2 Schedule of visits and procedures

**Visit number**	**1**	**2**	**3**	**4**	**5**	**6**	**7**	**8**
Timeline (days)*	Screening	0 (Challenge)	2	7	14	28	84	168
Timeline (weeks)*	Screening	0		1	2	4	12	24
Time windows (days)			±1	±2	±21	±7	±14	±21
Inclusion/exclusion criteria	X							
Review contra-indications	X	X			X			
Informed consent	X							
Medical history	X	(X)	(X)	(X)	(X)	(X)	(X)	(X)
Physical examination	X	(X)	(X)	(X)	(X)	(X)	(X)	(X)
Vital signs	X	X	X	X	X	X	X	X
Urinalysis	X							
PFTs	X	X	X	X	X	X		
β-HCG urine test	X	X			X			
**BCG Challenge**		X						
**Biopsy (Groups 1D and 2F)**					X			
**Bronchoscopy**					X			
Chest radiograph	X							
Induced sputum (Groups 2A, 2B, 2C, 2D, 2E)						X	X	X
Local & systemic events/reactions		X	X	X	X	X	X	X
Ediary setup		X						
Ediary final review						X		
Biochemistry (4mL)**/***	X		X	X	X	X		
Haematology (2mL)***	X			X	X	X		
Coagulation (4mL)***	X							
HBV, HCV, HIV (10mL)	X							
HLA typing (4mL)		X						
Exploratory immunology incl ELISpot (10-60mL****)	X	X	X	X	X	X	X	X
Blood vol (mL)	30	64	20	56	66	66	60	60
Cumulative blood vol (mL)***	30	94	114	170	236	302	362	422

X Event scheduled to occur
(X) If considered necessary, emphasising any complaint or change in medications
* Timeline is approximate only, as exact timings (± windows periods) of visits relate to the actual (not intended) date of the previous visit
** U&Es, LFTs, CRP (except day 2 when CRP only)
*** Blood volumes stated for Oxford. The exact volumes of blood taken will depend on the local site – cumulative blood volumes for volunteers outside Oxford may be slightly lower or higher than those of Oxford volunteers, due to the use of different volume vacutainers for biochemistry, haematology and serology samples as per local Trust standard procedures
**** 60ml at all visits except screening (10ml), visit 3 (16ml) and visit 4 (50ml)

Table 1. Schedule of trial procedures

5 ABBREVIATIONS

**AE**	Adverse Event
**AR**	Adverse Reaction
**BAL**	Bronchoalveolar Lavage
**BCC**	Basal cell carcinoma
**BCG**	Bacille Calmette-Guérin
**β-HCG**	Beta - Human Chorionic Gonadotrophin
**CCVTM**	Centre for Clinical Vaccinology and Tropical Medicine
**CFU**	Colony-forming unit
**CI**	Chief Investigator
**CIS**	Carcinoma *in situ*
**CRF**	Case Report Form
**DNA**	Deoxyribonucleic Acid
**ELISpot**	Enzyme-linked Immunospot
**FBC**	Full Blood Count
**GCP**	Good Clinical Practice
**GP**	General Practitioner (Family Doctor)
**HBsAg**	Hepatitis B Surface Antigen
**HBV**	Hepatitis B Virus
**HCV**	Hepatitis C Virus
**HIV**	Human Immunodeficiency Virus
**HLA**	Human Leukocyte Antigen
**ICH**	International Committee on Harmonisation
**ID**	Intradermal
**IFN-γ**	Interferon Gamma
**IGRA**	Interferon-Gamma Release Assay
**IM**	Intramuscular
**LFT**	Liver Function Test
**LSC**	Local Safety Committee
**MHRA**	Medicines & Healthcare Regulatory Authority
M.tb	*Mycobacterium tuberculosis*
**MVA**	Modified vaccinia Virus Ankara
**NHS**	National Health Service
**NIHR**	National Institute for Health Research
**OUH**	Oxford University Hospitals
**REC**	Research Ethics Committee
**SAE**	Serious Adverse Event
**SAR**	Serious Adverse Reaction
**SmPC**	Summary of Product Characteristics
**SOP**	Standard Operating Procedure
**SSI**	Statens Serum Institut
**SUSAR**	Suspected Unexpected Serious Adverse Reaction
**TB**	Tuberculosis
**TMF**	Trial Master File
**U&Es**	Urea & Electrolytes
**WTCRF**	Wellcome Trust Clinical Research Facility

6 BACKGROUND AND RATIONALE

6.1 Context

Mycobacterium tuberculosis (*M.tb*) is a pathogen with worldwide preponderance which infects humans causing tuberculosis (TB), a transmissible disease resulting in very high mortality and morbidity. It is estimated that a third of the world's population is latently infected with *M.tb*, and these people carry a 10% lifetime risk of developing active life-threatening disease [1]. In 2013, there were 9 million new cases worldwide and 1.5 million people died of TB [2]. Co-infection with human immunodeficiency virus (HIV) greatly increases risk of TB reactivation and death [3, 4]. Diagnosis is challenging and drug treatment can be prolonged, harmful, costly and complex. For these reasons an effective vaccine is a global public health priority.

The Bacille Calmette-Guérin (BCG) vaccine is the only licensed *M.tb* vaccine and it has been administered globally to several billion people over a 90 year period, by the intradermal route [5]. Although it does not protect against pulmonary TB in endemic areas, it is effective in preventing disseminated TB disease including tuberculous meningitis in childhood [4, 6, 7].

Recent advances in TB vaccine development have primarily been in the area of viral-vectored vaccines, given in prime-boost regimes with BCG as the priming vaccine. The most advanced of these vaccine candidates, MVA85A, had promising phase I results in the UK when given by the intradermal route, but subsequently showed significantly lower immunogenicity in phase II efficacy trials in South Africa [8, 9]. The failure of the MVA85A vaccine to improve efficacy in BCG-vaccinated infants and adults highlights our inability to predict which candidate TB vaccines might work in humans. The predictive value of preclinical animal models remains uncertain, and we do not have a validated immunological correlate of protection with which to guide vaccine design and the selection of which candidate vaccines should progress to efficacy trials.

Historically, the main immunological readout for assessing immunogenicity in TB vaccine trials has been measurement of antigen specific interferon-gamma (IFN-γ) release by T cells in one of several immunological assays [10]. There is considerable evidence to support the use of IFN-γ as an immunological readout [11-13], but whilst IFN-γ is essential for protective immunity against *M.tb*, it may not be sufficient. There are other cytokines, such as tissue necrosis factor alpha (TNFα), which are known to be important, as well as other functions of T cells which may be involved [10, 11].

Challenge models

Currently, there is no reliable alternative to large, randomized controlled trials in order to assess vaccine efficacy against TB. These efficacy trials for novel TB vaccines are challenging, time consuming and very costly [14]. The development of a safe controlled human mycobacterial challenge model which would ultimately be validated against field efficacy studies could greatly facilitate TB vaccine development. Such a model could be used both to guide vaccine selection and facilitate identification and validation of potential immunological correlates of protection.

In vaccine development for other pathogens such as malaria and para-typhoid, where animal models and immunological readouts are of uncertain or limited relevance, such challenge studies have been shown to be of great utility. So far it is has not been possible to consider a human challenge approach in TB vaccine development because humans cannot be safely challenged with wild type virulent *M.tb.* Efforts to develop safe, attenuated strains of *M.tb*, with reporter genes to facilitate sensitive detection of changes in bacterial load, are progressing (Walker et al, personal communication). In the meantime, however, progress with establishing basic parameters surrounding a clinical challenge model can be conducted using the existing TB vaccine, Bacille Calmette-Guérin (BCG), a live attenuated strain of *M. bovis*, which is licensed for human use. Using BCG as a model organism allows us to establish the optimal parameters for a human challenge model, which can then be applied to the challenge reporter strain when it becomes available.

Three studies to date have evaluated BCG as an intradermal challenge agent. In this approach, a skin biopsy is taken two weeks after BCG 'challenge' and the mycobacteria in the biopsy are quantified by culture and PCR [15-17],). This model has demonstrated that viable BCG, detected using culture and PCR, can be recovered from a skin biopsy taken two weeks after BCG challenge. Furthermore we have demonstrated that prior BCG vaccination modulates mycobacterial recovery from subsequent BCG challenge, in a population where BCG has previously been shown to protect against TB [15, 16, 18]. We have also shown that the magnitude of the immune response correlates inversely with mycobacterial growth and have used the challenge model to identify mechanisms responsible for this response [19].

Basis for the aerosol inhaled route

A limitation of the BCG challenge work conducted to date is the intradermal route of delivery. The natural route of infection for *M.tb* is by inhalation of aerosolised infectious droplets containing tubercle bacilli, leading to the establishment of primary infection in the lung. The lung has a distinct mucosal immune system characterized by bronchus associated lymphoid tissue (BALT), which is well adapted to encounter and process antigens such as *M.tb*. Administering BCG challenge via the airway should therefore have the advantage over other routes, of more correctly reflecting the mucosal response to infection with *M.tb,* thus helping us to understand the evolution of early mycobacterial infection in the lung. A successful challenge model by this route will be of great value in testing the efficacy of future vaccines. The inhaled route is a well-established route of drug delivery. Aerosolised droplets of bronchodilating, anti-inflammatory, and antimicrobial drugs are administered by inhalation to millions of patients each day. In the 1960s, BCG was safely delivered by aerosol in a small study in healthy subjects and in two studies in patients with lung cancer [20, 21]. Further clinical studies evaluating the feasibility of an aerosol challenge strategy are needed.

Aerosol delivery devices

Currently the World Health Organisation (WHO) is making a major investment in developing new aerosol devices, providing a portable, low cost method of vaccine delivery [22]. These devices are small, lightweight, portable, and aerosolise through a mesh to provide small and consistent particle size which deliver a vaccine or, for this trial, challenge agent, to the distal airway mucosa more consistently and precisely than conventional jet mechanisms.

One such mesh nebuliser is the MicroAIR NE-U22 (Omron® Healthcare Limited, Japan) which uses ultrasound to push liquid through a fine metal mesh. This generates an aerosol mist with a particle diameter of about 4 µm. It is in current use with licensed drugs such as bronchodilating and antimicrobial agents and achieves good bioavailability. BCG particles are typically around 2-4µm in size and can therefore be aerosolised with minimal damage [23-25]. We have used this device in our previous human aerosol TB vaccine clinical trials, TB026 and TB035, with a good usability and safety profile.

A GLP study assessing the toxicity of BCG administered via the intranasal route has recently completed. Administration of BCG SSI to BABL/c mice by the intranasal route was associated with changes in the lungs and lymph nodes consistent with exposure to the test item and induction of an adaptive immune response. A recent study has demonstrated BCG viability following aerosol delivery to the lungs of rhesus macaques using this nebuliser, with a good safety profile and PPD-specific cellular immune responses in BAL samples [25].

Bronchoscopy and bronchoalveolar sampling

BAL samples enable us to quantify the growth of the challenge agent itself through culture and PCR assessment of BCG CFU counts in the BAL sample. This is of key importance in evaluating this as a viable challenge route for the future assessment of vaccines. BAL samples also provide a relatively easily obtained sample of the lung's immune response to the challenge agent [26]. Protection from TB induced by intra-pulmonary vaccination correlates with cellular immune responses detected in the BAL samples of immunised mice [27]. In macaques receiving aerosolised MVA85A we detected significant levels of antigen-specific cellular immune responses in BAL samples [28], while in TB026, where 12 UK adults received aerosol MVA85A, we detected significant levels of antigen-specific cellular immune responses in these BAL specimens, with higher frequencies in the aerosol group [29]. Both CD4+ and CD8+ antigen specific T cells were detectable in the BAL. In macaques receiving aerosolised BCG, polyfunctional T cells and significant levels of antigen-specific cellular immune responses were detected in BAL specimens [25].

BAL samples are obtained by performing a bronchoscopy, a widely and safely used procedure. The short procedure involves the insertion of a narrow flexible fibre-optic tube into the airway under light intravenous sedation and topical local anaesthesia. Under direct vision, saline is delivered to a section of lung mucosa and then recollected by suction. In clinical trials TB026 and TB035 bronchoscopies have been well tolerated by volunteers.

6.2 Rationale

Hypothesis

We postulate that aerosol inhalation is a practical and feasible route of BCG challenge with an acceptable safety profile in healthy volunteers. We hypothesise that an aerosol BCG challenge will result in the recovery and quantification of BCG through the BAL procedure, and will provide a more physiological model of early mycobacterial infection.

Challenge Agent and dosage

BCG SSI or BCG Bulgaria (InterVax) will be used for this study. From previous studies there is evidence that only 10% of the intradermal dosage is required for mucosal administration (29, 30). With this in mind, and as this trial will be the first GCP clinical trial to give aerosolised BCG to humans, we plan to start at a very low challenge doses with incremental dose increases.

Arm 1 (BCG SSI)

The intradermal licensed dose of BCG SSI is 2-8 x 10^5 cfu (0.1ml)

The doses for the groups in Arm 1 will be on the basis of a median BCG concentration of 5 x 10^6 cfu/ml. Group 1A will receive 1 x 10^3 cfu BCG SSI by aerosol inhalation. The first volunteer in this group will be challenged ahead of other volunteers and no other volunteers will be challenged until at least 7 days has elapsed. The CI will perform a review of the 7 day safety data and decide if it is safe to challenge the remaining volunteers. Further details of safety reviews are given in section 11.1.

There will be a second review of safety data after challenge of all volunteers in Group 1A.

We will then dose escalate to 1 x 10^4 cfu BCG SSI for Group 1B in the same manner, with a safety review 7 days after challenge of the first volunteer. There will be a second review of safety data after challenge of all volunteers in Group 1B.

Once Group 1B is fully enrolled and the second safety review is completed, we will start randomisation to Groups 1C and 1D, with dose escalation to 1 x 10^5 cfu. For safety reasons, the first volunteer will be assigned to Group 1C (aerosol BCG SSI plus ID saline placebo) and there will be no further challenges until 7 days have elapsed and the safety data reviewed. If the CI decides it is safe to proceed, the remaining volunteers will then be randomised and challenged.

Arm 2 (BCG Bulgaria (InterVax))

The intradermal licensed dose of BCG Bulgaria (InterVax) is 1.5-6.0 x 10^5 cfu (0.1ml).

The doses for the groups in Arm 2 will be on the basis of an average BCG concentration of 4 x 10^6 cfu/ml. Group 2A will receive 1 x 10^4 cfu BCG Bulgaria (InterVax) by aerosol inhalation. The first volunteer in this group will be challenged ahead of other volunteers and no other volunteers will be challenged until at least 7 days have elapsed. The CI will perform a review of the 7 day safety data and decide if it is safe to challenge the remaining volunteers. Further details of safety reviews are given in section 11.1.

There will be a second review of safety data after challenge of all volunteers in Group 2A.

We will then dose escalate to 1 x 10^5 cfu BCG Bulgaria (InterVax) for Group 2B in the same manner, with a safety review 7 days after challenge of the first volunteer. There will be a second review of safety data after challenge of all volunteers in Group 2B.

We will then dose escalate to 1 x 10^6 cfu BCG Bulgaria (InterVax) for Group 2C, with a safety review 7 days after challenge of the first volunteer. There will be a second review of safety data after challenge of all volunteers in Group 2C.

We will then dose escalate to 1 x 10^7 cfu BCG Bulgaria (InterVax) for Group 2D, with a safety review 7 days after challenge of the first volunteer. There will be a second review of safety data after challenge of all volunteers in Group 2D.

Once Group 2D is fully enrolled and the fourth safety review is completed, we will start randomisation to Groups 2E and 2F. The dose used for challenge of the volunteers in Group 2E will be either 1 x 10^6 cfu or 1 x 10^7 cfu, depending on which dose shows the most promising results from Groups 2C and 2D. The dose used for intradermal challenge in Group 2F will be a log lower than Group 2E.

The rationale for dose escalating to 1 x 10^6 cfu was based on the following:

1. We will carefully dose escalate according to the schedule above and will not dose escalate beyond a dose that is not tolerated. If the 10^5 cfu dose is not well tolerated, we will not dose escalate further.
2. The amount of viable BCG cfu recoverable from a vial of BCG is always lower than the expected amount based on the SMPC. Therefore we anticipate dosing using the SMPC with a dose of 10^6 cfu will result in a delivered dose that is less than 10^6 cfu. The licensed dose of BCG SSI is 2-8 x 10^5 cfu.
3. In a previous BCG study we have administered 3 x the standard dose of BCG by intradermal injection, and this was well tolerated [17]. This was a non-CTIMP challenge study (TB031) where 10 participants received BCG SSI intradermally at a dose range of 5 x 10^5 to 2.4 x 10^6 cfu. As this higher dose of BCG SSI was previously shown to be well tolerated (no SAEs, with local and systemic AEs being mostly mild in nature), we would anticipate a similar safety profile in TB041 where participants will receive 1 x 10^6 cfu of the BCG Bulgaria strain. Furthermore, an additional 10 participants received an intradermal immunization with BCG Tice at a dose range of 5 x 10^5 to 2.4 x 10^6 cfu. This was also well tolerated with local and systemic AEs being mostly mild in nature.
4. To date in this study, administering a dose of 1 x 10^5 cfu has been well tolerated. Furthermore, the immunogenicity data on this dose demonstrates a relatively low immunogenicity compared with both the
5.

Figure 1: PPD ELISpot responses after BCG SSI 10^5 cfu delivered by either aerosol or intradermal route, compared with a prior clinical study with BCG SSI (TB038) where the dose was 100µl.

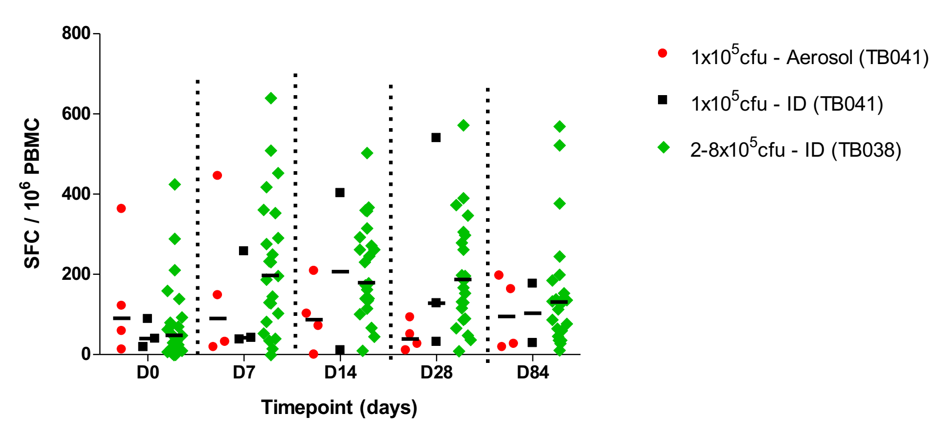

The rationale for further dose escalating to 1 x 10^7 cfu (for aerosol delivery only) is based on the following:

1. The safety data for all volunteers dosed to date has been reviewed and there have been no safety concerns in any volunteer. This includes data from all 3 Group 2C volunteers up to Day 14 who received a dose of 1x 10^6 cfu via aerosol. This dose was well tolerated. The progress to date of volunteers is shown below (green indicates visit completion).

Group	Vol no.	D0 Challenge	D2	D7	D14 Bronch	D28	D84	D168
2A	TBT-0411017	12/04/2017	13/04/2017	19/04/2017	26/04/2017	10/05/2017	05/07/2017	27/09/2017
2A	TBT-0411013	19/04/2017	20/04/2017	26/04/2017	03/05/2017	17/05/2017	10/07/2017	11/10/2017
2A	TBT-0411019	01/06/2017	02/06/2017	07/06/2017	22/06/2017	29/06/2017	06/09/2017	16/11/2017
2B	TBT-0411021	14/06/2017	16/06/2017	21/06/2017	29/06/2017	10/07/2017	12/09/2017	29/11/2017
2B	TBT-0411023	09/08/2017	11/08/2017	15/08/2017	24/08/2017	06/09/2017	01/11/2017	22/01/2017
2B	TBT-0411024	09/08/2017	11/08/2017	15/08/2017	24/08/2017	13/09/2017	01/11/2017	22/01/2017
2C	TBT-0411025	16/08/2017	17/08/2017	23/08/2017	30/08/2017	12/09/2017	07/11/2017	30/01/2018
2C	TBT-0411029	20/09/2017	22/09/2017	28/09/2017	04/10/2017	18/10/2017	13/12/2017	07/03/2017
2C	TBT-0411031	20/09/2017	22/09/2017	27/09/2017	04/10/2017	18/10/2017	13/12/2017	07/03/2017

2. The amount of viable BCG cfu recoverable from a vial of BCG is always lower than the expected amount based on the SmPC. The viable BCG cfu from the vials of BCG Bulgaria reconstituted in the trial to date is shown below, Figure 2. It is consistently half to 1 log lower than the 1.5-6 x 10^5/ml cfu stated in the SmPC.

Figure 2: BCG dose loaded into nebuliser following BCG reconstitution.

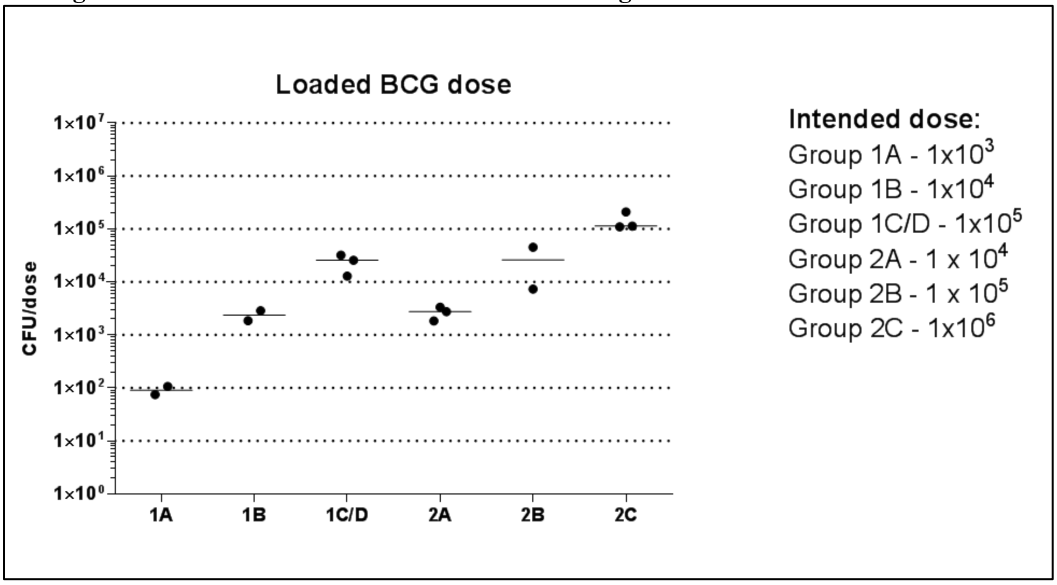

3. We have developed a model to help quantify the actual delivered dose of nebulised BCG through the nebuliser. This involved using a vacuum pump attached to an impinger to collect the aerosol expelled by the Omron nebulizer. The amount of BCG in this collected sample was quantified by plating onto solid agar. This model consistently measured an approximately 50% loss of BCG compared with the amount of BCG loaded into the nebuliser. See Figure 3 below. Of importance, this model does not take into account the further certain significant loss of BCG that occurs in a clinical setting through loss of aerosol into the environment, as the volunteer does not breathe in continuously, as with our vacuum model, and the nebuliser continues to aerosolise the BCG between breaths.

Figure 3: BCG recovery after nebulisation using vacuum model

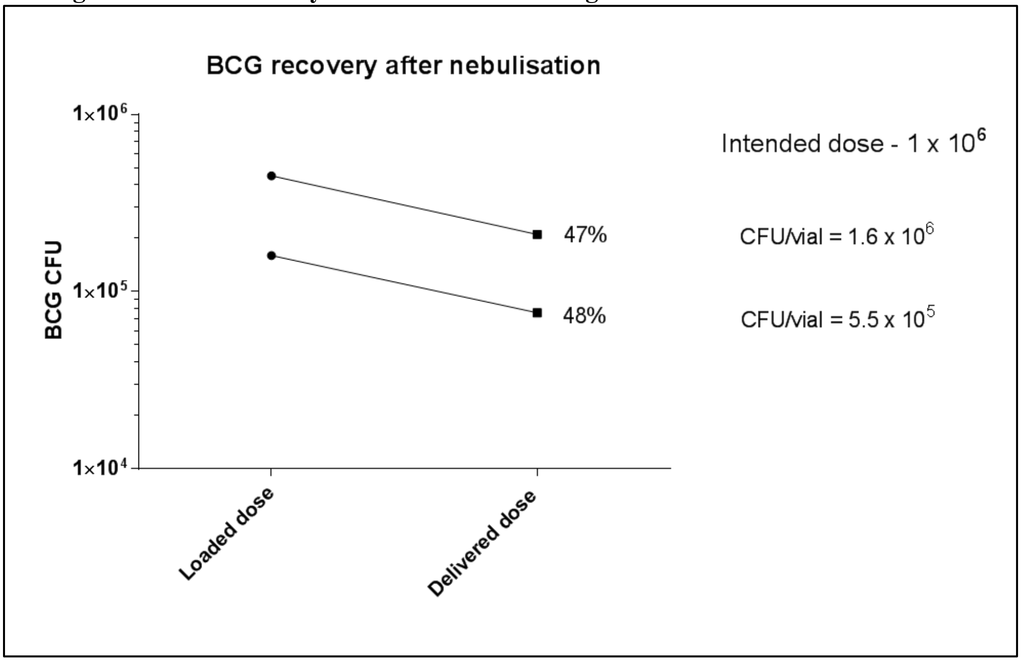

Our target continues to be to deliver the standard licensed BCG Bulgaria dose of approximately 4 x 10^5 BCG cfu via aerosol into the airway of the volunteer (if safety allows) and to compare this with the same dose delivered intradermally. As outlined above, the amount of viable units in the vials is consistently a half to one log lower, justifying our previous rationale for a dose escalation to 1 x 10^6 BCG cfu.

However, we have now further demonstrated that there is at least another 50% to one log loss of BCG in the process of nebulisation. Hence in order to account for these losses and achieve the actual delivered dose of

approximately 4 x 10^5 cfu into the airways of the volunteer, we would need to load the nebuliser cup with 1 x 10^7 cfu ("prepared" dose).
Given the above, we need to ensure that we are delivering a comparable dose of BCG via the two routes (ID or aerosol). Therefore to account for the loss that occurs during nebulisation we plan to load the nebuliser cup (Group 2E) with a dose 1 log higher to the amount loaded into the syringe for intradermal injection (group 2F). The prepared dose of Group 2E will depend on safety data from Groups 2C and 2D, as previously.

6.3 Risks and Benefits

Potential risks

The potential risks to participants in this trial include risks associated with:

1. Venepuncture and intravenous cannulation

Localised bruising and discomfort can occur at the site of venepuncture. Infrequently fainting may occur. The total volume of blood drawn over a six month period will be 422mL (blood volumes for volunteers outside Oxford may be slightly lower or higher than those for Oxford volunteers due to use of different volume vacutainers, following local Trust SOPs), which should not compromise these otherwise healthy volunteers, as they would donate 470mL during a single blood donation for the National Blood transfusion Service over a 3-4 month period. Volunteers will be asked to refrain from blood donation for the duration of their involvement in the trial.
An intravenous cannula is routinely inserted into a peripheral (usually forearm) vein prior to bronchoscopy in order to administer intravenous sedation. This is removed once the procedure is completed. The risks of cannulation are identical to those associated with venepuncture but include an additional small risk of soft tissue infection. This risk will be minimised by an aseptic insertion technique and is easily recognisable and treatable. The short duration of cannulation (a few hours) further minimizes this risk.

2. BCG challenge

BCG SSI is a licensed vaccine that has been given to over 3 billion people throughout the world since it was first developed in 1921. BCG Bulgaria (InterVax) is on the WHO list of pre-qualified vaccines and has a well-defined side effect profile that is similar to that of BCG SSI. Full details are given in the SmPCs. BCG is licensed for delivery via the intradermal route. It is not licensed for delivery via the aerosol route. The potential known adverse events are:

Local reaction from ID administration (Group 1D and 2F)

An inflammatory reaction as manifested by redness and swelling is expected to occur at the site of injection, followed by a local lesion that may ulcerate. This heals over some weeks to months to leave a small flat scar. It is also possible to develop some swelling (<1cm diameter) of axillary lymph nodes.
Uncommonly (less than 1 in 100 people) swelling of axillary lymph nodes to more than 1cm across, or an ulcer that discharges fluid at the injection site may occur.
Rare side effects (less than 1 in 1000 people) include inflammation of lymph nodes leading to abscesses and discharge of fluid from the swellings. Such a reaction would require treatment with antibiotics.

Local reaction from aerosol inhaled challenge (Groups 1A, 1B, 1C, 2A, 2B, 2C, 2D and 2E)

Expected reactions to aerosol challenge include mild throat discomfort and coughing. Transient bronchospasm (causing wheeze) is possible. As this is a novel route for BCG there are no other expected reactions.

Systemic reactions

Systemic reactions to intradermal BCG are rare (less than 1 in 100 people) and include low-grade fever and headache, and allergic reactions. In a previous trial of aerosol BCG around 1/3 of participants experienced fever and malaise. Disseminated complications of BCG, such as bone infections, have also been reported, but are extremely rare and have usually been reported in immunocompromised, not immunocompetent, individuals.

Allergic reactions

Allergic reactions from mild to severe may occur in response to any constituent of a medicinal product's preparation. Anaphylaxis is extremely rare (less than 1 in 1000 people) but can occur in response to any vaccine or medication.

3. Bronchoscopy

Bronchoscopy is a widely and safely used investigative procedure in clinical research studies involving both healthy volunteers and patients with respiratory conditions such as asthma and interstitial lung disease [30, 31]. Clinical guidelines for performing investigative bronchoscopy in research studies are well established [32]. The bronchoscopies will be carried out in a dedicated NHS bronchoscopy suite with an excellent safety record by highly skilled and experienced consultant respiratory physicians. Intravenous sedation and topical local anaesthesia are administered prior to bronchoscopy to reduce discomfort, facilitate the procedure, and remove memory of the event (In 98% of cases, subjects have no memory of the procedure). Trained, experienced staff and facilities for resuscitation and drugs for reversal of sedation will be available. No transbronchial or endobronchial biopsies will be taken. To further minimise risk, volunteers will be excluded if they have an

abnormal chest radiograph, a significant smoking history, a history of atopy or any evidence of lung disease, including asthma (as defined by: a clinical diagnosis of asthma; prescription of asthma medication; airflow obstruction on spirometry; history of nocturnal or exercise-induced wheeze).
The risks of bronchoscopy are discussed at the time of consent and comprise: adverse reaction to sedation or local anaesthetic, sore throat and /or transient hoarse voice, laryngospasm/bronchospasm, hypoxia, post-procedure flu-like symptoms (1-2 days) and risk of death (<1 in 100,000).

- Bronchoalveolar lavage is a routine procedure in investigative bronchoscopy and carries a minimum bleeding risk. The risk of infection or febrile reactions will be minimised by full bronchoscope asepsis and bronchoscope superflush.
- Respiratory depression secondary to sedation is rare. No rescue medication for oversedation has been required for volunteers participating in our aerosol clinical trials (TB026 and TB035) to date.
- Allergic reactions from mild to severe may occur in response to any constituent of the local anaesthetic or sedative agents. Anaphylaxis is extremely rare but can occur.

The Summary of Product Characteristics (SmPC) for the local anaesthetic and sedative agents contain full details of the indications and side effects of these licensed medications.

***4. Punch biopsy** (Group 1D and 2F only)*

- There is a small risk that the punch biopsy site may become infected. If this did occur, treatment with antibiotics may be required. The punch biopsy site will heal to form a small scar. Allergic reactions from mild to severe may occur in response to any constituent of the local anaesthetic or sedative agents. Anaphylaxis is extremely rare but can occur.

5. Chest radiograph

A chest radiograph is a painless radiological investigation which exposes volunteers to approximately 0.02 milliSieverts of radiation, equivalent to around 3 days of natural background radiation. The additional risk of cancer due to one chest radiograph is insignificant (1 in 900,000).

6. Spirometry

Vigorous respiratory manoeuvres such as forced expiration through the spirometer can occasionally lead to coughing or light-headedness, but these symptoms are mild and rapidly self-limiting.

***7. Induced sputum** (Groups 2A-2E)*

Inhalation of hypertonic saline to induce sputum production can occasionally result in excessive bouts of coughing or sensation of shortness of breath but these symptoms are mild and rapidly self-limiting. Rarely, hypertonic saline can induce bronchoconstriction but this is very unlikely to occur in our cohort of patients who have no history of atopy or asthma. Hypertonic saline-induced bronchospasm is quickly reversed by treatment with an inhaled short-acting B_2 agonist. Volunteers will be pre-treated with nebulised salbutamol and their lung function will be monitored throughout the procedure.

Potential benefits

Volunteers are not expected to benefit directly from participation in this trial. Volunteers will gain some information about their general health as a result of the screening history, examination, blood tests, urine tests, chest radiograph and spirometry. They may also gain health information from the bronchoscopy. Volunteers found to have a previously undiagnosed condition thought to require further medical attention will be referred appropriately to their GP or an NHS specialist service for further investigation and treatment, with their permission.
It is hoped that their contribution will further the development of a safe and successful vaccine for TB and our knowledge about TB infection and protection.

7 OBJECTIVES AND OUTCOME MEASURES

**Objectives**	**Outcome Measures**	**Timepoint(s) of evaluation of this outcome measure**
Primary Objective<br>To evaluate the safety of BCG challenge by the aerosol inhaled route in healthy volunteers	Actively and passively collected data on adverse events	At each visit and via e-diary for 28 days after challenge
**Secondary Objective**<br>To evaluate and compare the amount of BCG recovered from BAL in healthy BCG-naïve adults receiving both aerosol inhaled and ID BCG and from punch biopsy in adults receiving ID BCG	Culture and PCR quantification of BCG in BAL and punch biopsy	Day 14

**Tertiary Objective**<br>To identify laboratory markers of the immune response that correlate with the levels of BCG recovered at the challenge site	Established and exploratory markers of innate, cell mediated and humoral immunity in blood and BAL samples	At each visit
**Quaternary Objective**<br>To evaluate and compare the systemic and mucosal cellular and humoral immunogenicity induced by BCG challenge by the aerosol inhaled and ID route, in healthy volunteers	Laboratory markers of cell mediated and humoral immunity, including *ex-vivo* ELISpot in blood and intracellular cytokine staining in blood and BAL samples	At each visit

Table 2. Objectives and Outcome Measures

8 PARTICIPANT IDENTIFICATION

8.4 Trial Participants

Healthy, BCG-naïve UK adults have been chosen because of their low baseline level of anti-mycobacterial immunity and therefore reduced ability to suppress BCG growth. This maximizes the chance of detecting BCG at the challenge site.

8.5 Inclusion Criteria

Volunteers must meet all of the following criteria to enter the trial:

- Healthy adult aged 18-50 years
- Resident in or near Oxford (CCVTM or OUH) or Birmingham (NIHR-WTCRF) for the duration of the trial period
- Screening IGRA negative
- Chest radiograph normal
- No relevant findings in medical history or on physical examination
- Allow the Investigators to discuss the individual's medical history with their GP
- Use effective contraception (see below) for the duration of the trial period (females only)
- Refrain from blood donation during the trial
- Give written informed consent
- Allow the Investigator to register volunteer details with a confidential database (The Over-volunteering Protection Service) to prevent concurrent entry into clinical studies/trials
- Able and willing (in the Investigator's opinion) to comply with all the trial requirements

8.6 Exclusion Criteria

Volunteers must meet none of the following criteria to enter the trial:

- Previously resident for more than 12 months concurrently in a tropical climate where significant non-tuberculous mycobacterial exposure is likely
- Participation in another research trial involving receipt of an investigational product in the 30 days preceding enrolment, or planned use during the trial period[a]
- Prior vaccination with BCG or any candidate TB vaccine
- Administration of immunoglobulins and/or any blood products within the three months preceding the planned trial challenge date
- Clinically significant history of skin disorder, allergy, atopy, immunodeficiency (including HIV), cancer (except BCC or CIS), cardiovascular disease, gastrointestinal disease, liver disease, renal disease, endocrine disorder, neurological illness, psychiatric disorder, drug or alcohol abuse
- Concurrent oral or systemic steroid medication or the concurrent use of other immunosuppressive agents
- History of anaphylaxis to vaccination or any allergy likely to be exacerbated by any component of the trial agent, sedative drugs, or any local or general anaesthetic agents

- Pregnancy, lactation or intention to become pregnant during trial period
- Any respiratory disease, including asthma
- Current smoker
- Clinically significant abnormality on screening chest radiograph[b]
- Clinically significant abnormality of spirometry
- Any nasal, pharyngeal, or laryngeal finding which precludes bronchoscopy
- Current use of any medication taken through the nasal or inhaled route including cocaine or other recreational drugs
- Clinical, radiological, or laboratory evidence of current active TB disease
- Past treatment for TB disease
- Any clinically significant abnormality of screening blood or urine tests[b]
- Positive HBsAg, HCV or HIV antibodies[b]
- Any other significant disease, disorder, or finding, which, in the opinion of the Investigator, may either put the volunteer at risk, affect the volunteer's ability to participate in the trial or impair interpretation of the trial data

[a] Volunteers will be excluded from the trial if they are concurrently involved in another study or trial that involves regular blood tests or an investigational medicinal product. In order to check this, volunteers will be asked to provide their National Insurance or Passport number (if they are not entitled to a NI number) and will be registered on a national database of participants in clinical trials (www.tops.org.uk).
[b] Volunteers who are excluded from the trial because they have been discovered during screening procedures to be suffering from a previously undiagnosed condition thought to require further medical attention will be referred appropriately to their GP or an NHS specialist service for further investigation and treatment.

Effective contraception for female volunteers

Female volunteers are required to use an effective form of contraception during the course of the trial. Although no harmful effects on the foetus have been observed from BCG during pregnancy, it is wise to avoid vaccination during pregnancy, particularly during the first trimester.

Acceptable forms of contraception for female volunteers include:

- Established use of oral, injected on implanted hormonal methods of contraception
- Placement of an intrauterine device (IUD) or intrauterine system (IUS)
- Permanent sterilisation or bilateral tubal occlusion
- Barrier methods of contraception (condom; or occlusive cap with spermicide)
- Male sterilisation, if the vasectomised partner is the sole partner for the subject
- True abstinence, when this is in line with the preferred and usual lifestyle of the subject (periodic abstinence and withdrawal are not acceptable methods of contraception)

9 TRIAL DESIGN AND PROCEDURES

This is a randomised controlled clinical challenge trial of BCG administered by the aerosol inhaled route compared to the intradermal route in healthy UK volunteers.

9.7 Trial Numbers and Groups

For Arm 1 30 volunteers will be enrolled; the first three to Group 1A, the next three to Group 1B, then 24 randomly allocated to either group 1C or group 1D (the 1st volunteer will be in Group 1C).

[Arm 1 of this trial enrolled 13 volunteers before the BCG SSI expired in August 2016. Due to a global shortage of the vaccine, there is no way of knowing when more BCG SSI can be obtained. Recruitment for Arm 1 was therefore stopped in August 2016. There were no safety concerns for any of the groups.]

For Arm 2, 33 volunteers will be enrolled; the first three to Group 2A, the next three to Group 2B, the next three to Group 2C and the next three to Group 2D. Then 21 volunteers will randomly be allocated to either Group 2E (9 volunteers) or Group 2F (12 volunteers).

Arm 1

Group	Sample size	Intervention
Group 1A	3	Aerosol inhaled BCG SSI 1 x 10^3 cfu then bronchoscopy
Group 1B	3	Aerosol inhaled BCG SSI 1 x 10^4 cfu then bronchoscopy
Group 1C	12	Aerosol inhaled BCG SSI 1 x 10^5 cfu and intradermal saline placebo then bronchoscopy
Group 1D	12	Aerosol inhaled saline placebo and intradermal BCG SSI 1 x 10^5 cfu then bronchoscopy and punch biopsy
Arm 2		
Group	Sample size	Intervention
Group 2A	3	Aerosol inhaled BCG Bulgaria (InterVax) 1 x 10^4 cfu then bronchoscopy
Group 2B	3	Aerosol inhaled BCG Bulgaria (InterVax) 1 x 10^5 cfu then bronchoscopy
Group 2C	3	Aerosol inhaled BCG Bulgaria (InterVax) 1 x 10^6 cfu then bronchoscopy
Group 2D	3	Aerosol inhaled BCG Bulgaria (InterVax) 1 x 10^7 cfu then bronchoscopy
Group 2E	9	Aerosol inhaled BCG Bulgaria (InterVax) either 1 x 10^6 or 1 x 10^7 cfu and intradermal saline placebo then bronchoscopy. Dosing will depend on the optimal dose identified from results obtained from Groups 2C and 2D
Group 2F	12	Aerosol inhaled saline placebo and intradermal BCG Bulgaria (InterVax), at a dose a log lower than 2E ie either 1 x 10^5 or 1 x 10^6 cfu, then bronchoscopy and punch biopsy

Table 3. Trial groups

9.8 Recruitment

Volunteers may be recruited by use of an advertisement formally approved by the ethics committee and distributed or posted in the following places:

- In public places (including NHS hospitals and university buildings) with the agreement of the owner or proprietor
- In newspapers or other literature for circulation
- On radio via announcements
- On a website operated by our group or with the agreement of the owner or operator
- By e-mail distribution to a group or list only with the express agreement of the network administrator or with equivalent authorisation
- On stalls or stands at exhibitions or fairs
- Direct mail-out: this will involve obtaining names and addresses of adults via the most recent Electoral Roll. The contact details of individuals who have indicated that they do not wish to receive postal mail-shots would be removed prior to the Investigators being given this information. The company providing this service is registered under the Data Protection Act 1998. Investigators would not be given dates of birth or ages of individuals, but the list supplied would only contain names of those aged between 18-50 years (as per the inclusion criteria).
- Oxford Vaccine Centre databases: we may contact individuals from databases of groups within the CCVTM (including the Oxford Vaccine Centre database) of previous trial participants who have expressed an interest in receiving information about future studies for which they may be eligible.

A copy of intended advertising will be submitted with the initial application for ethical approval and any significant changes to this advertisement will be submitted as an amendment to the ethics committee for approval before use.
Volunteers who express an interest in the trial will be given a Participant Information Sheet and asked to register for the trial through the website. Their details will be passed to the trial team to arrange a pre-screening call and screening appointment.

9.9 Informed Consent

Written informed consent will be obtained at screening by a GCP trained Investigator.
The volunteer must sign and date the latest approved version of the consent form before any trial specific procedures are performed.

The Participant Information Sheet will be made available to the volunteer no less than 24 hours prior to attending for screening. The details enclosed will be discussed with the volunteer, including:

- the aims and nature of the trial
- the agents used
- the schedule of visits and tests to be carried out
- the implications and constraints of the protocol
- compensation for the volunteer
- known side effects and risks of taking part in the trial

The following general principles will be emphasised:

- Participation is entirely voluntary
- The volunteer may withdraw from the trial at any time for any reason
- Withdrawal or refusal to participate involves no penalty or loss of medical benefits
- The volunteer is free to ask questions at any time to allow him or her to understand the purpose of the trial and the procedures involved
- There is no direct benefit from participating
- The volunteer's GP will be contacted to corroborate their medical history and confirm that the volunteer does not meet any of the exclusion criteria
- The volunteer will be registered on the TOPS database (The Over-Volunteering Protection System)
- The volunteer's blood samples taken as part of the trial will be stored indefinitely for further research. The volunteer may elect to have their samples destroyed after the period required to meet Good Clinical Practice and regulatory requirements.

The volunteer will then have time to consider whether or not to participate. If the volunteer decides to participate, they will sign and date two copies of the consent form, one for them to take away and keep, and one for the Investigator which will be retained at the trial site. These forms will also be signed and dated by the Investigator. This will occur before any trial specific procedures are performed.

9.10Screening and Eligibility Assessment

Once the volunteer has given their consent to undergo screening, a baseline medical history (including concomitant medication) and physical examination will be performed by a GCP trained doctor. Inclusion and exclusion criteria will be checked using a tabulated format. Demographic and occupational data will be collected. Vital signs will be checked and bloods taken including FBC, clotting profile, U&Es, LFTs, HIV antibodies, HBsAg, HCV antibodies and IGRA. Spirometry will be performed according to local SOP. Volunteers will be counselled by one of the Investigators for HIV, Hepatitis B and Hepatitis C testing. Urine will be tested for the presence of clinically significant proteinuria, glycosuria or haematuria. A pregnancy test will be performed for female volunteers. Volunteers will then attend for a chest radiograph.
Laboratory parameters for inclusion/exclusion in the trial will be considered on an individual basis, with Investigator discretion for interpretation of results and the need for repeated tests. In general, volunteers will be excluded if a result at screening constitutes what would qualify as a grade 1 (or higher) laboratory AE, according to the site-specific laboratory AE tables found in the relevant SOP and filed in the TMF (Oxford), or Site Master File (Birmingham).
The total duration of the screening visit will be around 2 hours.

9.11Randomisation and blinding

To reduce bias and the influence of any difference in the baseline characteristics between Groups 1C and 1D (Arm 1), as well as Groups 2E and 2F (Arm 2), we will randomise the allocation of volunteers by variable block randomisation using sequentially numbered sealed envelopes, prepared by an independent colleague at the Centre for Statistics in Medicine. This aims to achieve a balance between the two groups in any important confounding factors such as levels of pre-existing anti-mycobacterial immunity (influenced by, for example, country of origin). Randomisation will occur for Groups 1C and 1D (Arm 1), as well as Groups 2E and 2F (Arm 2), only (for Arm 1, the first volunteer will be assigned to Group 1C for safety).
Volunteers in randomised groups will be blinded to eliminate subject bias (either conscious or subconscious) using a paired placebo design, whereby volunteers randomised to groups 1C and 2E (inhaled aerosol) will receive a concurrent intradermal saline injection, while volunteers in groups 1D and 2F (intradermal) will receive a concurrent dose of inhaled saline. This design has the added benefit of allowing a distinction to be made between any adverse events attributable to the method (including nebuliser device) of challenge delivery and those attributable to the inhaled challenge product itself. At day 14 these volunteers will be unblinded as Groups 1D and 2F will undergo punch biopsy whereas Groups 1C and 2E will not. It would not be ethical to

perform punch biopsy on Groups 1C and 2E volunteers to maintain blinding. There is potential impact on the reporting of AEs from this period hence, but as we anticipate the bulk of challenge-related AEs occurring in the first 2 weeks post challenge, this is a reasonable compromise in order to not subject these volunteers to an unnecessary procedure. A volunteer in Group 2E may become unblinded prior to day 14 if their induced sputum procedure falls at day 2 or day 7 follow up, but this will be in the minority of cases given not all Group 2E volunteers will be having induced sputum scheduled at these visits and not at all if they are enrolled at Birmingham.

The bronchoscopist performing the procedure at day 14 will be blinded to the route of challenge administration to eliminate any bias in the reporting of the appearance of the lung mucosa and extent of airway inflammation. The laboratory staff analysing the immunology samples will be blinded to reduce any bias that could be introduced at the sample processing stage by anonymising all samples.

9.12Follow up visits

D0 - Challenge

Any new medical issues or symptoms that have arisen will be assessed. Venepuncture and other samples will be taken according to the Schedule of Trial Procedures in section 4.2. Spirometry will be performed. The inclusion and exclusion criteria for the trial will be reviewed; provided that the volunteer still satisfies all inclusion criteria (and no exclusion criteria) and their consent remains valid, the volunteer will be allocated to a group. BCG challenge will be performed by the aerosol or intradermal route according to the site specific SOP. Placebo challenge will also be performed, according to their group. Volunteers will be kept under observation for around 60 minutes after challenge. The challenge visit will last approximately 2 hours. The e-diary will be set up during this visit. Birmingham volunteers will have transport to and from Oxford arranged for them.

D14 – Bronchoscopy

The bronchoscopy visit lasts several hours and takes place at the bronchoscopy suite in the OUH NHS Foundation Trust by the specialist team there. Blood will be taken as specified in the Schedule of Trial Procedures (section 4.2). Details of the procedure are outlined in section 6.1. Birmingham volunteers will have transport to and from Oxford arranged for them.

D14 – Biopsy (Groups 1D and 2F)

A 4mm x 3mm punch biopsy will be performed over the intradermal challenge site with local anaesthetic. This will be performed with a standard punch biopsy needle as used in outpatient dermatology clinics. This size is ideal for sampling the majority of the inflamed skin at the BCG challenge site. This procedure will occur following bronchoscopy and will add approximately 15 minutes to the visit.

Other follow-up visits

These will be performed as specified in the Schedule of Trial Procedures (section 4.2). Any new medical issues or symptoms that have arisen will be assessed, and the e-diary and any ongoing adverse events will be reviewed. Additional procedures or laboratory tests may be carried out at the discretion of the Investigators if deemed clinically necessary. Follow-up visits will last approximately 15-30 minutes, except for those having induced sputum where the visit will last approximately 1.5 hours. The maximum trial duration will be around 24 weeks after the date of enrolment.

Induced sputum collection will occur at 3 of the 5 follow up visits for Oxford volunteers in Groups 2A-2E. In order to collect samples at the maximum number of time points without overly burdening staff and volunteers with this time intensive procedure we are allocating volunteers to one of two schedules. Volunteers in Groups 2A-D will have induced sputum collection at the 4 week, 3 month and 6 month visit. The first 3 volunteers enrolled into Group 2E will also be allocated to the above the schedule. The final 6 volunteers enrolled into Group 2E will be allocated to sputum collection at day 2, day 7 and the 6 month follow up visit. This will ensure that 6 volunteers dosed with the maximal BCG aerosol dose will be allocated to the two schedules. Volunteers from Birmingham, while allocated to a schedule will not actually have the sputum collected due to time and logistic constraints. Induced sputum collection is an experimental aspect of this trial and we have devised the above schedule based on pragmatics and practicality given the limited resources of the clinic.

After the final trial visit, an End of Study letter will be sent to each volunteer's GP to inform them that the volunteer has either completed the trial and which intervention they received, or was not enrolled.

9.13Timepoints

Screening – trial enrolment time window

Enrolment should take place no longer than 120 days following the date of screening appointment. If more than 120 days elapse, the screening visit should be repeated in full prior to enrolment in order to minimise the risk to participants of any new unidentified health problems having arisen during that period.

Challenge of first volunteer – subsequent challenge time window

Arm 1: The first volunteer, who will be allocated to Group 1A, will be challenged with BCG SSI by the aerosol inhaled route at least 7 days before any subsequent volunteers in this group. After safety review of the 3 Group 1A volunteers, the first volunteer in Group 1B will be challenged at least 7 days prior to further volunteer enrolment into Group 1B. After safety review of the 3 Group 1B volunteers, the first volunteer in Group 1C will be challenged at least 7 days prior to further volunteer enrolment into Groups 1C & 1D.
Arm 2: The first volunteer, who will be allocated to Group 2A, will be challenged with BCG Bulgaria (InterVax) by the aerosol inhaled route at least 7 days before any subsequent volunteers in this group. After safety review by the clinical team and the Chief Investigator of the 3 Group 2A volunteers, the first volunteer in Group 2B will be challenged at least 7 days prior to further volunteer enrolment into Group 2B. After similar safety review of the 3 Group 2B volunteers, the first volunteer in Group 2C will be challenged at least 7 days prior to further enrolment into Group 2C. After similar safety review of the 3 Group 2C volunteers, the first volunteer in Group 2D will be challenged at least 7 days prior to further enrolment into Group 2D. After similar safety review of the 3 Group 2D volunteers, and review of the results from Groups 2C and 2D to decide the optimal dose to take forwards, volunteers will be enrolled into Groups 2E and 2F.
Challenge – bronchoscopy and biopsy time window
In previous intradermal BCG challenge studies, two weeks has been established as the optimal time window for BCG recovery; this should also be the case for aerosol challenge.
Follow up period
The follow up period will be six months in accordance with findings from previous studies in which adequate safety data and reliable markers of immunogenicity have been obtained in this time interval.

9.14Sample handling

Details regarding samples, volume and frequency of sampling are listed in the Schedule of Trial Procedures (section 4.2). Blood and other samples will be processed according to local laboratory SOPs. All samples will be in anonymised form at the CCVTM/OUH; samples at the NIHR-WTCRF may contain patient numbers and details as per NHS procedures.
Volunteers will be informed that there may be leftover samples of their blood, BAL, skin biopsies and sputum. With the volunteers' informed consent, any leftover cells and serum, plasma, sputum or BAL samples will be frozen for future analysis of *M.tb* and/or BCG-related responses. This may include human DNA and RNA analysis to search for correlates of TB risk and/or protection. Samples may be shipped to other parties involved in our research in anonymised form for immunological analysis. Volunteers will be able to decide if they will permit such future use of any leftover samples. If they elect not to permit this, all of those leftover samples will be discarded after the required period of storage to meet GCP and regulatory requirements.

9.15Challenge Postponement Criteria

Challenge will not proceed on the scheduled day in any of the following situations:

- The volunteer has a current or recent upper respiratory tract infection, unless they have been symptom-free for at least one week
- The volunteer has a temperature > 37.5^{o}C
- The Investigator judges the volunteer to have an acute moderate or severe illness (whether febrile or not)
- The volunteer has received a live non-trial vaccine within the preceding 28 days
- The Investigator has any other concern that challenge may not be in the volunteer's best interests

In this case the volunteer may be challenged at a later date or withdrawn from the trial at the discretion of the Investigator.

9.16Bronchoscopy Postponement criteria

Bronchoscopy will not proceed on the scheduled day in any of the following situations:

- The volunteer has a temperature > 37.5^{o}C
- The Investigator judges the volunteer to have an acute moderate or severe illness (whether febrile or not)
- The Investigator has any other concern that bronchoscopy may not be in the volunteer's best interests

In the event of postponement the bronchoscopy will be rescheduled when possible up to a 3 week window.

9.17Discontinuation / Withdrawal Criteria

Every reasonable effort will be made to maintain protocol compliance and participation in the trial. In accordance with the principles of the current revision of the Declaration of Helsinki and any other applicable regulations, a volunteer has the right to withdraw from the trial at any time and for any reason, and is not obliged to give his or her reasons for doing so. The Investigator may withdraw the volunteer at any time in the interests of the volunteer's health and well-being (including on the advice of the LSC). In addition the volunteer may withdraw/be withdrawn for any of the following reasons:

- Administrative decision by the Investigator
- Ineligibility (either arising during the trial or retrospectively, having been overlooked at screening)
- Significant protocol deviation
- Volunteer non-compliance with trial requirements (failure to attend two or more follow-up visits)
- Any AE which requires discontinuation of trial involvement or results in inability to comply with trial procedures
- Confirmed pregnancy during the trial

Any volunteer who becomes pregnant during the trial will be followed up as per the protocol and until the end of the pregnancy. We will not routinely perform venepuncture in a pregnant volunteer.
The reason for withdrawal will be recorded in the CRF. Volunteers withdrawn from the trial may be replaced on the decision of the Investigator. If the volunteer is withdrawn due to an AE, the Investigator will arrange for appropriate specialist management or follow up visits or telephone calls until the AE has resolved or stabilised. The regulatory authorities will be informed in a timely manner. The extent of follow up after premature discontinuation will be determined by the Investigator but will be at least for the whole trial period, and if pregnant, until pregnancy outcome. An 'End of Study letter' will be sent to each withdrawn volunteer's GP.
If a volunteer withdraws from the trial, samples collected before their withdrawal from the trial will be used/stored unless the volunteer specifically requests otherwise. Long term safety data collection will continue as appropriate if a volunteer has received a challenge dose.

9.18Safety

Discontinuation of the trial
The trial will be discontinued in the event of any of the following:

- New scientific information is published to indicate that volunteers in the trial are being exposed to undue risks as a result of administration of the challenge agent, or as a result of the trial procedures or follow-up schedule.
- Serious concerns about the safety of the challenge agent arise as a result of one or more challenge-related SAE(s) occurring in the volunteers enrolled in this or any other ongoing trial of BCG.
- For any other reason at the discretion of the Investigator.

9.19End of Study Definition

The trial will be completed when the last volunteer enrolled into the trial has completed their final follow up visit. An end of study letter will be sent to each volunteer's GP.

10 CHALLENGE AGENT AND DEVICES

10.20 BCG Description

BCG SSI

BCG SSI contains live attenuated Danish strain 1331 Mycobacterium *bovis* BCG. It is supplied as a powder and solvent for suspension. Each vial contains 2-8 x 10^6 cfu (see SmPC).

BCG Bulgaria (InterVax)

BCG Bulgaria (Intervax) is a dried preparation containing live bacteria derived from an attenuated strain of Mycobacterium *bovis* BCG. It is supplied as a powder and diluent for suspension. Each vial contains 1.5-6 x 10^6 cfu (see SmPC).

Storage of BCG

BCG SSI will be supplied to the CCVTM in Oxford from the Churchill Hospital pharmacy. BCG Bulgaria (InterVax) will be shipped from the manufacturer InterVax in Bulgaria directly to the Clinical Biomanufacturing Facility (CBF) in the presence of a temperature logger. The vaccine will be re-labelled for local use in the TB041 trial.
Dispensed BCG vials are supplied in boxes containing multiple vials and each box is clearly labelled with the market product.
The challenge agent will be stored at +2 to +8°C (nominal temperature) in a secure, temperature-monitored refrigerator at the CCVTM, University of Oxford, Churchill Hospital.

10.21 Dispensing and administration

All movements of vials of the trial agent in or out of the locked refrigerator will be documented. Challenge agent accountability, storage, shipment and handling will be in accordance with local SOPs and other relevant local forms.
The challenge agent will be administered by ID injection over the deltoid region of the non-dominant upper arm, or by aerosol inhalation, according to the site-specific SOP. Volunteers will stay in the unit for 60 minutes (±10 minutes) after challenge. During the administration of the challenge agent, monitoring equipment, oxygen, medicines including bronchodilators and resuscitation equipment will be immediately available for the management of anaphylaxis and bronchospasm according to the site-specific SOP.
In order to minimise dissemination of the BCG bacteria into the environment, a number of measures will be instituted during and following challenge:

- The dilution process will be carried out in a sealed vial in accordance with the relevant SOP.
- The aerosol inhaled challenge will take place in a closed clinic room and not opened until 10 minutes after the end of nebulisation.
- During aerosol inhalation all clinical staff will wear appropriate protective clothing (PPE); gown, goggles, overshoes, gloves and FFP3 face mask. The volunteer will wear a hood in order to contain the aerosol.
- All disposable items including needles, vials, dressings, and protective clothing will be disposed of by following clinical waste and infection control policies, in accordance with the current approved SOP.
- Non-disposable parts of the MicroAIR NE-U22 nebuliser will be cleaned and disinfected in accordance with the device documentation.
- The clinic room will then undergo a terminal clean in accordance with local recommendations.

10.22 Saline

Sterile saline will be used for dilution (for BCG Bulgaria (InterVax) extra saline diluent will be supplied by the manufacturer) and placebo doses. The volume of saline used as placebo will be identical to the challenge volume by the same route.
Hypertonic saline will be delivered via nebuliser according to the site-specific SOP.

10.23 Salbutamol

Volunteers undergoing the induced sputum procedure will be pre-treated with a prescribed dose of salbutamol (200mcg delivered via spacer) in accordance with the European Respiratory Society's guidelines [33].

10.24 MicroAIR NE-U22

This nebuliser is used for aerosol delivery of the BCG. This is an approved electromedical device, CE0197, EAN code 40 15672 10142 1. Information about the specifications, usage, and maintenance of the nebuliser device can be found in the device documentation [34]. This is the same device used in studies TB026 and TB035.

10.25 Ultrasonic nebuliser NE-U780

This nebuliser is used for delivering the hypertonic saline during the induced sputum procedure. This is an approved electromedical device, CE0197, EN60601-1-2-2007. Information about the specifications, usage, and maintenance of the nebuliser device can be found in the device documentation [35].

10.26 Sedative & anaesthetic agents for bronchoscopy

Fentanyl is a licensed opioid used routinely to provide analgesia and sedation during medical procedures. It will be stored, dispensed and administered in accordance with standard NHS procedures and the Summary of Product Characteristics (SmPC).
Midazolam is a licensed benzodiazepine used routinely to provide sedation and amnesia during medical procedures. It will be stored, dispensed and administered in accordance with standard NHS procedures and the SmPC.
Lignocaine is a licensed local anaesthetic used routinely during medical procedures. It will be stored, dispensed and administered in accordance with standard NHS procedures and the SmPC.
Other licensed drugs may be used during bronchoscopy at the discretion of the Investigators.

11 ASSESSMENT OF SAFETY

Safety will be assessed by the frequency, incidence and nature of adverse events and serious adverse events arising during the trial. The safety profile of BCG given intradermally is well understood; therefore we will not be routinely collecting information regarding local reactions to ID administration (Groups 1D and 2F). We will however be recording other reactions since BCG is being given by a novel route (aerosol).

11.27 Interim Safety Review

Prior to dose escalation of the challenge agent, the clinical team and Chief Investigator will review the safety data and adverse events in volunteers before proceeding to the next challenge dose.
Dose escalation will not take place in the event of any of the following stopping criteria being met:

- **Any SAE deemed to be possibly, probably or definitely related to the challenge agent**
- **Solicited respiratory AEs:** If more than one dose of challenge agent (Groups 1A, 1B, 2A, 2B, 2C or 2D) are followed by the same Grade 3 solicited local AE beginning within 2 days after challenge (day of challenge and one subsequent day) and persisting at Grade 3 for >48 hours.
- **Solicited systemic AEs:** If more than one dose of challenge agent (Groups 1A, 1B, 2A, 2B, 2C or 2D) are followed by the same Grade 3 solicited systemic AE beginning within 2 days after challenge (day of challenge and one subsequent day) and persisting at Grade 3 for >48 hours.
- **Unsolicited AEs:** If more than one volunteer (Groups 1A, 1B, 2A, 2B, 2C or 2D) develops a Grade 3 unsolicited AE (including the same laboratory AE) that is considered possibly, probably or definitely related to challenge and persists at Grade 3 for >48 hours.

'Solicited respiratory AEs' and 'solicited systemic AEs' are those listed in Table 5 in Section 11.6. 'Unsolicited AEs' are any AE not described herein.
If a stopping rule has been met and following an internal safety review it is deemed appropriate to restart dosing, a request to restart dosing with pertinent data must be submitted to the regulatory authority as a request for a substantial amendment. The internal safety review will consider:

- The relationship of the AE or SAE to the challenge agent.
- The relationship of the AE or SAE to the challenge agent dose, or other possible causes of the event.
- If appropriate, additional screening or laboratory testing for other volunteers to identify those who may develop similar symptoms, and alterations to the current Participant Information Sheet (PIS).
- New, relevant safety information from ongoing research programs on the various components of the challenge agent.

The local ethics committee will also be notified if a holding rule is activated or released.

In addition to these pre-defined criteria, the study can be put on hold upon advice of the Local Safety Monitor, Chief Investigator, Study Sponsor, regulatory authority, Ethical Committee(s) or Local Safety Committee, for any single event or combination of multiple events which, in their professional opinion, jeopardise the safety of the volunteers or the reliability of the data.

11.28 Definitions

Adverse Event (AE)
An AE is any untoward medical occurrence in a volunteer, which may occur during or after administration of the challenge agent and does not necessarily have a causal relationship with the intervention. An AE can

therefore be any unfavourable and unintended sign (including an abnormal laboratory finding), symptom or disease temporally associated with the trial intervention, whether or not considered related to the trial intervention.
Each adverse event will be graded by the participant according to the table for grading severity of adverse events (see Section 11.7). Severity gradings may be reviewed and discussed with the participants at the clinic visits.

Adverse Reaction (AR)

An AR is any untoward or unintended response to the challenge agent. This means that a causal relationship between the agent and an AE is at least a reasonable possibility, i.e., the relationship cannot be ruled out. All cases judged by the reporting medical Investigator as having a reasonable suspected causal relationship to the challenge agent (i.e. possibly, probably or definitely related to it) will qualify as adverse reactions.

Unexpected Adverse Reaction

An adverse reaction, the nature or severity of which is not consistent with the applicable product information (e.g. SmPC).

Serious Adverse Event (SAE)

An SAE is an AE that results in any of the following outcomes, whether or not considered related to the trial intervention:

- Death.
- Life-threatening event (i.e., the volunteer was, in the view of the Investigator, at immediate risk of death from the event that occurred). This does not include an AE that, if it occurred in a more severe form, might have caused death.
- Persistent or significant disability or incapacity (i.e., substantial disruption of one's ability to carry out normal life functions).
- Hospitalisation or prolongation of existing hospitalisation, regardless of length of stay, even if it is a precautionary measure for continued observation. Hospitalisation (including inpatient or outpatient hospitalisation for an elective procedure) for a pre-existing condition that has not worsened unexpectedly does not constitute a serious AE.
- An important medical event (that may not cause death, be life threatening, or require hospitalisation) that may, based upon appropriate medical judgment, jeopardise the volunteer and/or require medical or surgical intervention to prevent one of the outcomes listed above. Examples of such medical events include allergic reaction requiring intensive treatment in an emergency room or clinic, blood dyscrasias, or convulsions that do not result in inpatient hospitalisation.
- Congenital anomaly or birth defect.

Serious Adverse Reaction (SAR)

An adverse event (expected or unexpected) that is both serious and, in the opinion of the reporting Investigator or Sponsors, believed to be possibly, probably or definitely due to the challenge agent or any other trial treatments, based on the information provided.

Suspected Unexpected Serious Adverse Reaction (SUSAR)

A serious adverse reaction, the nature and severity of which is not consistent with the information about the medicinal product in question set out in the SmPC.
NB: To avoid confusion or misunderstanding the following note of clarification is provided: "Severe" is often used to describe intensity of a specific event, which may be of relatively minor medical significance. "Seriousness" is the regulatory definition supplied above.

11.29 Foreseeable Adverse Reactions

Foreseeable adverse reactions are listed in section 6.3.

11.30 Expected Serious Adverse Events

No serious adverse events are expected in this trial.

11.31 Causality Assessment

For every unsolicited AE, an assessment of the relationship of the event to the administration of the challenge agent will be undertaken. An intervention-related AE refers to an AE for which there is a probable or definite relationship to administration of a challenge agent. An interpretation of the causal relationship of the intervention to the AE in question will be made, based on the type of event, the relationship of the event to the time of challenge administration and the known biology of the challenge agent, with reference to the SmPC (Table 4). Causality assessment will take place during interim analyses and at the final safety analysis.

0	**No Relationship**	No temporal relationship to trial product; ***and***<br>Alternate aetiology (clinical state, environment or other interventions); ***and***<br>Does not follow known pattern of response to challenge
1	**Unlikely**	Unlikely temporal relationship to trial product; ***and***<br>Alternate aetiology likely (clinical state, environment or other interventions); ***and***<br>Does not follow known typical or plausible pattern of response to challenge
2	**Possible**	Reasonable temporal relationship to trial product; ***or***<br>Event not readily produced by clinical state, environment or other interventions; ***or***<br>Similar pattern of response to that seen with other challenge agents
3	**Probable**	Reasonable temporal relationship to trial product; ***and***<br>Event not readily produced by clinical state, environment or other interventions; ***and***<br>Known pattern of response seen with other challenge agents
4	**Definite**	Reasonable temporal relationship to trial product; ***and***<br>Event not readily produced by clinical state, environment or other interventions; ***and***<br>Known pattern of response seen with the challenge agent

Table 4. Guidelines for assessing the relationship of challenge administration to an AE

11.32 Reporting Procedures for All Adverse Events

All AEs occurring in the 28 days following challenge observed by the Investigator or reported by the volunteer, whether or not attributed to trial medication, will be recorded on electronic diary cards. Data from the diary cards will be extracted following the last volunteer last visit (LVLV). Outside the diary card periods, respiratory and systemic AEs (listed in Table 5 below) will be specifically solicited at each visit, and graded by severity (as detailed in section 11.7). All AEs starting after the diary card period, or persisting after this period, will be recorded in the AE line listing of the CRF.
The overall appearance of the lung mucosa will be assessed at the day 14 bronchoscopy.
All AEs that result in a volunteer's withdrawal from the trial will be followed up until a satisfactory resolution occurs, or until a non-trial related causality is assigned (if the volunteer consents to this). Serious adverse events (SAEs) will be collected throughout the entire trial period.

	**Adverse event**
**Respiratory**	Cough
	Sore throat
	Wheeze
	Shortness of breath
	Coughing up phlegm
	Coughing up blood
	Chest pain
**Systemic**	Documented fever (oral temperature > 37.5° C)
	Myalgia
	Arthralgia
	Feverishness
	Headache
	Fatigue
	Nausea
	Malaise

Table 5. Routinely solicited adverse events

Reporting Procedures for Serious AEs (see Safety Reporting SOP)
In order to comply with current regulations on serious adverse event reporting to regulatory authorities, the event will be documented accurately and notification deadlines respected. SAEs will be reported on the SAE forms to members of the trial team immediately, once the Investigators become aware of their occurrence, as described in the site-specific SOP. Copies of all reports will be forwarded for review to the Chief Investigator and Principal Investigator (as the Sponsor's representatives) within 24 hours of the Investigator being aware of the suspected SAE. The local safety committee (LSC) will be notified of SAEs which are deemed possibly, probably or definitely related to trial interventions; the LSC will be notified immediately (within 24 hours) of the Investigators' being aware of their occurrence. SAEs will not normally be reported immediately to the ethical committee(s) unless there is a clinically important increase in occurrence rate, an unexpected outcome, or a new event that is likely to affect safety of trial volunteers, at the discretion of the Chief Investigator and/or LSC.

Reporting Procedures for SARs
These will be reported as per any SAE.

Reporting Procedures for SUSARs
The Chief Investigator will report all SUSARs to the MHRA and ethical committee(s) within required timelines (15 days for all SUSARs, unless life threatening in which case 7 days, with a final report within a further 8 days (total 15)). The Chief Investigator will also inform all Investigators concerned of relevant information about SUSARs that could adversely affect the safety of participants.
All SUSARs and deaths occurring during the trial will be reported to the Sponsor. For all deaths, available autopsy reports and relevant medical reports will be made available for reporting to the relevant authorities.

11.33 Assessment of Severity

The severity of clinical AEs will be assessed according to the scale in Tables 5-6:

	Grade 1 (mild)	Grade 2 (moderate)	Grade 3 (severe)
Fever (oral)	37.6°C - 38.0°C	38.1°C – 39.0°C	>39.0°C
Tachycardia (bpm)*	101 - 115	116 – 130	>130
Bradycardia (bpm)**	50 – 54	40 – 49	<40
Systolic hypertension (mmHg)	141 - 159	160 – 179	≥180
Diastolic hypertension (mmHg)	91 - 99	100 – 109	≥110
Systolic hypotension (mmHg)***	85 - 89	80 – 84	<80

Table 6. Severity grading criteria for physical observations.

*Taken after ≥10 minutes at rest; **When resting heart rate is between 60 – 100 beats per minute. Use clinical judgement when characterising bradycardia among some healthy volunteer populations, for example, conditioned athletes; ***Only if symptomatic (e.g. dizzy/ light-headed)

**GRADE 0**	None
**GRADE 1**	Mild: Transient or mild discomfort (< 48 hours); no medical intervention/therapy required
**GRADE 2**	Moderate: Mild to moderate limitation in activity – some assistance may be needed; no or minimal medical intervention/therapy required
**GRADE 3**	Severe: Marked limitation in activity, some assistance usually required; medical intervention/therapy required, hospitalisation possible

Table 7. Severity grading criteria for respiratory and systemic AEs

11.34 Procedures to be followed in the event of abnormal findings

Eligibility for enrolment in the trial in terms of laboratory findings will be assessed as detailed in Section 9.4. Abnormal clinical findings from medical history, examination or blood tests will be assessed as to their clinical significance throughout the trial. Laboratory adverse events will be assessed using the site-specific tables in the Site Master File/TMF. If a test is deemed clinically significant, it may be repeated, to ensure it is not a single occurrence. If a test remains clinically significant, the volunteer will be informed and appropriate medical care arranged as appropriate and with the permission of the volunteer. Specific details regarding findings, discussion with volunteers and resulting actions will be recorded in the CRF. Decisions to exclude the volunteer from enrolling in the trial or to withdraw a volunteer from the trial will be at the discretion of the Investigator.

11.35 Local Safety Committee

A Local Safety Committee (LSC) will be appointed to provide real-time safety oversight. The LSC will be notified within 24 hours of the Investigators' being aware of the occurrence of SAEs. The LSC has the power to place the trial on hold if deemed necessary following a trial intervention-related SAE. At the time of writing the LSC will be chaired by Professor Brian Angus, Clinical Tutor in Medicine, Honorary Consultant Physician and Director of the Oxford Centre for Clinical Tropical Medicine at Oxford University. There will be a minimum of two other appropriately qualified committee members. All correspondence between Investigator and LSC will be conveyed by the Investigator to the trial Sponsor. The Chair of the LSC will be contacted for advice and independent review by the Investigator or trial Sponsor in the following situations:

- Following any SAE deemed to be possibly, probably, or definitely related to the trial challenge agent.
- Any other situation where the Investigator or trial Sponsor feels independent advice or review is important.

Safety Profile Review

The safety profile will be assessed on an on-going basis by the Investigators. The Chief Investigator, Principal Investigator, and relevant Investigators (as per the trial delegation log) will also review safety issues and SAEs as they arise.

12 STATISTICS

This is primarily an exploratory and safety trial with descriptive endpoints. For Arm 1 of the trial, following the first six volunteers enrolled into Groups 1A and 1B, 12 volunteers will be recruited into each experimental group of the trial (Groups 1C and 1D). Similarly for Arm 2, following enrolment of the first 12 volunteers into Groups 2A, 2B, 2C and 2D, subsequent volunteers will be randomised into Groups 2E (9 volunteers) and 2F (12 volunteers); data from volunteers given the optimal dose selected for 2E (ie 2C or 2D) will be analysed together with those from that optimal dose Group (2C or 2D); total 12. Our previous experience with clinical studies suggests that this sample size is a feasible number to recruit, screen, enrol, and follow up in practical terms, whilst also allowing the determination of any substantial differences in the outcome measures between the two experimental groups in each Arm. The sample size has not been determined with the aim of achieving statistical significance.
Differences between means or medians between the two groups will be presented along with their 95% confidence intervals. Within each group, paired data will be analysed using either a paired t-test or the Wilcoxon matched pairs test depending on the normality of the data.

13 DATA MANAGEMENT

13.36 Source Data

Source documents are where data are first recorded, and from which participants' CRF data are obtained. These include the CRF itself (history and examination), the volunteer consent form, blood and microbiology results, radiology report, GP response letter, copy of bronchoscopy report and any further correspondence relating to the volunteer regarding medical/clinical issues. The CRF will be electronic and paper (CCVTM/OUH) or paper (NIHR-WTCRF) together with electronic diary cards (both sites).
CRF entries will be considered source data if the CRF is the site of the original recording (e.g. there is no other written or electronic record of data). All documents will be stored safely in confidential conditions. On all trial-specific documents, other than the signed consent, the participant will be referred to by the trial participant number/code, not by name.

13.37 Access to Data

Direct access will be granted to authorised representatives from the Sponsor, host institution and the regulatory authorities to permit trial-related monitoring, audits and inspections. All information relating to the trial and its volunteers will be held in strict confidence, and in accordance with GCP and institutional requirements.

13.38 Data Recording and Record Keeping

The CI will be responsible for collecting, recording, analysing, and storing all the data accruing from the trial. These tasks may be delegated to other Investigators. Paper CRFs will be stored in a key-locked cabinet at the NIHR-WTCRF and CCVTM, and electronic CRFs on the OpenClinica™ database, which is stored electronically on secure servers that are outsourced by OpenClinica^{TM}. Data from paper CRFs will be transcribed onto the OpenClinica^{TM} database. Some data may be duplicated anonymously into an electronic Microsoft Excel™ file on the CCVTM secure server for clinical monitoring through the trial.
Trial records will be held by the Investigator for as long as required by legislation as a minimum (currently until at least 2 years after the last marketing authorisation for the product) and in order to enable dissemination of trial results after publication, and to enable decoding and destruction of anonymised samples if subsequently requested by a volunteer. Data will subsequently be transferred to a secure archive in accordance with the Data Protection Act. Volunteers will be assigned individual unique trial numbers for identification on all trial records, except where the use of identifiable information is unavoidable (including on GP correspondence, registration documents, and consent forms).

14 QUALITY ASSURANCE PROCEDURES

14.39 Quality Assurance

Investigator procedures
Approved site-specific SOPs will be used at all clinical and laboratory sites.
Modification to protocol
No substantial amendments to this protocol will be made without consultation with, and agreement of, the Sponsor. Any substantial amendments to the trial that appear necessary during the course of the trial must be discussed by the Investigator and Sponsor concurrently. If agreement is reached concerning the need for an

amendment, it will be produced in writing by the CI and will be made a formal part of the protocol following ethical and regulatory approval.
An administrative change to the protocol is one that modifies administrative and logistical aspects of a protocol but does not affect the volunteers' safety, the scientific value of the trial, the conduct of the trial or safety of the challenge agent. An administrative change is a non-substantial amendment and does not require REC approval.
The CI is responsible for ensuring that changes to an approved trial, during the period for which REC approval has already been given, are not initiated without REC review and approval except to eliminate apparent immediate hazards to the volunteer.

Protocol deviation

All deviations from the protocol will be documented in a protocol deviation form and filed in the trial master file and site file accordingly.

14.40 Monitoring

Data will be evaluated for compliance with the protocol and accuracy in relation to source documents. The trial will be conducted in accordance with procedures identified in the protocol. Regular monitoring will be performed according to ICH GCP. According to applicable SOPs, the Monitors will verify that the clinical trial is initiated, conducted and completed, and data are generated, documented and reported in compliance with the protocol, GCP and the applicable regulatory requirements.

15 SERIOUS BREACHES

The Medicines for Human Use (Clinical Trials) Regulations contain a requirement for the notification of "serious breaches" to the MHRA within 7 days of the Sponsor becoming aware of the breach. A serious breach is defined as “A breach of GCP or the trial protocol which is likely to affect to a significant degree:

- the safety or physical or mental integrity of the volunteers of the trial
- the scientific value of the trial”

In the event that a serious breach is suspected the Sponsor must be contacted within 1 working day. In collaboration with the CI, the serious breach will be reviewed by the Sponsor and, if appropriate, the Sponsor will report it to the REC committee, Regulatory Authority and the NHS host organisation within 7 calendar days.

16 ETHICAL AND REGULATORY CONSIDERATIONS

16.41 Declaration of Helsinki

The Investigator will ensure that this trial is conducted in accordance with the principles of the Declaration of Helsinki as agreed by the World Medical Association General Assembly (Washington 2002).

16.42 Good Clinical Practice

The Investigator will ensure that this trial is conducted in accordance with ICH Good Clinical Practice (GCP), and local regulatory requirements.

16.43 Approvals

A copy of the protocol, proposed informed consent form, other written volunteer information and the proposed advertising material will be submitted to an independent REC for written approval. The Investigators will submit and, where necessary, obtain approval from the REC for all subsequent substantial amendments to the protocol and informed consent document. The Investigators will notify deviations from the protocol or SAEs occurring at the site to the Sponsor and will notify the REC of these in accordance with local procedures.

16.44 Reporting

The CI shall submit once a year throughout the clinical trial, or on request, an Annual Progress Report to the REC, host organisation and Sponsor. In addition, an End of Study notification and final report will be submitted to the MHRA, the REC, host organisation and Sponsor. An annual DSUR will be submitted to the Regulatory Authority, if required by the MHRA.

16.45 Volunteer Confidentiality

The trial staff will ensure the volunteers' anonymity is maintained. All documents will be stored securely and only accessible by trial staff and authorised personnel. No information concerning the trial or the data will be released to any unauthorised third party, without prior written approval of the sponsor. The trial will comply with the Data Protection Act, which requires data to be anonymised as soon as it is practical to do so.

16.46 Expenses and Benefits

Volunteers will be compensated *pro rata* for their time, travel and for trial procedures while participating in the trial, amounting to a total of approximately £515-£635, depending on the exact number of visits, site of recruitment and whether any repeat or additional visits are necessary.

17 FINANCE AND INSURANCE

17.47 Funding

This trial will be financed by research grants from The Gates Foundation and The Wellcome Trust, held by Professor Helen McShane.

17.48 Indemnity

If any volunteer is harmed as a result of this trial, medical care will be provided under the NHS.
Negligent Harm
Indemnity and/or compensation for negligent harm arising specifically from an accidental injury for which the University is legally liable as the Research Sponsor will be covered by the University of Oxford.
Non-Negligent Harm
Indemnity and/or compensation for harm arising specifically from an accidental injury, and occurring as a consequence of the Research volunteers' participation in the trial for which the University is the Research Sponsor will be covered by the University of Oxford.

17.49 Insurance

The University has a specialist insurance policy in place - Newline Underwriting Management Ltd, at Lloyd's of London - which would operate in the event of any volunteer suffering harm as a result of their involvement in the research.

18 PUBLICATION POLICY

When the trial is complete, a manuscript describing the primary trial results will be written and published in a peer-reviewed, open access journal. International guidelines will be followed regarding authorship. There may also be secondary publications on more exploratory results.

19 REFERENCES

1. Dye, C., et al., *Consensus statement. Global burden of tuberculosis: estimated incidence, prevalence, and mortality by country. WHO Global Surveillance and Monitoring Project.* JAMA, 1999. **282**(7): p. 677-86.
2. WHO, *Global tuberculosis report 2014. http://apps.who.int/iris/bitstream/10665/137094/1/9789241564809_eng.pdf?ua=1 (accessed 03 March 2015)*, 2014.
3. Corbett, E.L., et al., *Tuberculosis in sub-Saharan Africa: opportunities, challenges, and change in the era of antiretroviral treatment.* Lancet, 2006. **367**(9514): p. 926-37.
4. Colditz, G.A., et al., *Efficacy of BCG vaccine in the prevention of tuberculosis. Meta-analysis of the published literature.* JAMA, 1994. **271**(9): p. 698-702.
5. Fine, P.E., *The BCG story: lessons from the past and implications for the future.* Rev Infect Dis, 1989. **11 Suppl 2**: p. S353-9.
6. Trunz, B.B., P. Fine, and C. Dye, *Effect of BCG vaccination on childhood tuberculous meningitis and miliary tuberculosis worldwide: a meta-analysis and assessment of cost-effectiveness.* Lancet, 2006. **367**(9517): p. 1173-80.
7. Rodrigues, L.C., V.K. Diwan, and J.G. Wheeler, *Protective effect of BCG against tuberculous meningitis and miliary tuberculosis: a meta-analysis.* Int J Epidemiol, 1993. **22**(6): p. 1154-8.
8. Tameris, M.D., et al., *Safety and efficacy of MVA85A, a new tuberculosis vaccine, in infants previously vaccinated with BCG: a randomised, placebo-controlled phase 2b trial.* Lancet, 2013. **381**(9871): p. 1021-8.
9. Ndiaye, B.P., et al., *Safety, immunogenicity, and efficacy of the candidate tuberculosis vaccine MVA85A in healthy adults infected with HIV-1: a randomised, placebo-controlled, phase 2 trial.* Lancet Respir Med, 2015. **3**(3): p. 190-200.
10. Beveridge, N.E., et al., *A comparison of IFNgamma detection methods used in tuberculosis vaccine trials.* Tuberculosis (Edinb), 2008. **88**(6): p. 631-40.
11. Cooper, A.M., et al., *Disseminated tuberculosis in interferon gamma gene-disrupted mice.* J Exp Med, 1993. **178**(6): p. 2243-7.
12. Jouanguy, E., et al., *Partial interferon-gamma receptor 1 deficiency in a child with tuberculoid bacillus Calmette-Guerin infection and a sibling with clinical tuberculosis.* J Clin Invest, 1997. **100**(11): p. 2658-64.
13. Bonecini-Almeida, M.G., et al., *Induction of in vitro human macrophage anti-Mycobacterium tuberculosis activity: requirement for IFN-gamma and primed lymphocytes.* J Immunol, 1998. **160**(9): p. 4490-9.
14. Tameris., e.a., *Safety and efficacy of MVA85A, a new tuberculosis vaccine, in infants previously vaccinated with BCG: a randomised, placebo-controlled phase 2b trial.* Lancet, 2013.
15. Minassian, A.M., et al., *A human challenge model for Mycobacterium tuberculosis using Mycobacterium bovis bacille Calmette-Guerin.* J Infect Dis, 2012. **205**(7): p. 1035-42.
16. Harris, S.A., et al., *Evaluation of a human BCG challenge model to assess antimycobacterial immunity induced by BCG and a candidate tuberculosis vaccine, MVA85A, alone and in combination.* J Infect Dis, 2014. **209**(8): p. 1259-68.
17. Minhinnick, A., et al., *Optimization of a Human Bacille Calmette-Guerin Challenge Model: A Tool to Evaluate Antimycobacterial Immunity.* J Infect Dis, 2016. **213**(5): p. 824-30.
18. Hart, P.D. and I. Sutherland, *BCG and vole bacillus vaccines in the prevention of tuberculosis in adolescence and early adult life.* Br Med J, 1977. **2**(6082): p. 293-5.
19. Matsumiya, M., et al., *Gene expression and cytokine profile correlate with mycobacterial growth in a human BCG challenge model.* J Infect Dis, 2015. **211**(9): p. 1499-509.
20. Cusumano, C.L., J.A. Jernigan, and R.H. Waldman, *Aerosolized BCG (Tice strain) treatment of bronchogenic carcinoma: phase I study.* J Natl Cancer Inst, 1975. **55**(2): p. 275-9.
21. Garner, F.B., et al., *Aerosol BCG treatment of carcinoma metastatic to the lung: a phase I study.* Cancer, 1975. **35**(4): p. 1088-94.
22. WHO, *IVR strategic plan 2010 - 2020. http://whqlibdoc.who.int/hq/2010/WHO_IVB_10.02_eng.pdf?ua=1 (accessed 02 March 2015)* 2010.
23. Rosenthal, S.R., J.T. McEnery, and N. Raisys, *Aerogenic BCG vaccination against tuberculosis in animal and human subjects.* J Asthma Res, 1968. **5**(4): p. 309-23.
24. Nuermberger, E.L., et al., *Paucibacillary tuberculosis in mice after prior aerosol immunization with Mycobacterium bovis BCG.* Infect Immun, 2004. **72**(2): p. 1065-71.
25. White, A.D., et al., *Evaluation of the Immunogenicity of Mycobacterium bovis BCG Delivered by Aerosol to the Lungs of Macaques.* Clin Vaccine Immunol, 2015. **22**(9): p. 992-1003.

26. Ho, L.P., et al., *Reduced interleukin-18 levels in BAL specimens from patients with asthma compared to patients with sarcoidosis and healthy control subjects.* Chest, 2002. **121**(5): p. 1421-6.
27. Santosuosso, M., et al., *Mucosal luminal manipulation of T cell geography switches on protective efficacy by otherwise ineffective parenteral genetic immunization.* J Immunol, 2007. **178**(4): p. 2387-95.
28. White, A.D., et al., *Evaluation of the safety and immunogenicity of a candidate tuberculosis vaccine, MVA85A, delivered by aerosol to the lungs of macaques.* Clin Vaccine Immunol, 2013. **20**(5): p. 663-72.
29. Satti, I., et al., *Safety and immunogenicity of a candidate tuberculosis vaccine MVA85A delivered by aerosol in BCG-vaccinated healthy adults: a phase 1, double-blind, randomised controlled trial.* Lancet Infect Dis, 2014. **14**(10): p. 939-46.
30. Kariyawasam, H.H., et al., *Safety and tolerability of three consecutive bronchoscopies after allergen challenge in volunteers with mild asthma.* Thorax, 2007. **62**(6): p. 557-8.
31. Elston, W.J., et al., *Safety of research bronchoscopy, biopsy and bronchoalveolar lavage in asthma.* Eur Respir J, 2004. **24**(3): p. 375-7.
32. Busse, W.W., et al., *Investigative bronchoprovocation and bronchoscopy in airway diseases.* Am J Respir Crit Care Med, 2005. **172**(7): p. 807-16.
33. Zsoka, W, & Ildiko, H. *Induced sputum analysis: step by step*. Breathe, 2013; **9**: p.300-6
34. OMRON, *OMRON Microair U22 website* http://www.omron-healthcare.com/eu/en/our-products/respiratory-therapy/microair-u22 *(accessed 02 March 2015).* 2015.
35. OMRON, *OMRON ultrasonic nebulizer NE-U780 website* https://www.omron-healthcare.com/.../***u780***-*instruction-manual-ne-**u780**-e-en (accessed 11 October 2017).* 2017.
